# Deep learning‐based MRI analysis reveals Lewy body co‐pathology accelerates brain aging in Alzheimer's disease

**DOI:** 10.1002/alz.71593

**Published:** 2026-07-15

**Authors:** Babak Ahmadi, Melissa Armstrong, Breton M. Asken, Mostafa Reisi‐Gahrooei, Abbas Babajani‐Feremi, the Alzheimer's Disease Neuroimaging Initiative, the Australian Imaging Biomarkers and Lifestyle flagship study of ageing

**Affiliations:** ^1^ Department of Industrial and Systems Engineering University of Florida Gainesville Florida USA; ^2^ Department of Neurology University of Florida Gainesville Florida USA; ^3^ Magnetoencephalography (MEG) Lab The Norman Fixel Institute of Neurological Diseases University of Florida Health Gainesville Florida USA; ^4^ Department of Clinical and Health Psychology University of Florida Gainesville Florida USA

**Keywords:** α‐synuclein, Alzheimer's disease, brain age, brain age gap, cognitive decline, cognitive impairment, co‐pathology, deep learning, Lewy body, MRI, neurodegeneration, neurodegenerative burden

## Abstract

**INTRODUCTION:**

Lewy body (LB) co‐pathology is common in Alzheimer's disease (AD), yet its in vivo impact on neurodegeneration remains unclear.

**METHODS:**

We trained a 3D‐DenseNet on multi‐cohort T1‐weighted magnetic resonance imaging (MRI) from cognitively unimpaired individuals to estimate brain age (BA) and applied it to cognitively impaired Alzheimer's Disease Neuroimaging Initiative (ADNI) participants stratified by cerebrospinal fluid (CSF) p‐tau_181_/Aβ_42_ (AD) and α‐synuclein seed amplification assay (LB) status into: AD+LB+, AD+LB−, AD−LB+, and AD−LB−. We compared baseline and longitudinal brain–age gap (BAG), identified saliency‐derived anatomical contributors, assessed regional atrophy and cognition, and evaluated whether BAG explains LB‐related clinical decline within AD.

**RESULTS:**

The model robustly captured normative aging. AD+LB+ exhibited the greatest and fastest‐increasing BAG, with saliency maps emphasizing regions that also showed steeper longitudinal atrophy and concordant cognitive decline, and BAG mediated a substantial portion of LB‐related cognitive impairment within AD+.

**DISCUSSION:**

LB co‐pathology confers an additional neurodegenerative burden in AD, underscoring the importance of combined biomarker assessment and targeted interventions.

## Highlights

- Brain age model robustly captured normative aging on a held‐out test set (*r *= 0.92, *R* ^2 ^= 0.88).
- Alzheimer's disease positive and Lewy body positive status (AD+LB+) showed the highest baseline and fastest‐rising brain–age gap (BAG) versus single‐pathology groups.
- Female BAG vulnerability in AD+ was further intensified by LB co‐pathology.
- Deep learning (DL) ‐driven saliency maps in AD+LB+ highlighted both canonical AD‐vulnerable and LB‐sensitive regions.
- BAG partially explained LB‐related cognitive impairment within AD+.

## 1 BACKGROUND

Alzheimer's disease (AD) and Lewy body (LB) disease are common neurodegenerative disorders in older adults.^1^ AD is characterized by the accumulation of amyloid‐beta (Aβ) plaques and tau neurofibrillary tangles.^2^ LB disease, manifesting clinically as Parkinson's disease and dementia with Lewy bodies (DLBs), is characterized by intraneuronal aggregation of misfolded α‐synuclein proteins that form Lewy bodies and Lewy neurites.^3^, ^4^ Although α‐synuclein accumulation is the hallmark of LB diseases, it is also a common co‐pathology in AD.^2^, ^5^, ^6^ Post‐mortem studies have shown that approximately 30%–50% of autopsy‐confirmed AD cases exhibit comorbid α‐synuclein pathology in addition to Aβ and tau depositions.^7^, ^8^, ^9^, ^10^ This overlap suggests that mixed pathology may accelerate neurodegeneration and cognitive decline beyond either condition alone,^2^, ^5^, ^11^, ^12^, ^13^, ^14^ thereby increasing diagnostic and therapeutic complexity. Therefore, in vivo detection of co‐pathologies is critical for prognosis and targeted therapy.

AD pathology biomarkers are increasingly used in research and clinical care, including cerebrospinal fluid (CSF) biomarkers, Aβ positron emission tomography (PET), and emerging blood‐based assays.^15^ Recent advancements in CSF α‐synuclein seed amplification assay (SAA) have enabled in vivo detection of LB pathology.^16^, ^17^, ^18^ Across clinical^19^, ^20^ and neuropathological^2^, ^5^, ^21^, ^22^, ^23^ cohorts, SAA demonstrated high specificity and sensitivity, providing a vital in vivo tool for investigating the role of LB pathology across neurodegenerative diseases.

Building on these advancements, SAA positivity has been linked to earlier symptom onset, faster cognitive decline, and greater cortical hypometabolism, especially when co‐existing with AD pathology.^2^, ^24^ Notably, it has also been associated with structural atrophy in specific regions like the nucleus basalis of Meynert (NBM).^3^ However, prior structural investigations have largely been cross‐sectional and region‑delimited, without simultaneously mapping SAA positivity on an AD background onto distributed structural patterns and to both baseline status and subsequent cognitive change. Therefore, there remains a need for an integrated, longitudinal, biomarker‑anchored approach that characterizes the global and region‐specific structural footprint of LB co‐pathology, thereby testing whether neuroanatomical burden statistically bridges co‑pathology and cognitive impairment.

In parallel, advances in deep learning (DL) have opened new avenues in neurodegenerative research, including its integration with neuroimaging,^25^, ^26^, ^27^, ^28^, ^29^, ^30^, ^31^ such as magnetic resonance imaging (MRI). DL applied to T1‐weighted MRI can yield biomarkers for neurodegeneration; chief among these biomarkers is the brain–age gap (BAG)—the difference between a DL‐predicted brain age (BA) and chronological age (CA).^28^, ^32^, ^33^, ^34^ Notably, multiple studies have demonstrated robust performance of BA models and associated the greater BAGs to worse clinical outcomes,^25^, ^29^, ^35^, ^36^, ^37^, ^38^ supporting BAG as a measure of neurodegenerative burden.

Prior studies have shown that BAG is elevated in AD or clinically diagnosed LB disease versus cognitively unimpaired (CU) individuals.^25^, ^26^, ^32^ However, they have largely focused on isolated neurodegenerative diseases and LB studies have relied on clinical criteria (e.g., consensus guidelines for DLB^39^) rather than detection of misfolded α‐synuclein. Consequently, no work to date has investigated whether in vivo LB pathology contributes to BAG and, more critically, whether its combination with AD yields larger and faster‐increasing BAG and neurodegeneration beyond AD alone. This gap is notable since LB pathology often contributes to structural decline patterns beyond hallmark AD atrophy.^26^ Prior investigations of α‐synuclein‐associated atrophy have also shown inconsistent findings regarding its overlap with AD‐vulnerable regions (e.g., the medial temporal lobe [MTL]).^3^, ^40^, ^41^, ^42^, ^43^ Equally missing is an MRI‐based longitudinal perspective on how LB pathology compounds over time, particularly against the backdrop of AD, and how it ultimately influences region‐specific and overall neurodegeneration, with downstream effects on cognition.

**RESEARCH IN CONTEXT**

1. **Systematic review**: Literature searches (e.g., PubMed) showed that α‐synuclein seed amplification assay (SAA) detects Lewy body (LB) pathology and that SAA‐positivity in Alzheimer's disease (AD) is associated with faster clinical decline, whereas magnetic resonance imaging (MRI) signatures are inconsistent and mostly cross‐sectional. These works are appropriately cited. No study has yet paired SAA‐based LB detection with longitudinal MRI and cognitive outcomes to characterize the distributed structural signature of co‐pathology and its relationship to faster disease progression in AD.
2. **Interpretation**: Leveraging in vivo cerebrospinal fluid (CSF) biomarkers, we show that AD+LB+ exhibits the greatest and fastest increase in BAG (used as a global index of neurodegenerative burden). Model saliency localized this excess burden to canonical AD‐vulnerable as well as additional non‐canonical regions, motivating targeted region of interest (ROI) analyses. These saliency‐informed regions showed greater baseline atrophy and steeper tissue loss under co‐pathology, alongside concordant cognitive decline. BAG partially mediates LB‐related cognitive impairment within AD+ and suggests that female vulnerability associated with AD positivity is further amplified by LB co‐pathology.
3. **Future directions**: Replicate in independent/diverse cohorts including Parkinson's disease (PD)/DLB; test multimodal imaging/blood biomarkers; and clarify mechanisms of the sex‐by‐LB co‐pathology effect.

**FIGURE 1:**
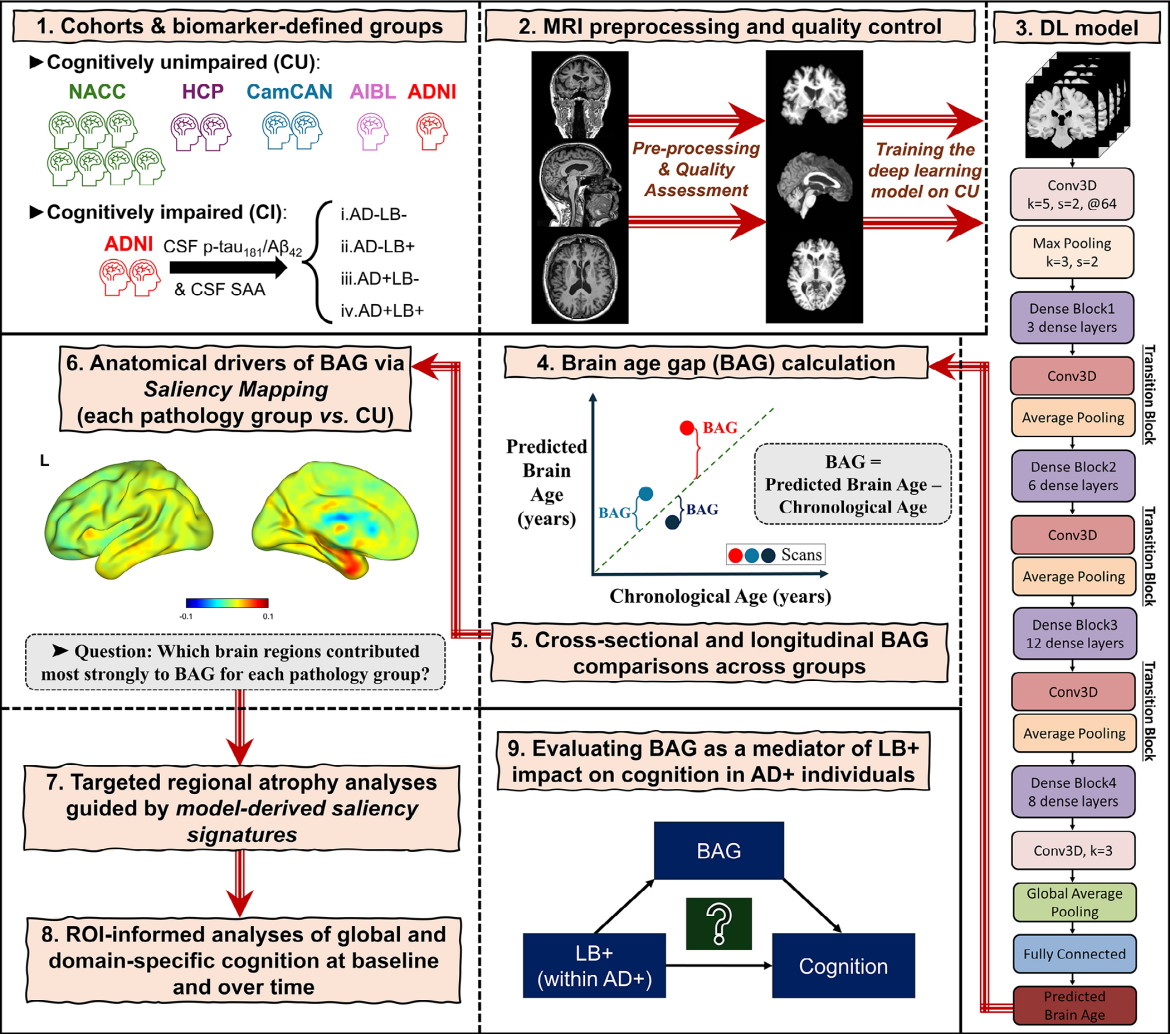
An overview of the study design and analytic workflow. (1) T1‐Weighted MRI scans were collected from five major cohorts (NACC, HCP, CamCAN, AIBL, and ADNI; each symbol represents ∼350 participants), providing cognitively unimpaired (CU) scans for training the deep‐learning (DL) model, plus cognitively impaired participants with biomarker‐defined Alzheimer's disease (AD) and Lewy body (LB) pathology. (2) All MRI scans underwent standardized preprocessing (FreeSurfer, affine registration to the 2‐mm MNI152 template) and rigorous quality assessments to prepare the data for the DL model. (3) A 3D‐DenseNet architecture was trained on CU scans and was used to predict brain age in all pathology subgroups. (4) Brain–age gap (BAG) calculation (predicted brain age−chronological age), providing a global index of deviation from normative aging. (5) Covariate‐adjusted group comparisons at baseline and over time test BAG differences across groups. (6) Model interpretation via saliency maps (each pathology group vs. CU) that visualize model sensitivity and highlight anatomy contributing to the model's brain age predictions in a group‑specific manner. (7) Saliency‐guided ROI volumetrics test whether model‐identified regions show greater baseline differences and faster longitudinal atrophy. (8) ROI‐informed analyses of global and domain‐specific cognition evaluate clinical consequences in parallel with structural change. (9) Mediation analysis assesses whether BAG statistically mediates the effect of LB positivity within AD+ individuals on cognition. All statistical models are specified in Section 2 and adjust for prespecified covariates; saliency maps are used for interpretation and ROI selection rather than as direct evidence of tissue loss. AD, Alzheimer's disease; ADNI, Alzheimer's Disease Neuroimaging Initiative; AIBL, Australian Imaging, Biomarkers & Lifestyle; BAG, brain age gap; CamCAN, Cambridge Centre for Ageing and Neuroscience; CDR‐SB, Clinical dementia rating, sum of boxes; CI, cognitively impaired; CU, cognitively unimpaired; DL, deep learning; HCP‐A, Human Connectome Project in Aging; LB, Lewy body; MRI, magnetic resonance imaging; NACC, National Alzheimer's Coordinating Center; ROI, region of interest.

In this study, we established a DL‐based BA model validated across multiple cohorts to capture normal aging trajectories; used in vivo biomarkers of Aβ, tau, and α‐synuclein to evaluate how AD, LB, and AD+LB differ in BAG (including sex‐stratified analyses); applied DL saliency maps to identify anatomical drivers of elevated BAG and to guide hypothesis‐generating region of interest (ROI) selection; assessed the longitudinal volumetric trajectories of these saliency‐informed regions to determine how LB co‐pathology reshapes spatiotemporal brain atrophy and amplifies typical AD‐related neurodegeneration; and finally, tested whether BAG explains the association between LB co‐pathology and faster clinical deterioration. The study design is shown in Figure 1.

## 2 METHODS

### 2.1 Study cohort

The data analyzed in this study were obtained from five established cohorts—National Alzheimer's Coordinating Center (NACC), Alzheimer's Disease Neuroimaging Initiative (ADNI), Australian Imaging, Biomarkers & Lifestyle (AIBL),^44^ Cambridge Centre for Ageing and Neuroscience^45^, ^46^ (CamCAN), and Human Connectome Project in Aging (HCP‐A)—each operating under its own ethical oversight and approved data acquisition protocols, where in each, all participants provided appropriate informed consent. All studies received approval from their respective institutional review boards or equivalent ethics committees. To capture normal aging trajectories using our BA estimation model based on the MRI scans, we first selected CU individuals across cohorts: ADNI* (*n* = 348; 71.3 ± 6.2 years, 65.0% female), AIBL^44^ (*n* = 359; 71.8 ± 6.1 years, 61.8% female), NACC† (*n* = 2304; 68.6 ± 10.7 years, 68.3% female), CamCAN‡ (*n* = 623; 55.4 ± 17.9 years, 50.9% female),^45^, ^46^ and HCP‐A§ (*n* = 721; 60.2 ± 15.6 years, 56.2% female). To avoid data leakage and overfitting, only the first MRI scan per participant was included, yielding a total of 4355 scans from individuals aged 23–100 years.

Given the longitudinal nature of ADNI, many CU participants had serial scans acquired at different time points (on average, 5.0 ± 2.6 scans per participant). We excluded any participants that were used in the training phase from this cohort to avoid leakage, and leveraged the remaining longitudinal scans from the other CU individuals in ADNI (*n* = 139, 691 scans; mean age 72.6 ± 5.9 years; 55.4% female) both to evaluate the model's reliability in capturing normal aging trajectories within the same ADNI database—from which AD/LB pathology subgroups would later be derived—and to establish a reference subgroup for subsequent comparisons with those pathological cohorts.

We next identified four cognitively impaired (CI; ADNI clinician‐assigned diagnosis of MCI or dementia) AD/LB pathology subgroups from the ADNI cohort. Participants included in these subgroups had their α‐synuclein status determined via the most recent CSF α‐synuclein SAA test, along with available data on CSF Aβ_42_ and p‐tau_181_ within 1 year—biomarkers for Aβ and tau—and at least one T1‐weighted magnetic resonance imaging (MR scan taken within 1 year of the SAA test. CSF concentrations of Aβ_42_ and p‐tau_181_ were measured using the Elecsys CSF immunoassay.^47^ AD‐positivity was defined using the CSF p‐tau_181_/Aβ_42_ ratio based on a previously specified threshold of 0.021 against [18F]florbetapir amyloid‐PET, as recommended by the ADNI Biomarker Core Steering Committee. LB status was determined using the α‐synuclein SAA test, performed at the Amprion Clinical Laboratory and validated for clinical use in accordance with Clinical Laboratory Improvement Amendment (CLIA) requirements.^2^ In prior autopsy‐validated work using this assay, antemortem CSF α‐synuclein SAA showed 97.8% sensitivity and 98.1% specificity for limbic/neocortical α‐synuclein pathology, although sensitivity was substantially lower for amygdala‐predominant pathology (14.3%).^22^ In ADNI specifically, the same assay showed 79% sensitivity and 97% specificity overall for LB pathology, with higher sensitivity for neocortical (100%) than limbic (57%) or amygdala‐predominant (60%) pathology.^5^ Based on the results of the p‐tau_181_/Aβ_42_ ratio and SAA α‐synuclein status, participants were classified into one of four AD/LB pathology subgroups: AD+LB+ (*n* = 166; 74.3 ± 7.2 years, 39.2% female), AD+LB− (*n* = 396; 73.5 ± 7.0 years, 42.4% female), AD‐LB+ (*n* = 46; 73.8 ± 7.2 years, 28.3% female), and AD−LB− (*n* = 195; 72.0 ± 8.2 years, 40.5% female). The AD−LB− subgroup therefore represents cognitively impaired individuals without biomarker evidence of AD or LB pathology, in whom other or mixed etiologies may underlie clinical impairment. Participants with *indeterminate* SAA results (i.e., samples that did not meet the laboratory's positive/negative thresholds after repeat testing per the assay algorithm) were excluded (*n* = 9). Because ADNI excludes individuals presenting with parkinsonism or other overt non‐AD neurological disorders, the LB+ subgroups in this study represent CSF α‐synuclein SAA‐positive individuals without clinically diagnosed Parkinson's disease (PD) or dementia with Lewy bodies.

### 2.2 MRI data pre‐processing and quality control assessment

All raw T1‐weighted MRI scans were processed using FreeSurfer's recon‐all pipeline (FreeSurfer v 7.1.1), which provides fully automated skull‐stripping, motion correction, normalization of nonuniform signal intensities, and Talairach space transformation, as well as tissue segmentation and removal of nonbrain structures.^48^ FreeSurfer was selected due to its established reliability in the literature, its comprehensive and reproducible workflow, and its capacity to facilitate analyses of neuroanatomical structures. Following recon‐all, each MRI underwent an erosion–dilation step to refine tissue boundaries and was then affinely registered to the 2‐mm isotropic MNI152 template using Advanced Normalization Tools (ANTs) to achieve a consistent spatial framework for further analyses.

Ensuring the quality of the processed MRI data was critical, as the presence of low‐quality scans can bias model training and evaluation. Although Qoala‐T^49^ was used as an initial automated quality control tool, we complemented it with additional outlier detection methods to identify images that were not appropriately processed by FreeSurfer. These measures were implemented both before and after data normalization to capture a wide range of intensity‐ and structure‐related anomalies. Prior to normalization, we applied intensity‐based criteria to detect scans exhibiting extreme deviations in global brightness, contrast, and overall intensity distribution. For instance, we identified scans whose mean or standard deviation of voxel intensities substantially diverged from the dataset norm, as well as those whose voxel intensities, when standardized, fell significantly outside expected ranges. Such deviations often suggest technical issues or scanner‐specific artifacts that warrant exclusion or closer inspection. Following normalization, we shifted our focus toward detecting more subtle anomalies that would be less apparent in raw intensity space. We used principal component analysis (PCA) to represent each scan in a lower‐dimensional space capturing the major patterns of variability, and then measured each scan's distance from the multivariate center using the Mahalanobis metric. This approach facilitated the identification of images that, despite appearing normalized, still exhibited atypical structural or intensity profiles. In addition, we applied an Isolation Forest algorithm,^50^ which relies on iterative feature splitting, to identify scans that could be more easily isolated from the overall distribution, thus flagging subtle irregularities not explained by global intensity measures or principal components alone.

By integrating these checks before and after normalization, we established a multi‐layered quality control strategy. Notably, each outlier detection method was applied separately to each subgroup, rather than to the entire pooled dataset, to ensure that flagged scans represented true anomalies relative to their own clinical context. Following each round of outlier detection, all identified images were manually inspected to confirm that they were genuine outliers rather than artifacts of the detection process. This careful, stepwise approach ensured that only high‐quality, representative MRI data informed the subsequent stages of DL model training and validation, ultimately enhancing the robustness and interpretability of our results.

To assess robustness to recruitment source and site effects, we conducted pre‑specified sensitivity analyses with ComBat‑GAM.^51^ Harmonization parameters were learned only on the CU training set to avoid information leakage and then applied to validation, test, and all ADNI pathology groups. We evaluated reduction of site signal and preservation of biological signal using domain‑predictability (site classification after PCA), partial *R* ^2^ of site and age on principal components, and structural similarity (SSIM), and investigated whether predicted BAs and main findings differed from the unharmonized results. Details are provided in the Supporting Information.

### 2.3 DL model architecture and training

A 3D‐DenseNet architecture^52^ was chosen to predict BA from the processed T1‐weighted MRI scans of CU individuals, primarily due to its proven performance in handling such data.^26^ Additionally, we used a 3D‐DenseNet backbone to estimate BA because it (i) operates on whole‐brain T1 volumes without ROI selection or handcrafted features, preserving spatial information; (ii) supports gradient‐based saliency mapping to localize voxel‐wise contributors to the BAG (critical for our downstream anatomical analyses); and (iii) yielded the best predictive accuracy in internal benchmarking against other CNN‐based models. This combination of whole‐brain coverage, interpretability, and performance made 3D‐DenseNet the most appropriate backbone for detecting disease‐related deviations from normative aging. Model performance was also benchmarked against the widely adopted brainageR^53^ framework, evaluated on identical CU test splits across ADNI, AIBL, CamCAN, HCP‑A, and NACC. As depicted in Figure 1, the 3D‐DenseNet architecture comprises four dense blocks containing 3, 6, 12, and 8 layers, respectively, with each block separated by a transition block. Within each dense layer, a 1×1×1 bottleneck convolution is set to scale the current in‐channel dimension, followed by a 3×3×3 convolution. The initial convolution layer uses a 5×5×5 kernel, and after the final dense block, a 3×3×3 convolution is applied before a global average pooling layer. The resulting features are fed into a fully connected layer predicting a single scalar, called the BA. This architecture contains 251,098,737 trainable parameters. Because this is a relatively high‐capacity configuration, we additionally trained two shallower 3D‐DenseNet variants as sensitivity analyses, using identical preprocessing, data splits, optimization, and bias‐correction procedures while varying only the number of dense layers per block (2,4,8,6 and 2,3,6,4; approximately 111 and 60.5 M trainable parameters, respectively). The full model showed the best normative age prediction performance and was therefore retained as the primary model (details are provided in the Supporting Information, Section S5). Although 3D‐DenseNet architectures often rely on a smaller bottleneck dimension,^26^ we opted for a configuration in which we allow the 1×1×1 convolution to scale with the current in‐channel dimension, rather than a fixed multiple of the growth rate. This larger design was motivated by our diverse, multi‐cohort dataset, where we found that the additional capacity helped capture subtle inter‐subject variations and group differences without overfitting, as evidenced by steadily improving validation losses and final test mean absolute error (MAE). To further guard against overfitting and cohort‐specific artefacts, model development was restricted to one MRI scan per participant, used strict age‐stratified train/validation/test splitting, and was evaluated on an independent multicohort held‐out test set spanning all five source cohorts. Additional robustness analyses included Leave‐One‐Cohort‐Out (LOCO) cross‐validation, cohort‐matched benchmarking against brainageR, ComBat‐GAM site‐sensitivity analyses, and reduced‐capacity architectures sensitivity analyses, all provided in the Supporting Information.

Training proceeded in a staged manner, with five iterative cycles of 15 epochs each. Adam was chosen as the optimizer, and early stopping was applied to halt training if the validation loss did not improve for several epochs (patience = 6), ensuring that the model did not overfit. Extensive hyperparameter tuning was conducted to identify the optimal configuration for batch size, kernel sizes, growth rates, loss function, and learning rate, while the optimizer and the early stopping callback were kept constant throughout the tuning phase, providing a controlled environment to evaluate each combination. After systematically evaluating multiple configurations, we selected the best‐performing hyperparameters based on their validation set performance. The final choice included a mini‐batch size of 8, MAE loss function for directly quantifying prediction errors in years, and an initial learning rate of 5×10^−6^. Whenever the validation performance plateaued over all epochs of an iteration, we reduced the learning rate by a factor of 0.7 to facilitate convergence toward an optimal solution. Random seeds were fixed at 42 to enhance reproducibility, and during training, the best model state was periodically saved whenever validation performance improved, ensuring that we retained the optimal set of parameters for downstream evaluation. All training was performed in PyTorch using two A100 graphics processing units (GPUs) in parallel, allowing efficient handling of the large 3D volumes and substantial model complexity.

To ensure robust estimation of generalizable brain aging patterns, we assembled a cohort of 4355 CU participants, aged 23–100 years. To prevent data leakage and to rigorously assess generalizability, we split the dataset into training (80%, *n* = 3484; 65.8 ± 13.5 years, 63.6% female), validation (10%, *n* = 435; 66.3 ± 12.7 years, 61.4% female), and test (10%, *n* = 436; 66.2 ± 12.9 years, 61.7% female) sets. Splits were generated using stratified sampling based on discretized age bins, which preserved the age composition across subsets. Figure S1 shows the age composition of the training cohort and the age‐bin distributions across the training, validation, and test subsets, demonstrating that age‐stratified splitting preserved comparable age‐bin composition across subsets. We also checked that sex distribution did not differ significantly among the training, validation, and test sets, preserving comparability for subsequent analyses, including those involving sex effects. As mentioned before, we only included the first available MRI scan per participant, preventing any subject‐level overlap between the training, validation, and testing phases. By integrating a robust 3D‐DenseNet model, rigorously stratified data splitting, careful hyperparameter tuning, and adaptive learning rate and early stopping strategies, we aimed to achieve a stable, generalizable BA estimation pipeline. This carefully engineered training process ensures that the final model reflects genuine aging patterns rather than artifacts of sampling, preprocessing, or overfitting.

### 2.4 Saliency maps analysis

We employed a gradient‐based saliency mapping approach to visualize the spatial distribution of features that most strongly influenced the predictions of our 3D‐DenseNet model. Following the extension of previously established 2D saliency techniques to the 3D domain,^25^ each input image (volume $V_{0}$) was passed through the trained 3D‐DenseNet model with a score function S(V) and voxels in $V_{0}$ are ranked based on their importance to the score function. Although this score function is inherently nonlinear with respect to *V*, a local linear approximation around $V_{0}$ can be obtained using a first‐order Taylor expansion. In this approximation, voxel importance is inferred from the partial derivatives of S(V) with respect to the input V at $V_{0}$, reflecting how infinitesimal changes in voxel intensity affect the predicted BA. Notably, since gradients are computed with respect to each individual scan, the saliency maps are inherently input‐specific: they differ across participants according to their unique anatomical features and pathology, despite the network weights remaining fixed after training.

More specifically, the procedure for generating saliency maps was as follows. First, we fed each subject's MRI volume into the 3D‐DenseNet in evaluation mode in PyTorch, identified the output neuron with the highest contribution to the predicted BA, and then computed the gradients of that top‐scoring output with respect to the input volume. These gradients capture how sensitively the model's prediction responds to each voxel's intensity. To further enhance interpretability and mitigate noise, we applied Gaussian smoothing to the saliency maps. This additional step provided clearer, more coherent patterns of feature relevance across the 3D volume. Smoothed saliency maps were generated for all CU individuals as well as for all AD/LB participants, enabling a nuanced examination of how structural brain features differentially influenced the DL model's BA estimates.

Subsequently, we averaged individual saliency maps within each diagnostic group to obtain representative mean saliency patterns. To elucidate differences in salient patterns across groups, we computed difference maps by subtracting the averaged saliency maps of one cohort from another. These difference maps highlight the regions in the brain MRI volumes that differentially influence the model's prediction across distinct clinical conditions. In addition to the described vanilla gradients, we also computed SmoothGrad^54^ attributions to reduce high‑frequency noise by averaging gradient magnitudes over multiple Gaussian‑perturbed inputs. This complementary analysis was to ensure that our interpretability results were robust and not driven by reliance on a single attribution method. Group‑mean and difference maps were compared between methods at ROI and voxel levels, and results are provided in the Supporting Information. Notably, saliency maps indicate model sensitivity for age prediction and were used as a hypothesis‑generating tool; they were not interpreted as direct evidence of regional atrophy or disease‐specific vulnerability. All surfaces were visualized using the BrainNet Viewer.^55^

### 2.5 Neuroanatomical assessments

To examine the impact of co‐pathology on regional brain atrophy, we used the volumetric measures extracted from FreeSurfer's *recon‐all* pipeline. For cortical parcellations, we used the Desikan‐Killiany atlas and for subcortical segmentation, we used the FreeSurfer automated segmentation (aseg). Guided by the heightened saliencies derived from our difference maps and prior AD/LB literature, we focused our analyses on four major anatomical compartments. Specifically, the MTL included the bilateral hippocampus, entorhinal cortex, amygdala, and parahippocampal gyrus; the basal ganglia comprised the bilateral caudate, putamen, accumbens, and pallidum; the occipital lobe encompassed the lateral occipital, cuneus, pericalcarine, and lingual cortices; and the middle temporal region consisted of the mid‐portion of the bilateral temporal cortex. The MTL was treated as the primary AD‐focused ROI family, whereas basal ganglia, occipital, and middle temporal regions were included as secondary ROIs because they emerged in the saliency analyses and prior literature. We also considered fusiform, cingulum, and insula in our supplemental analysis.

### 2.6 Neuropsychological assessments

For the cognitive performance evaluation among the AD/LB pathology groups, we considered a comprehensive set of neuropsychological test scores that were completed within one year of SAA, including measures of global cognition such as Clinical Dementia Rating Sum of Boxes (CDR‐SB) and Alzheimer's Disease Assessment Scale‐Cognitive Subscale (ADAS‐Cog‐11). Cognitive domain scores were also evaluated, reflecting memory,^56^ language, visuospatial function,^57^ and executive function.^58^

### 2.7 Statistical analysis

#### 2.7.1 Demographic and cohort comparisons

We first assessed group‐level demographic differences among the four pathology subgroups (AD−LB−, AD+LB−, AD−LB+, and AD+LB+) using chi‐squared tests for categorical variables and one‐way analysis of variance (ANOVA) for continuous variables. Similarly, before training the BA DL model, we used a one‐way ANOVA and a chi‐square test, respectively, to ensure there were no significant differences in CA and sex distribution across the training, validation, and test subsets of CU individuals. In Supporting Information, we also assessed age‐bin distributions across subsets using chi‐square tests applied to both decade bins and the exact discretized age bins used for stratified sampling.

#### 2.7.2 BA model performance assessment and bias correction

To assess the accuracy of our BA estimations from the MRI scans, we computed the MAE between the predicted BA and CA, along with Spearman's correlation (r), and the coefficient of determination ($R^{2}$). The Spearman's correlation coefficient evaluated the monotonic relationship between BA and CA, while $R^{2}$ reflected the proportion of the variance in BA explained by CA in a linear model. We additionally assessed calibration of predicted versus CA by estimating the slope and intercept from linear regression of predicted age on CA. These performance and calibration metrics were calculated for the pooled CU test set and, to make cross‐cohort generalization explicit, separately within each source cohort in the held‐out CU test set (details are provided in the Supporting Information, Section S2).

The BAG—the difference between a DL–predicted BA based on the T1‐weighted MRI scan and a participant's CA (BA–CA)—is used to assess the neurodegenerative burden. Since BAG is derived from a normative structural age prediction model, in neurodegenerative disease a positive BAG is interpreted primarily as pathology‑related neurodegenerative burden (deviation from normative structural aging), rather than direct evidence of accelerated biological aging mechanisms. To account for the well‐known bias in BA predictions, where estimates are often correlated with CA—resulting in overestimation in younger individuals and underestimation in older individuals due to regression dilution^59^, ^60^—and can exhibit sex‐linked offsets, we implemented a linear age‐ and sex‐bias correction.^61^ Specifically, we fitted a linear model to the validation data of the CU group relating the model's raw predicted BA to CA and sex (dummy‐coded as 0 for female, 1 for male): $\hat{\mathrm{BA}}=\beta_{0}+\beta_{\mathrm{age}}\cdot \mathrm{CA}+\beta_{\mathrm{sex}}\cdot Sex$, and computed the age‑and‑sex bias‐corrected BA as:

$$\;{\hat{\mathrm{BA}}}^{\mathrm{corr}}=\frac{\hat{\mathrm{BA}}-\beta_{0}-\;\beta_{\mathrm{sex}}\cdot \mathrm{Sex}}{\;\beta_{\mathrm{age}}}$$

With corrected BAG defined as ${\hat{\mathrm{BA}}}^{\mathrm{corr}}-\mathrm{CA}$. The coefficients were derived once from the CU validation set and then applied consistently to the CU test set, the CU longitudinal subset from ADNI, and all pathology subgroups for bias correction. This validation‐only procedure avoided information leakage and minimized the possibility that between‐group chronological‐age differences could artifactually influence BAG. All reported BAG analyses use this corrected BAG. The same bias‐correction and primary BAG analyses were repeated for the reduced‐capacity DenseNet variants as a sensitivity analysis for model capacity (details are provided in the Supporting Information, Section S5).

#### 2.7.3 BAG comparison among groups

Corrected BAGs were compared among groups using generalized estimating equations (GEEs) fit at the scan level, which accounted for repeated scans within participants by clustering on participant ID. All models used an exchangeable working correlation and robust (sandwich) standard errors. To ensure equal total contribution per participant despite differing numbers of scans, each scan was weighted by 1/*n_i_* (*n_i_ * = number of scans for participant *i*), so that weights summed to 1 within participant. The primary analysis evaluated the group differences and was adjusted for age, sex, cognitive status, apolipoprotein E (APOE) ‐ε4 genotype status, years of education, and recruitment site.

#### 2.7.4 Sex‐stratified BAG analyses

Next, we investigated sex effects in two complementary ways: first, by performing stratified analyses to assess group differences separately within males and females; and second, by fitting a separate model within each group to directly estimate the effect of sex while adjusting for the same covariates. All pairwise comparisons were derived from Wald contrasts and corrected for multiple testing using the Holm–Šídák correction.

#### 2.7.5 Baseline and longitudinal BAG and regional volumetric analyses

To investigate group‐specific trajectories of BAG and regional structural atrophy, we used linear mixed‐effects models (LMMs) with subject‐specific random intercepts and random slopes on time, and examined baseline differences using general linear models. For BAG and regional MRI volumes, fixed effects included pathology group, time, and their interaction (group×time) to test group‐dependent rates of change; when appropriate, a quadratic time term and group×time^2^ interaction were added to flexibly capture non‐linear trajectories. Model preference (linear vs. quadratic) was determined by Bayesian Information Criterion (BIC), selecting the lower‐BIC specification to balance fit and parsimony. A significant group×time term was interpreted as evidence of group‐dependent differences in slope, and a significant group×time^2^ term as evidence of group‐dependent acceleration/deceleration. Throughout the manuscript, *baseline BAG* refers to the cross‐sectional BAG at the participant's first included MRI, whereas *longitudinal BAG slope* refers to the annual change in BAG estimated from the mixed‐effects model. The statistical models are provided in the Supporting Information. Across all models (both cross‐sectional and longitudinal), adjustments included baseline age, baseline cognitive status, sex, APOE‐ε4 status, years of education, and recruitment site.

For models involving gray matter volumes, intracranial volume (ICV) was additionally included as a covariate to adjust for between‐subject differences in head size. We chose this approach rather than simple proportional scaling of regional volumes by ICV (i.e., ROI/ICV) because regional brain volumes do not generally scale strictly proportionally with ICV, and many ROIs show region‐specific, approximately allometric relationships with head size. Under these conditions, ratio normalization can over‐ or under‐correct individual regions and may leave residual or reversed associations with ICV. Including ICV directly in the regression/mixed‐effects models provided flexible head‐size adjustment within the same inferential framework used to test group, sex, and longitudinal effects, while retaining regional volumes in their native units. Pairwise group contrasts were derived from Wald tests and adjusted for multiple comparisons using FDR. For the key BAG and regional volumetric contrasts emphasized in the study, we additionally report effect sizes and 95% confidence intervals in the Supporting Information, using Hedges'g for baseline comparisons and standardized β for longitudinal models, alongside the corresponding raw adjusted differences (years or cm^3^; years/year or cm^3^/year), because standardized β values for annualized group×time terms can be numerically small even when the accumulated between‐group divergence over follow‐up is biologically meaningful. As a sensitivity analysis, we repeated the primary BAG models after excluding the AD−LB+ subgroup and reported the results in the Supporting Information, Section S8.

#### 2.7.6 BAG incremental value analyses

To assess whether BAG provides incremental value beyond conventional MRI summaries, we compared baseline and longitudinal cognitive models including BAG, a whole‐brain normative atrophy summary, and conventional volumetric markers (e.g., global cortical gray matter volume), as detailed in Supporting Information, Section S15. ΔBIC and likelihood‑ratio test (LRT) *p*‑values were used as formal evidence.

#### 2.7.7 Mediation analyses

We also tested whether BAG mediates the association between LB positivity and cognition within the AD+ stratum. Two prespecified designs were used. First, a baseline (cross‑sectional) mediation model estimated the natural indirect effect using the product‑of‑coefficients approach with linear regression models for the mediator and outcome models. Second, a baseline‑to‑change mediation model evaluated whether baseline BAG accounted for subsequent cognitive decline: participant‑level slopes (units per year) were obtained from linear mixed‑effects models with random intercepts and slopes, and these slopes were used as outcomes in otherwise identical mediation models. The indirect effect (average causal mediation effect [ACME]; *a*·*b*), direct effect (average direct effect [ADE]; *c*′), and total effect (TE; *c*), alongside the proportion mediated (PM = ACME/TE), are reported in the results section and Supporting Information. Uncertainty was quantified with bias‑corrected and accelerated (BCa) bootstrap 95% confidence intervals from 5000 resamples; *p*‑values are two‑sided, and multiplicity was controlled within each analysis family with FDR. Including an exposure‐mediator interaction term in the outcome model for both designs did not materially change the ACME or statistical conclusions. All models were adjusted for age, sex, education, and APOE‐ε4 status. Cognitive diagnosis was not included as a covariate to avoid over‐adjustment for a post‐exposure variable derived from cognitive assessments similar to the outcome measures, which could bias the indirect effect in mediation analyses.^62^ Identification assumes sequential ignorability (no unmeasured confounding of exposure to mediator and mediator to outcome, given covariates), no mediator‐outcome confounders affected by exposure, and correct model specification (linearity and random‐effects structure). Mediation estimates are interpreted cautiously; statistical significance alone does not establish causation. All statistical analyses were conducted in Python with Statsmodels/SciPy. Statistical significance was set at two‐sided (*p* < 0.05). Unless otherwise specified, all reported *p*‐values are adjusted for multiple comparisons.

## 3 RESULTS

### 3.1 Cohort characteristics

The CU dataset was drawn from five cohorts—NACC, ADNI, AIBL, CamCAN, and HCP‐A—and restricted to one scan per participant to minimize data leakage and the risk of overfitting. We first combined these data (*n* = 4355; 65.9 ± 13.3 years, 63.2% female) and as described in Section 2, partitioned them into training, validation, and test sets, carefully stratifying by CA. A one‐way ANOVA showed no significant difference in age distributions among these subsets (*F* = 0.461, *p* > 0.05), and a chi‐squared test confirmed no significant difference in sex ($\chi^{2}$ = 1.29, *p* > 0.05). Table S1 summarizes the overall characteristics and partitioning of the CU dataset, whereas Table S2 and Figure S1 show the age distribution of the actual training cohort and the preserved age‐bin composition across the training, validation, and test subsets used for model development.

The 803 CI individuals were stratified by AD pathology and LB pathology into four subgroups: AD+LB+ (*n* = 166), AD+LB− (*n* = 396), AD−LB+ (*n* = 46), and AD−LB− (*n* = 195). Baseline demographic, cognitive, and volumetric characteristics are summarized in Table 1. Formal omnibus comparisons showed an overall group difference in age (*F* (3, 799) = 3.21, *p* < 0.05), but not in sex or education. Nevertheless, the proportion of female participants was numerically lowest in the AD−LB+ subgroup (28.3% vs. 39.2%–42.4% in the other subgroups). Cognitive status and APOE‐ε4 distribution differed significantly across groups (*χ* ^2^(3) = 95.37 and *χ* ^2^(6) = 159.87, both *p* < 0.001). All baseline cognitive measures also differed across groups (all *p* < 0.001), with the greatest impairment observed in the AD+ subgroups, particularly AD+LB+. Baseline regional volumetric measures likewise showed significant overall group differences (all *p* < 0.05). The AD−LB+ subgroup was substantially smaller than the other subgroups and included very few dementia cases, likely reducing statistical power and limiting the precision of estimates and contrasts involving this subgroup. Accordingly, findings involving AD−LB+, particularly null or weaker effects, should be interpreted cautiously rather than as evidence of no difference.

**TABLE 1:** Baseline demographic, cognitive, and volumetric characteristics of cognitively impaired individuals by AD/LB biomarker status.

Parameter	AD−LB− (*n *= 195)	AD−LB+ (*n *= 46)	AD+LB− (*n *= 396)	AD+LB+ (*n* = 166)	Statistic	*p*‐value
**Demographics**						
Age (year)	72.0 ± 8.2	73.8 ± 7.2	73.5 ± 7.0	74.3 ± 7.2	*F*(3, 799) = 3.21	<0.05
Sex, F (%)	79 (40.5%)	13 (28.3%)	168 (42.4%)	65 (39.2%)	*χ* ^2^(3) = 3.59	>0.05
Education (year)	16.2 ± 2.6	16.8 ± 2.4	15.9 ± 2.7	15.9 ± 3.0	*F*(3, 799) = 1.81	>0.05
**Cognitive status**					*χ* ^2^(3) = 95.37	<0.001
MCI	177 (90.8%)	40 (87.0%)	235 (59.3%)	79 (47.6%)		
Dementia	18 (9.2%)	6 (13.0%)	161 (40.7%)	87 (52.4%)		
**APOE‐ε4**					*χ* ^2^(6) = 159.87	<0.001
Non‐carrier	152 (78.0%)	40 (87.0%)	135 (34.1%)	49 (29.5%)		
Heterozygous, 1	42 (21.5%)	3 (6.5%)	196 (49.5%)	81 (48.8%)		
Homozygous, 2	1 (0.5%)	3 (6.5%)	65 (16.4%)	36 (21.7%)		
**Cognition**						
MMSE	28.12 ± 1.97	27.74 ± 1.87	26.48 ± 2.64	25.58 ± 2.74	*F*(3, 799) = 36.27	<0.001
CDR‐SB	1.44 ± 1.28	1.29 ± 0.95	2.28 ± 1.81	2.67 ± 1.88	*F*(3, 799) = 21.64	<0.001
ADAS11	8.49 ± 4.65	9.08 ± 4.18	12.83 ± 6.44	15.45 ± 7.45	*F*(3, 798) = 43.92	<0.001
***Missing*** *	0	0	0	1		
Memory	0.56 ± 0.97	0.44 ± 0.75	−0.34 ± 0.97	−0.74 ± 0.97	*F*(3, 799) = 67.12	<0.001
Language	0.38 ± 0.91	0.32 ± 0.76	−0.18 ± 1.04	−0.57 ± 1.05	*F*(3, 799) = 31.19	<0.001
Visuospatial	0.12 ± 0.68	0.19 ± 0.57	−0.23 ± 0.89	−0.48 ± 0.96	*F*(3, 799) = 18.55	<0.001
Executive function	0.4 ± 0.85	0.066 ± 0.79	−0.34 ± 1.05	−0.83 ± 1.08	*F*(3, 799) = 48.86	<0.001
FAQ	2.95 ± 5.51	4.33 ± 5.63	7.74 ± 7.68	9.96 ± 8.38	*F*(3, 792) = 32.33	<0.001
***Missing*** *	3	0	3	1		
mPACCtrailsB	−3.69 ± 4.77	−4.66 ± 3.97	−9.49 ± 6.45	−12.12 ± 6.50	*F*(3, 799) = 72.27	<0.001
mPACCdigit	−4.23 ± 5.38	−4.89 ± 4.33	−10.18 ± 6.83	−12.65 ± 6.74	*F*(3, 799) = 65.68	<0.001
**Gray matter volumetrics (cm^3^)**						
Hippocampus	4.847 ± 0.937	4.540 ± 0.889	4.328 ± 0.792	4.089 ± 0.698	*F*(3, 798) = 31.10	<0.001
Parahippocampus	2.503 ± 0.498	2.432 ± 0.464	2.341 ± 0.435	2.227 ± 0.429	*F*(3, 797) = 12.36	<0.001
Entorhinal	2.334 ± 0.613	2.227 ± 0.518	2.106 ± 0.546	1.919 ± 0.523	*F*(3, 798) = 17.31	<0.001
Amygdala	1.997 ± 0.439	1.869 ± 0.427	1.712 ± 0.405	1.548 ± 0.363	*F*(3, 798) = 40.30	<0.001
Occipital lobe	28.397 ± 3.623	27.304 ± 4.063	26.913 ± 3.663	26.279 ± 3.330	*F*(3, 798) = 11.11	<0.001
Basal ganglia	12.647 ± 1.658	12.238 ± 1.517	12.323 ± 1.555	12.028 ± 1.399	*F*(3, 798) = 3.65	<0.05
Middle temporal	13.421 ± 1.849	12.744 ± 2.081	12.257 ± 2.063	11.688 ± 1.923	*F*(3, 798) = 25.43	<0.001
Cingulum	11.725 ± 1.377	11.386 ± 1.399	11.184 ± 1.441	10.884 ± 1.281	*F*(3, 798) = 11.16	<0.001
Insula	8.605 ± 0.961	8.417 ± 1.043	8.402 ± 0.966	8.151 ± 0.938	*F*(3, 798) = 5.58	<0.001
Fusiform	11.4 ± 1.459	11.279 ± 1.697	10.622 ± 1.623	10.366 ± 1.612	*F*(3, 798) = 17.18	<0.001

*Note*: Participants were drawn from the ADNI cohort and classified into four subgroups according to their corresponding CSF biomarker results: AD−LB− (*n* = 195), AD−LB+ (*n* = 46), AD+LB−(*n* = 396), and AD+LB+ (*n* = 166). Values are presented as mean ± SD or counts (%). Cognitive measures include MMSE, CDR‐SB, ADAS11, domain‐specific composites (Memory, Language, Visuospatial, Executive), Functional Activities Questionnaire (FAQ), and mPACC metrics (mPACCdigit, mPACCtrailsB). Volumetric measures (normalized to intracranial volume) cover medial temporal, basal ganglia, occipital, and related regions often affected by AD and/or LB pathology. These baseline data show that participants with co‐pathology (AD+LB+) tend to have more cognitive impairments and smaller volumetric measures. Formal group comparison statistics are reported in the Statistic and *p*‐value columns. Categorical variables were compared using *χ* ^2^ tests; continuous variables using one‐way ANOVA. Missing values are shown where applicable.

Abbreviations: AD, Alzheimer's Disease; ADAS11, Alzheimer's Disease Assessment Scale‐Cognitive Subscale (11‐item version); ADNI, Alzheimer's Disease Neuroimaging Initiative; CDR‐SB, Clinical Dementia Rating Sum of Boxes; CSF, cerebrospinal fluid; F, female; FAQ, Functional Activities Questionnaire; LB, Lewy body; MCI, mild cognitive impairment; MMSE, Mini‐Mental State Examination; mPACC, modified Preclinical Alzheimer's Cognitive Composite; SD, standard deviation.

### 3.2 BA estimation in CU individuals

**FIGURE 2:**
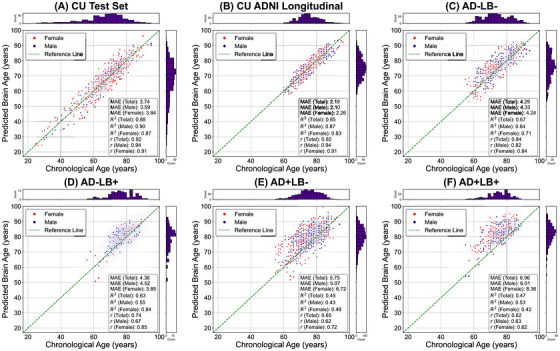
Model performance in predicting brain age across cognitively unimpaired and cognitively impaired AD/LB subgroups. Scatter plots compare predicted brain age (y‐axis) and chronological age (x‐axis) for (A) cognitively unimpaired individuals, highlighting the combined multicohort test set (CU Test Set), (B) a separate longitudinal subset from ADNI (CU ADNI Longitudinal; from individuals not included in the training phase to avoid leakage), (C–F) each AD/LB pathological subgroup. Each datapoint represents a scan, colored by sex (red dots for females, blue dots for males). The diagonal dashed line shows a perfect 1:1 correspondence between predicted and true ages. Histograms along the axes depict the distribution of chronological ages and predicted brain ages for each subset. Mean absolute error (MAE), coefficient of determination (*R* ^2^), and Spearman's correlation (*r*) are reported for each group (and by sex) in the inset boxes, underscoring robust model generalization in CU individuals and elevated brain age gaps in pathological subgroups. Cohort‐stratified held‐out test‐set calibration plots are provided in Figure S2. AD, Alzheimer's disease; ADNI, Alzheimer's Disease Neuroimaging Initiative; CU, cognitively unimpaired; LB, Lewy body.

We trained a 3D‐DenseNet model to estimate BA from T1‐weighted MRI scans of CU individuals, encompassing a broad age range (23–100 years), ensuring robust capture of normative aging trajectories. After training, we evaluated the model on the CU test set (*n*_test_ = 436), which included scans from all five cohorts. To make cohort‐level generalization within the held‐out CU test set explicit, we examined performance separately in ADNI, AIBL, NACC, CamCAN, and HCP‐A. Cohort‐stratified held‐out test‐set performance remained favorable across cohorts, with MAEs ranging from 2.47 to 4.60 years, Spearman's *r* from 0.83 to 0.96, and calibration slopes from 0.93 to 1.01 (Table S3; Figure S2). The model's ability to generalize across site‐ and population‐level differences was therefore confirmed on this diverse test set and further validated through a rigorous LOCO cross‐validation (see Table S4). The age‐ and sex‐ bias corrected BAG averaged near zero (0.16 ± 0.22 years, MAE = 3.74 ± 0.13 years), with strong agreement (*R* ^2^ = 0.88) between predicted and CAs (Figure 2A). We compared our model's predictive performance against the widely adopted brainageR, using the same CU test splits across all five cohorts. Our model achieved lower MAE than brainageR in all cohorts not included in the brainageR training set (see Table S5). Reduced‐capacity 3D‐DenseNet sensitivity models (approximately 111 and 60.5 M parameters) showed higher CU test prediction error (MAE 4.39 and 4.54 years, respectively), indicating reduced normative sensitivity relative to the primary model (Supporting Information Section S5; Tables S6, S7).

Next, we applied the model to the longitudinal scans of the CU individuals in ADNI (excluding those participants used in the model training phase to avoid leakage). This step was aimed to test the model's reliability in capturing normal aging in this cohort and to generate a reference point for subsequent comparisons with ADNI‐derived AD/LB pathology subgroups. After bias correction, the average BAG was 0.12 ± 0.10 years and the MAE was 2.19 ± 0.06 years, suggesting robust performance on 691 scans of the CU data in ADNI (Figure 2B).

### 3.3 BAG comparison across pathology subgroups

**FIGURE 3:**
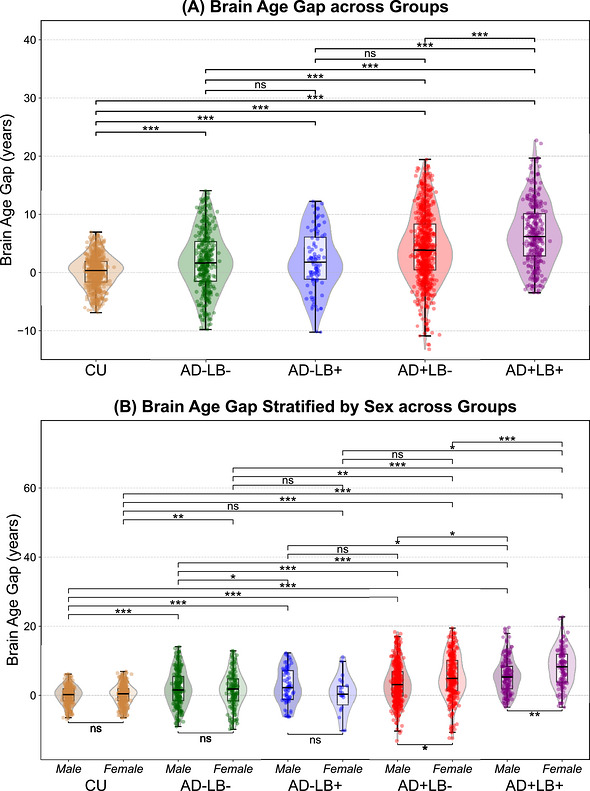
Bias‐corrected brain age gaps across subgroups. (A) Violin plots showing the distribution of age‐ and sex‐ bias‐corrected brain age gaps across the four pathological subgroups (AD−LB−, AD−LB+, AD+LB−, and AD+LB+), as well as the cognitively unimpaired reference (CU). The black box in each violin denotes the interquartile range and median, while whiskers mark the minimum and maximum data points within 1.5 × IQR. Each datapoint represents a scan. Generalized estimating equation (GEE; robust SEs) analyses with Holm–Šídák correction indicate that all pathological subgroups differ significantly from CU (***p < 0.001), with the co‐pathology subgroup (AD+LB+) showing the largest deviation. These results underscore the amplified neurodegenerative impact when AD and LB processes co‐occur. The model was adjusted for age, sex, cognitive state, APOE‐ε4 genotype status, years of education, and recruitment site. (B) Age‐ and sex‐ bias‐corrected brain age gaps stratified by sex within all subgroups. Violin plots display the distribution of age gap estimates, with box plots indicating the median and interquartile ranges. Each datapoint represents a scan. GEE models (with robust SEs and Holm–Šídák corrected) reveal that within the AD+ subgroups (AD+LB−, AD+LB+), females exhibit significantly higher brain age gaps. This pattern underscores potential sex‐specific susceptibility once AD pathology is present. Stratified GEE models for males and females separately confirm that co‐pathology (AD+LB+) yields the largest deviation in both sexes. Models were adjusted for age, cognitive state, APOE‐ε4 genotype status, years of education, and recruitment site. (ns: not significant; *p < 0.05; **p < 0.01; ***p < 0.001). AD, Alzheimer's disease; ANOVA, analysis of variance; APOE, apolipoprotein E; IQR, interquartile range; LB, Lewy body; SE, standard error.

Deploying the model trained with normative cohorts, we estimated BA in the four CI AD/LB pathology subgroups. Figure 3 shows the distribution of these age‐ and sex‐ bias corrected BAGs, and corresponding summary results are presented in Table S8. All AD/LB pathology subgroups showed significantly elevated BAGs relative to the CU reference—indicating that their structural MRI‐derived BAs exceeded CAs to a greater extent than in CU individuals. Notably, as shown in Figures 2C–F, the co‐pathology subgroup (AD+LB+) exhibited the largest deviation (6.61 ± 0.3 years; MAE = 6.96 ± 0.27), exceeding that of both AD+LB− (4.32 ± 0.21 years; MAE = 5.75 ± 0.16) and AD−LB+ (1.98 ± 0.55 years; MAE = 4.36 ± 0.36), as well as AD−LB− (1.83 ± 0.26 years; MAE = 4.29 ± 0.17).

A GEE analysis, with robust SEs, adjusted for age, sex, cognitive state, APOE‐ε4 status, years of education, and recruitment site, indicated a highly significant overall group effect on BAGs (*χ* ^2^(4) = 156.07, *p* < 10^−^^32^). Post hoc Holm–Šídák Wald contrasts further indicated that each pathology subgroup differed significantly from the CU reference (all *p* < 10^−^^6^), confirming markedly elevated BAGs across all AD/LB subgroups. As depicted in Figure 3A, among the CI pathology subgroups themselves, the only non‐significant pairs were between AD−LB− versus AD−LB+ and AD−LB+ versus AD+LB−. Further subgroup comparisons revealed that the co‐pathology condition (AD+LB+) surpassed both AD or LB pathology alone, underscoring a potential compounding effect on neurodegenerative burden when AD and LB co‐occur.

### 3.4 Sex differences in BAGs

To investigate the influence of sex on BAGs across subgroups, we conducted two complementary analyses. First, we performed pairwise comparisons of males versus females within each subgroup. Second, we assessed differences among subgroups for each sex separately. These models were adjusted for the same set of covariates as above, except for sex itself. Figure 3B displays the distribution of the age‐ and sex‐bias‐corrected BAGs for males and females in each subgroup.

From the within‐group comparisons, in the CU reference and AD− subgroups, male–female differences were not significant. In contrast, within the AD+ subgroups (AD+LB− and AD+LB+), females exhibited significantly higher BAGs than males (AD+LB−: *p* < 0.05, AD+LB+: *p* < 0.01). This pattern indicates a sex‐specific susceptibility once AD pathology is present.

To formally test whether this sex gap was exacerbated by LB co‐pathology, we modeled the group and sex interaction and evaluated the contrast of female‐male BAG differences (AD+LB+ vs. AD+LB−). The analysis revealed that the female‐specific elevation in BAG was significantly more pronounced in the co‐pathology subgroup. The difference in BAG between females and males was 1.97 years greater in AD+LB+ compared to AD+LB− (i.e., BAG[F–M] _[AD+LB+]_—BAG[F–M] _[AD+LB−]_ = 1.97 years; *p* < 0.05). This suggests not only that females exhibit a heightened vulnerability once AD pathology is present, but that this vulnerability is significantly amplified by concomitant LB pathology. When pooling all males and all females separately by subgroup, sex‐specific GEE models indicated strong group effects for both sexes (males: *χ* ^2^(4) = 62.58, *p* < 10^−^^12^; females: *χ* ^2^(4) = 89.41, *p* < 10^−^^17^). The AD+LB+ subgroup had significantly elevated BAGs relative to all other subgroups in both sexes, mirroring the pattern seen in the combined analysis.

### 3.5 Baseline and longitudinal comparisons of BAG across groups

To further elucidate how LB co‐pathology shapes both cross‐sectional structural burden and subsequent change, we evaluated baseline BAG and longitudinal BAG slope, respectively. Specifically, we fitted linear mixed‐effects models (random intercepts and slopes) to the 1,574 scans across all subgroups, modeling BAG as a function of time since baseline and its interaction with group. Baseline comparisons were analyzed via a general linear model. All models were adjusted for baseline age, sex, baseline cognitive state, APOE‐ε4 status, years of education, and recruitment site, with CU ADNI longitudinal serving as the reference group. Model comparisons via BIC favored a linear model over a quadratic one.

**FIGURE 4:**
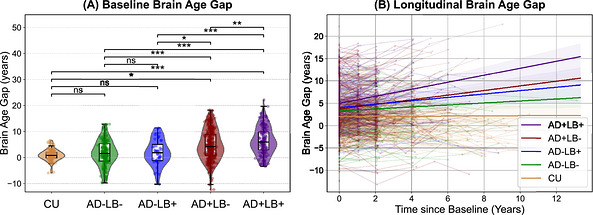
Baseline and longitudinal comparisons of brain age gap across the cognitively unimpaired reference group and the pathology subgroups. Violin plots in (A) illustrate the distribution of brain age gaps at baseline across the four AD/LB subgroups, as well as the cognitively unimpaired reference (CU), with overlaid boxplots indicating median and interquartile ranges. Pairwise group comparisons were conducted using a general linear model (ns: not significant; *p < 0.05; **p < 0.01; ***p < 0.001). (B) Thin lines represent individual changes in brain age gap over time (shown as spaghetti plots only for visual context), while thick lines with shaded bands indicate population‐level trajectories predicted from the fitted linear mixed‐effects model (random intercepts and slopes), with corresponding 95% confidence intervals. The AD+LB+ subgroup exhibits the steepest slope, reflecting a significantly faster divergence from CU (Δ*β* = +0.77 years/year; *p* < 0.001), AD−LB− (Δ*β* = +0.56 years/year; *p* < 0.001), AD−LB+ (Δ*β* = +0.41 years/year; *p* < 0.05), and AD+LB− (Δ*β* = +0.27 years/year; *p* < 0.05), highlighting the additional neurodegenerative burden imposed by LB co‐pathology in the presence of AD. Both cross‐sectional and longitudinal models are adjusted for baseline age, sex, baseline cognitive status, APOE‐ε4 status, years of education, and recruitment site, with all results corrected for multiple comparisons. AD, Alzheimer's disease; LB, Lewy body.

At baseline, relative to CU, both AD+LB− (Δ*β* = +2.32 years, SE = 0.86, *p* < 0.05) and AD+LB+ (Δ*β* = +3.57 years, SE = 0.91, *p* < 0.001) displayed significantly higher BAGs, whereas AD−LB− and AD−LB+ did not differ from CU. Pairwise contrasts among patient subgroups further indicated that AD+LB+ also exceeded both AD−LB+ (Δ*β* = +2.95 years, SE = 0.83, *p* < 0.001) and AD+LB− (Δ*β* = +1.25 years, SE = 0.44, *p* < 0.01); and AD+LB− exceeded AD−LB+ (Δ*β* = +1.70 years, SE = 0.77, *p* < 0.05) at baseline (Figure 4A).

Turning to slopes (i.e., the yearly change in BAG), relative to the reference CU, which had a negligible yearly slope (*β* = 0.02), all disease groups showed faster increases over time: AD−LB− (Δ*β* = +0.20 years/year, SE = 0.10, *p* < 0.05), AD−LB+ (Δ*β* = +0.36 years/year, SE = 0.16, *p* < 0.05), AD+LB− (Δ*β* = +0.50 years/year, SE = 0.09, *p* < 0.001), and AD+LB+ (Δ*β* = +0.77 years/year, SE = 0.12, *p* < 0.001). Pairwise contrasts confirmed that AD+LB+ outpaced AD+LB− (Δ*β* = +0.27 years/year, SE = 0.10, *p* < 0.05) and AD−LB+ (Δ*β* = +0.41 years/year, SE = 0.17, *p* < 0.05), and it also exceeded AD−LB− (Δ*β* = +0.56 years/year, SE = 0.11, *p* < 0.001), underscoring the additional neurodegenerative burden imposed by co‐occurring AD and LB pathologies (Figure 4B). AD+LB− progressed faster than AD−LB− (Δ*β* = +0.30 years/year, SE = 0.06, *p* < 0.001), whereas its difference from AD−LB+ did not reach significance; like the comparison between AD−LB+ and AD−LB−. Overall, these findings indicate that although AD positivity alone is associated with higher BAG and faster increases in BAG over time (i.e., faster divergence from normative structural aging patterns), the concomitant presence of LB pathology amplifies this divergence even further, leading to a more pronounced and rapidly evolving deviation from normative aging trajectories. (AD+LB+ > AD+LB− > AD−LB+ ≈ AD−LB− > CU). Effect‐size estimates supported the same pattern, with wider confidence intervals for contrasts involving the smaller AD−LB+ subgroup (Tables S9, S10). To test whether the small AD−LB+ subgroup drove the primary BAG findings, we repeated the main BAG analyses after excluding AD−LB+ entirely. The pattern was unchanged: AD+LB+ remained significantly higher than AD+LB− in the all‐scan BAG model, the baseline BAG model, and the longitudinal BAG‐slope model (Table S11).

To assess robustness to site effects, we repeated the key analyses after harmonization using ComBat‐GAM (age modeled as a smooth term). The pattern of results was unchanged: AD+LB+ consistently showed the largest deviation from normative ageing and the steepest longitudinal increase, followed by AD+LB−, with limited effects in AD−LB+. Paired equivalence tests (Two One‐Sided T‐tests [TOST], ±1 year) indicated that predicted BAs remained statistically equivalent before versus after harmonization. Details are provided in Supporting Information, Section S9. Taken together, the site‐harmonization results complement the held‐out test, cohort‐stratified held‐out evaluation, LOCO validation, and brainageR benchmarking supporting that the main findings are not driven by cohort‐specific artifacts. The primary cross‐sectional and baseline BAG findings were robust to reduced model capacity: in both shallower DenseNet variants, the omnibus group effect remained highly significant and the AD+LB+ versus AD+LB− contrast remained significant in the all‐scan and baseline analyses. In longitudinal analyses, the medium‐capacity model preserved the AD+LB+ versus AD+LB− slope difference, whereas the smallest model retained the same directional ordering but appeared to have insufficient sensitivity to detect this more nuanced longitudinal contrast. Details are provided in Supporting Information, Section S5.

### 3.6 Saliency‐based interpretability analyses

**FIGURE 5:**
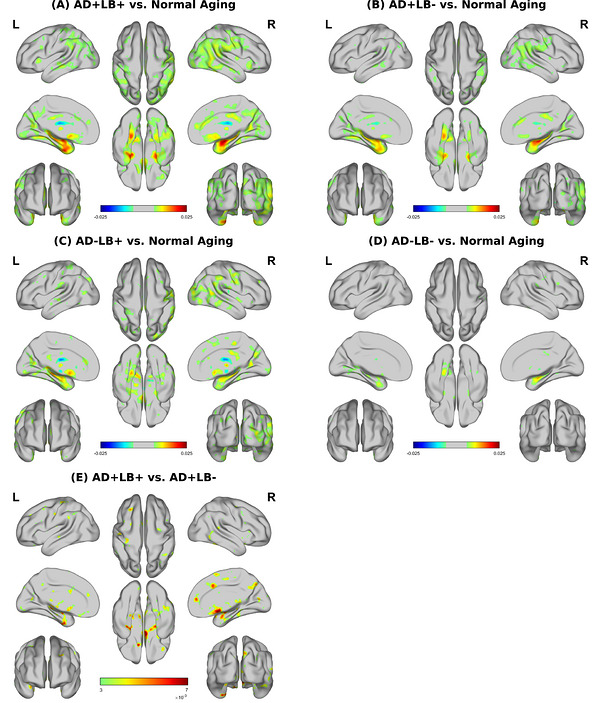
Group‐level difference saliency maps, driven from the deep learning model, highlighting regions contributing to the elevated brain age gap in AD/LB subgroups. (A–D) The difference between the averaged saliency for each AD/LB subgroup versus the cognitively unimpaired (CU) reference; warmer colors (yellow–red) indicate stronger contributions to the model's higher brain age predictions in the pathological subgroup. Conversely, cooler colors (blue–cyan) mark areas in which saliency is greater for the cognitively unimpaired cohort. The AD+LB+ subgroup demonstrates broader and more pronounced saliency involvement than AD+LB− or AD−LB+. (E) The direct comparison of AD+LB+ versus AD+LB−, isolating the additional effect of LB on an AD background. Prominent saliencies involve cholinergic basal forebrain, and parts of medial temporal structures, right cingulum, right precuneus, and left fusiform gyrus, suggesting these regions contribute more strongly to the model's higher brain age predictions in co‑pathology. These saliency maps indicate model sensitivity rather than atrophy itself, and the key patterns were replicated with SmoothGrad to ensure their robustness (Figure S1). AD, Alzheimer's disease; LB, Lewy body.

To gain insight into the anatomical regions most relevant to our BA predictions from the MRI scans, we generated gradient saliency maps for each individual and averaged them over each pathology subgroup. These were then compared to the averaged saliency map derived from CU individuals—representative of normative aging. This approach aimed to account for normal aging effects and highlight anatomical features to which the model was most sensitive when estimating BA across subgroups. Because gradient saliency quantifies voxel‐wise influence on age prediction, these maps were interpreted as hypothesis‐generating model‐attribution patterns rather than direct evidence of regional tissue loss or disease‐specific vulnerability. As illustrated in Figure 5A–D, these difference maps revealed that the co‐pathology subgroup (AD+LB+) had comparatively heightened saliency across the medial surfaces, including the MTL and related subcortical structures. Specifically, the AD+ subgroups showed heightened saliency in the amygdala, hippocampus, fusiform gyrus, and entorhinal cortex, indicating a greater contribution of these regions to the model's prediction of older‐appearing BA estimates, with the AD+LB+ group demonstrating the most widespread involvement.

We further observed slightly elevated saliency in parts of the right cingulum, cuneus, precuneus, and lingual for AD+LB+ relative to AD+LB−. Additionally, the LB+ subgroups showed relatively greater saliency in the occipital cortex, middle temporal (posterior aspect), and calcarine cortex compared to LB− subgroups, along with elevated involvement of the thalamus, caudate, and olfactory areas. Pairwise comparisons of mean saliency values for all AD/LB groups versus CU using the Automated Anatomical Labeling (AAL) atlas are provided in Tables S12–S15. To ensure that our interpretability results were robust and not driven by reliance on a single attribution method, we replicated the analysis using SmoothGrad—a gradient‐based enhancement that mitigates noise by averaging saliency maps over multiple perturbed inputs—which yielded a consistent neuroanatomical pattern (Figure S3A–D). Group‑mean and difference maps were compared between the two saliency methods at both the ROI and voxel levels. Across all group comparisons, the two approaches identified highly concordant anatomical patterns of importance for BA prediction, demonstrating strong consistency (Table S16).

Moreover, we compared the averaged saliency map of AD+LB+ directly against that of AD+LB− to isolate the additional saliency signal associated with LB positivity on an existing AD background. As shown in Figure 5E (and Figures S3E, S4), a pronounced positive saliency cluster appeared near the cholinergic basal forebrain, including the NBM, as well as in parts of the MTL, right cingulum, right precuneus, and left fusiform gyrus, indicating that these regions contributed more strongly to the model's predictions in AD+LB+ than in AD+LB−.

### 3.7 Longitudinal effect of co‐pathology on region‐specific atrophy

**FIGURE 6:**
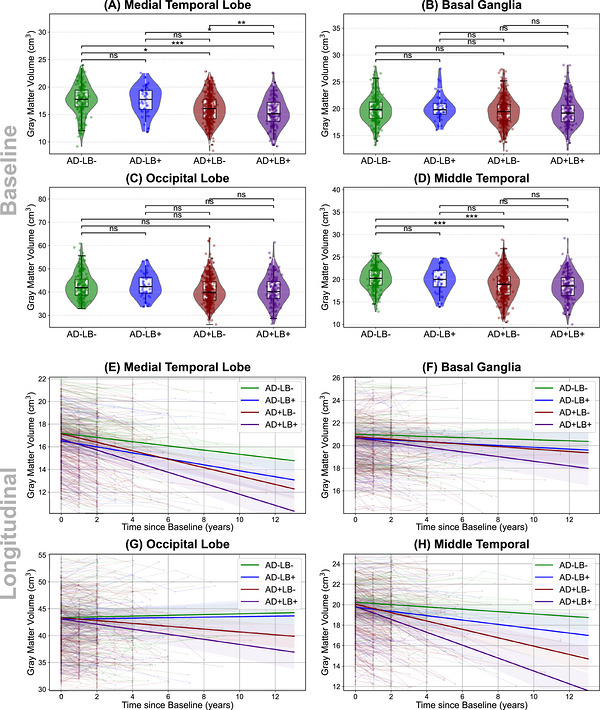
Baseline and longitudinal comparisons of gray matter volume in key AD and LB‐related regions across AD/LB pathology subgroups. (A–D) The distribution of gray matter volume at baseline for the (A) medial temporal lobe, (B) basal ganglia, (C) occipital lobe, and (D) middle temporal cortex across four AD/LB pathological subgroups. Overlaid boxplots indicate medians and interquartile ranges. The top bars and corresponding significance labels reflect pairwise group comparisons under a general linear model (ns: not significant; *p < 0.05; **p < 0.01; ***p < 0.001). (E–H) The longitudinal trajectories of gray matter volume over time for the same four regions. Individual trajectories are shown as thin lines (spaghetti plots; only for visual context), while thick lines with shaded bands indicate population‑level trajectories predicted from the fitted linear mixed‑effects models (random intercepts and slopes), with corresponding 95% confidence intervals. Relative to the single‐pathology subgroups (AD−LB+ or AD+LB−) and AD−LB− reference, the co‐pathology subgroup (AD+LB+) consistently demonstrates the fastest volumetric declines. For instance, in the MTL, AD+LB+ exhibits significantly steeper atrophy than AD−LB− (*p* < 0.001) and AD+LB− (*p* < 0.001); in the occipital lobe, AD+LB+ exceeds AD−LB− (*p* < 0.001) and AD+LB− (*p* < 0.01); in the basal ganglia, AD+LB+ differs from AD−LB− and AD+LB− (both *p* < 0.05); and in the middle temporal cortex, AD+LB+ surpasses AD−LB−, AD−LB+ and AD+LB− (all *p* < 0.001), indicating a more pronounced atrophy pattern under LB co‐pathology. All cross‐sectional and longitudinal models are adjusted for baseline age, sex, baseline cognitive status, APOE‐ε4 status, years of education, recruitment site, and ICV, with all results corrected for multiple comparisons. AD, Alzheimer's disease; APOE, apolipoprotein E; ICV, intracranial volume; LB, Lewy body.

Building on our BAG findings, and using the saliency maps as a hypothesis‑generating guide for ROI selection, we performed a more granular investigation into specific neuroanatomical regions. To clarify how AD/LB proteinopathies combine to drive atrophy beyond what either pathology might incur alone, we focused on the gray matter volume of the MTL (a composite of the hippocampus, amygdala, entorhinal cortex, and parahippocampal gyrus) and key extra‐MTL regions, including the basal ganglia, occipital lobe, and middle temporal cortex. Figure 6 shows representative baseline (via general linear model) and longitudinal comparisons (via linear mixed‐effects model with random intercepts and slopes) across pathology subgroups, with additional results for the fusiform, cingulum, insula, and MTL sub‐regions provided in Figures S5 and S6. Aligned with our global BA analyses, BIC comparisons consistently supported linear rather than quadratic trajectories in each ROI. All models were adjusted for baseline age, sex, baseline cognitive status, APOE‐ε4 status, years of education, recruitment site, and ICV.

At baseline, in the MTL, both AD+LB− (Δ*β* = −0.58 cm^3^, SE = 0.22, *p* < 0.05) and AD+LB+ (Δ*β* = −1.20 cm^3^, SE = 0.27, *p* < 0.001) showed significantly lower volumes compared to AD−LB−, indicating pronounced baseline atrophy. AD−LB+ did not reach statistical significance. The baseline MTL volume in AD+LB+ was also significantly lower than that of AD+LB− (Δ*β* = −0.62 cm^3^, SE = 0.21, *p* < 0.01), and lower than AD−LB+ (Δ*β* = −0.85 cm^3^, SE = 0.39, *p* < 0.05). In the basal ganglia and occipital lobe, none of the subgroups significantly diverged from AD−LB− or from each other. For the middle temporal cortex, AD+LB+ (Δ*β* = −1.14 cm^3^, SE = 0.30, *p* < 0.001) and AD+LB− (Δ*β* = −1.00 cm^3^, SE = 0.25, *p* < 0.001) fell below AD−LB−, while AD−LB+ did not reach statistical significance. Additionally, pairwise contrasts among AD+LB+, AD+LB−, and AD−LB+ were nonsignificant at baseline.

Over time, compared to AD−LB−, AD+LB− (Δ*β* = −0.19 cm^3^/year, *p* < 0.001) and AD+LB+ (Δ*β* = −0.30 cm^3^/year, *p* < 0.001) exhibited steeper MTL atrophy slopes, with AD+LB+ being the highest (vs. AD+LB−: Δ*β* = −0.12 cm^3^/year, *p* < 0.001), supporting that co‐occurring LB pathology amplifies existing AD‐related neurodegeneration within limbic regions classically vulnerable in AD. In the basal ganglia, AD+LB+ declined faster than AD−LB− (Δ*β* = −0.16 cm^3^/year, *p* < 0.05), whereas AD+LB− did not differ from AD−LB−; pairwise comparisons further showed that AD+LB+ surpassed AD+LB− (Δ*β* = −0.10 cm^3^/year, *p* < 0.05). The occipital lobe showed the most pronounced slope in AD+LB+ (Δ*β* = −0.54 cm^3^/year, *p* < 0.001), with AD+LB− also accelerated (Δ*β* = −0.34, *p* < 0.001). AD−LB+ did not reach the threshold for significance. Pairwise comparisons further confirmed AD+LB+ surpassed AD+LB− (Δ*β* = −0.20 cm^3^/year, *p* < 0.01), reinforcing that concomitant LB pathology intensifies atrophy in visually related cortices beyond what is observed in AD alone. Finally, in the middle temporal cortex, AD+LB+ exceeded AD+LB− (Δ*β* = −0.22 cm^3^/year, *p* < 0.001), whereas AD+LB− itself exhibited steeper slopes compared to AD−LB+ and AD−LB− (Δ*β* = −0.20 cm^3^/year, −0.30 cm^3^/year, respectively, both *p* < 0.001). Corresponding effect‐size estimates for the key baseline and longitudinal regional volumetric contrasts are provided in Supporting Information, Section S7.

Overall, AD+LB+ emerged as the most atrophic subgroup at baseline in the MTL and demonstrated the steepest declines in multiple cortical and subcortical structures. Whereas isolated LB often yields borderline or nonsignificant effects, its combination with AD markedly intensified neurodegeneration beyond single‐pathology levels. Therefore, LB co‐pathology compounds both baseline tissue loss and subsequent atrophy rates, aligning with the BA‐based results in indicating an amplified disruption of normal structure.

### 3.8 Incremental cognitive value of BAG and mediation of LB effects within AD

**FIGURE 7:**
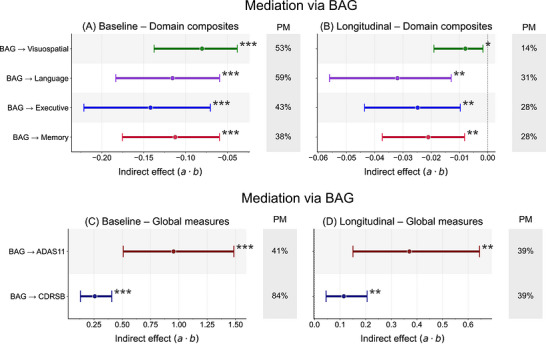
Mediation of the association between Lewy body positivity on an AD background and cognition by brain age gap (BAG). Forest plots display the indirect effect (*a·b*) with bias‐corrected and accelerated (BCa) 95% confidence intervals from 5000 bootstrap resamples. (A, B) Domain composites (visuospatial, language, executive, memory) (A) at baseline and (B) in a baseline‑to‑change design, where the outcome is the participant‑level cognitive slope (units/year) estimated from linear mixed‑effects models with random intercepts and slopes. (C, D) Global measures (ADAS‑Cog 11, CDR‑SB) (C) at baseline and (D) baseline‑to‑change. Sign convention follows clinical interpretation: more negative *a·b* for domain composites indicates poorer performance with higher BAG, whereas more positive *a·b* for global scales indicates worse (higher) scores with higher BAG. Numbers at right report the proportion mediated. Asterisks denote FDR‑adjusted significance (*p < 0.05, **p < 0.01, ***p < 0.001). Analyses were restricted to AD‑positive participants (LB+ vs. LB−) and adjusted for baseline age, sex, APOE‑ε4 status, years of education, and recruitment site. AD, Alzheimer's disease; ADAS11: Alzheimer's Disease Assessment Scale‐Cognitive Subscale 11; CDR‐SB, Clinical Dementia Rating Sum of Boxes; FDR, false discovery rate; LB, Lewy body.

Building on the structural and saliency map findings, analyses of global (CDR‑SB, ADAS‑Cog‑11) and domain‑specific (memory, language, visuospatial, and executive) composites indicated that AD+LB+ performed worse on several measures at baseline and declined more rapidly thereafter than AD+LB− (Figures S7 and S8). To directly evaluate whether BAG captures information beyond established structural MRI summaries, we compared it against both a whole‐brain normative atrophy burden score and conventional volumetric measures of medial temporal and global cortical gray matter atrophy. BAG generally showed better cognitive model fit than these alternative MRI summaries and, importantly, improved model fit when added to models already including conventional volumetrics. Specifically, in baseline cognitive models, BAG was generally preferred to the whole‐brain atrophy summary (ΔBIC for BAG vs. SimpleAtrophy = −11.1 to −4.1; negative values favor BAG). When added to models already including conventional MTL and global cortical gray matter volumetrics, BAG provided additional baseline improvement (ΔBIC add BAG|VOL = −22.9 to −0.59; LRT *p* = 0.004–0.011). In longitudinal analyses, the evidence was stronger and more consistent: BAG improved model fit relative to the whole‐brain atrophy summary (ΔBIC for BAG vs. SimpleAtrophy = −46.3 to −6.6) and adding BAG beyond conventional volumetrics substantially improved fit across all longitudinal cognitive outcomes (ΔBIC add BAG|VOL = −76.4 to −25.7; all LRT *p* < 0.001), supporting that BAG captures clinically relevant neuroanatomical variation related to cognitive decline beyond conventional structural measures (Tables S17A, S17B). Motivated by these findings, we tested whether the neuroanatomical burden captured by BAG mediates the association between LB positivity (within AD+; AD+LB+ vs. AD+LB−) and cognition, evaluating mediation for (i) baseline performance and (ii) subsequent individual‑specific slopes (Figure 7). Mediation models were adjusted for age, sex, APOE‐ε4 status, years of education, and recruitment site.

At baseline, LB positivity on the background of AD (LB+ within AD+) showed statistically significant indirect effects through BAG. Specifically, using BAG as mediator, indirect effects on all four domain composites were negative, indicating LB positivity was associated with greater BAG and, in turn, poorer cognition: memory *a·b* ≈ −0.1123 (bias‐corrected and accelerated [BCa] 95% CI: [−0.1755, −0.0595]; *p* < 0.001; proportion mediated [PM] ≈ 0.38), language ≈ −0.1155 (BCa 95% CI: [−0.1834, −0.0594]; *p* < 0.001; PM ≈ 0.59), visuospatial ≈ −0.0803 (BCa 95% CI: [−0.1376, −0.0383]; *p* < 0.001; PM ≈ 0.53), and executive ≈ −0.1419 (BCa 95% CI: [−0.2214, −0.0704]; *p* < 0.001; PM ≈ 0.43). Coherent positive mediation was observed for global outcomes—ADAS‐Cog‐11 *a·b* ≈ 0.9531 (BCa 95% CI: [0.5086, 1.4854]; *p* < 0.001; PM ≈ 0.41) and CDR‐SB ≈ 0.2575 (BCa 95% CI: [0.1305, 0.4074]; *p* < 0.001; PM ≈ 0.84)—consistent with higher BAG relating to higher (worse) outcomes.

We next investigated whether baseline BAG explains the faster *rate* of cognitive change associated with LB positivity within AD+. In models with mediator measured at baseline (M_bl_) and outcomes specified as individual‐specific slopes (Y_slope_), LB positivity was associated with higher BAG at baseline, and BAG in turn predicted steeper decline on domain composites and greater worsening on global scales. Indirect effects (*a·b*) were significant after FDR correction across all outcomes: memory ≈ −0.0210 (BCa 95% CI: [−0.0373, −0.00818]; *p* < 0.01; PM ≈ 0.28), language ≈ −0.0320 (BCa 95% CI: [−0.0560, −0.0129]; *p* < 0.01; PM ≈ 0.31), executive ≈ −0.0248 (BCa 95% CI: [−0.0436, −0.00971]; *p* < 0.01; PM ≈ 0.28), and visuospatial ≈ −0.0079 (BCa 95% CI: [−0.0190, −0.00170]; *p* < 0.05; PM ≈ 0.14). For global measures, indirect effects were positive—CDR‐SB ≈ 0.115 (BCa 95% CI: [0.045, 0.205]; *p* < 0.01; PM ≈ 0.39) and ADAS‐Cog‐11 ≈ 0.371 (BCa 95% CI: [0.150, 0.644]; *p* < 0.01; PM ≈ 0.39)—indicating that higher baseline BAG portends steeper clinical deterioration. For both designs, the direct (ADE; *c′*), total (TE; *c*), and indirect (ACME; *a·b*) effects, together with the proportion mediated (PM = ACME/TE), are shown in the mediation path diagrams in Figures S9 and S10.

Taken together, BAG partially mediates both the cross‐sectional association between LB positivity (on an AD background) and cognition, and the association with subsequent rates of cognitive change across domain composites and global scales, with directionality consistent across outcomes where higher scores denote worse performance.

## 4 DISCUSSION

Leveraging an interpretable DL model on structural MRI scans from a large, multi‐cohort dataset, and incorporating a comprehensive set of cognitive measures alongside CSF α‐synuclein SAA, we provided converging evidence that individuals harboring concomitant AD and LB pathology (AD+LB+) exhibit faster neurodegeneration, compared to isolated AD or LB. Our findings extend prior reports suggesting that the prevalent co‐occurrence of LB and AD‐related pathologies exerts an amplified effect, driving a significantly greater deviation in BAGs, more pronounced and extensive region‐specific atrophy, and steeper cognitive decline compared to either pathology alone—consistent with MRI‐derived BAG capturing part of the structural pathway by which LB co‐pathology contributes to clinical impairment. Notably, these amplified effects are pronounced across different key regions, including the MTL, occipital lobe, and NBM, suggesting an enhanced vulnerability of circuits typically involved in memory, attention, visuospatial, and executive functioning. This regional profile is broadly consistent with recent multimodal studies of mixed AD/LB disease, which together point to convergent vulnerability of temporolimbic, basal forebrain, and posterior cortical systems.^2^, ^3^, ^6^, ^63^

The trained DL model robustly captured normative brain‐aging trajectories across cohorts. Based on this foundation and aligned with literature demonstrating the utility of BAG as a global marker of neurodegeneration,^26^, ^27^, ^28^, ^32^, ^64^ we assessed the deviations from these normative trajectories in CI individuals with AD/LB pathologies. Significantly elevated baseline BAGs were observed across all pathological subgroups, with the co‐pathology subgroup showing both the highest baseline BAG and the steepest longitudinal BAG increase, exceeding CU individuals and single‐pathology subgroups. Reduced‐capacity sensitivity analyses preserved the main co‐pathology pattern, supporting its robustness. These findings reinforce and extend emerging evidence that LB pathology imposes an additional neurodegenerative burden on top of established AD processes.^2^, ^24^

Sex‐stratified analyses revealed that BAGs were significantly higher in females once AD was present, and that this female‐specific elevation was further amplified in co‐pathology condition, suggesting not only that females have a heightened vulnerability once AD pathology is present but also a significant amplification of that vulnerability with concomitant LB pathology. This is consistent with recent AD biomarker studies showing higher tau burden, stronger amyloid–tau coupling, and faster tau accumulation in females, as well as LB literature indicating that women more often show greater cognitive impairment and more concomitant AD‐related pathology.^65^, ^66^, ^67^, ^68^, ^69^ These findings highlight the necessity of accounting for sex differences in AD/LB studies.

The interpretability afforded by our saliency maps provided a data‐driven view of the anatomical features that most influenced BA prediction in each subgroup, guiding our investigation of corresponding region‐specific atrophies and domain‐specific cognitive decline. We treat saliency as hypothesis‐generating and base atrophy inferences on the volumetric analyses.

Our saliency analyses indicated that AD+LB+ shows an expanded set of salient regions, combining canonical AD targets with those frequently implicated in LB disease. Specifically, these regions contributed most to the elevated BAGs in this subgroup compared to other subgroups. Longitudinal volumetric analyses further demonstrated that the AD+LB+ subgroup experiences steeper volumetric atrophies in these key structures. A particularly striking example of this was observed in the MTL, where AD+LB+ exhibited a steeper decline in volume than AD+LB−, suggesting that α‐synuclein potentially amplifies other AD‐related processes in limbic regions. This faster MTL atrophy paralleled steeper memory decline in AD+LB+ over time, implying a connection between structural degeneration in this critical memory circuit and its clinical manifestation. This is consistent with recent SAA‐anchored tau‐PET findings linking α‐synuclein co‐pathology in AD to greater tau burden in AD‐typical regions, faster amyloid‐driven tau accumulation, and accelerated cognitive decline.^6^ Beyond the MTL, our analyses revealed a more distributed pattern of vulnerability in AD+LB+, encompassing the occipital lobe, the middle temporal cortex, and basal ganglia, consistent with recent fluorodeoxyglucose (FDG)‐PET studies showing greater cortical hypometabolism in AD+LB+ individuals, particularly in posterior cortical regions, with occipital hypometabolism partly dissociating from concurrent AD biomarker levels.^2^, ^70^ These regions also displayed heightened saliency in our DL model, faster atrophy in our longitudinal volumetric analyses, and were paralleled with declines in visuospatial, language, and executive function, respectively.

Prior neuropathology and MRI‐based studies of LB pathology‐associated atrophy have often implicated medial temporal structures, yet these findings have been inconsistent across cohorts.^3^, ^40^, ^41^, ^42^, ^43^ Moreover, while Wisse et al.^3^ highlighted the NBM as a primary atrophic focus in LB‐positive individuals, recent autopsy‐based work shows that posterior basal forebrain atrophy is most severe in mixed AD/LBD, whereas hippocampal atrophy is more specifically associated with AD,^63^ and amygdala‐predominant LB pathology in AD has been associated with cognitive decline and focal amygdala atrophy.^71^ Against this backdrop, our saliency analyses suggested greater model‐attributed importance extending beyond NBM, encompassing parts of MTL, right cingulum/precuneus, and left fusiform gyrus, fit with an emerging view that mixed AD/LB involves both cholinergic basal forebrain and temporolimbic networks.

To probe the mechanism, we tested whether BAG sits on the pathway from LB co‑pathology to cognitive impairment using a prespecified contrast of LB‐positivity within AD. Across outcomes, higher BAG was associated with worse cognition, and BAG accounted for part of the association between LB‐positivity and cognition both at baseline and over time. Effects were evident for global measures and across all domain composites. These patterns support the view that BAG captures a structural substrate through which LB co‑pathology amplifies clinical expression in AD. While mediation in observational data cannot establish causality and BAG reflects global rather than LB‑specific neurodegeneration, these findings suggest that MRI‐derived neurodegenerative burden partly explains the association between LB co‐pathology and cognitive decline.

These interwoven findings illustrate a coherent portrait of the amplified effects of co‐pathology; LB co‐pathology not only broadens the scope of structural decline beyond canonical AD patterns but also exacerbates atrophy in core AD target areas. This multifaceted disruption manifests clinically as more pronounced and wide‐ranging cognitive deficits, a portion of which is statistically explained by the greater BAG.

The BA framework quantifies deviation from normative structural aging, but elevated BAG in neurodegenerative disease should be interpreted as pathology‐related neurodegenerative burden that makes the brain appear *older*, rather than as evidence of accelerated biological aging mechanisms. In this context, BAG may function primarily as a compact nonlinear summary of distributed neurodegenerative burden, not as a distinct biological process independent of established atrophy patterns. This interpretation is supported by our incremental‐value analyses, in which BAG provided information beyond conventional structural MRI measures, most consistently in longitudinal models where spatially distributed structural changes accumulate over time and may be integrated nonlinearly by the model, underscoring its value as a longitudinal biomarker rather than a surrogate for standard atrophy metrics. Compared with prior BAG studies,^25^, ^26^, ^32^ which largely relied on clinical diagnoses rather than direct biomarker evidence of pathology, our biomarker‐anchored design links BAG to underlying LB pathology—especially in tandem with AD. To our knowledge, this is the first study to apply BA modeling to biomarker‐confirmed AD/LB co‐pathology, revealing its sensitivity to the compounded effects of α‐synuclein and amyloid‐tau pathologies. Together with the mediation results, BAG not only tracks disease burden but also helps characterize the structural pathway by which LB co‐pathology exacerbates cognitive decline in AD.

This study has limitations that warrant consideration. Potential residual participant overlap between ADNI and NACC cannot be excluded because public releases are de‐identified and no public cross‐database overlap lists are available. Because ADNI excludes overt parkinsonism/DLB, the LB+ subgroups here represent CSF SAA‐positive individuals without an overt synucleinopathy syndrome; thus, our findings may not generalize to clinically defined mixed dementia cohorts with parkinsonism or to PD/DLB populations with more widespread LB pathology. MRI‐based measures likely capture only part of LB‐related disease biology, which may also arise through synaptic, network, metabolic, and neurochemical dysfunction, underscoring the need for complementary assessments such as FDG‐PET, diffusion MRI, or functional imaging. For example, recent FDG‐PET studies suggest that mixed AD/LB can show posterior metabolic abnormalities exceeding what is visible as atrophy on MRI.^70^, ^72^ AD−LB+ subgroup was substantially smaller than others, reducing statistical power; accordingly, findings involving AD−LB+ should be interpreted cautiously. The CI AD−LB− subgroup should not be considered pathology‐free, but rather etiologically heterogeneous, potentially reflecting non‐AD/non‐LB or mixed processes not captured by the assayed biomarkers, including cerebrovascular disease, TDP‐43‐related pathology, or other neurodegenerative contributors. Some individuals may also harbor subthreshold pathology burden or anatomically restricted LB pathology that is less sensitively detected by CSF SAA. The intricacies of how TDP‐43 or vascular pathologies, which might intersect with AD+LB+ also remain to be explored. Furthermore, BAG remains an indirect measure of neurodegeneration, and saliency maps should be interpreted cautiously as model‐attribution patterns rather than direct measures of biology or atrophy. Our volumetric analyses were limited to selected ROIs from the Desikan–Killiany atlas; future investigations incorporating additional relevant regions defined by alternative brain atlases may provide a more comprehensive characterization of AD/LB co‐pathology‐related neurodegeneration. Finally, mediation results should be interpreted cautiously given the observational design.

From a clinical and translational standpoint, our results underscore the importance of identifying LB pathology in individuals with AD. Failure to consider LB status could overlook individuals at risk for more aggressive progression, potentially confounding clinical trial outcomes or real‐world interventions. Emerging disease‐modifying therapies targeting Aβ and tau may show reduced clinical efficacy if α‐synuclein simultaneously drives neuronal loss and cognitive deterioration, whereas recognition of mixed proteinopathy may motivate combination therapies tackling multiple proteinopathies at once. These findings support more integrated therapeutic approaches for diagnosis, monitoring, and treatment in coexistent neurodegenerative disease.

## AUTHOR CONTRIBUTIONS

**Babak Ahmadi**: conceptualization, methodology, algorithm development, data collection, data processing, investigation, writing—original draft, and writing—review and editing. **Melissa Armstrong**: review and editing. Breton M. Asken: review and editing. **Mostafa Reisi‐Gahrooei**: review and editing. **Abbas Babajani‐Feremi**: conceptualization, methodology, data collection, critically review, and editing.

## CONFLICT OF INTEREST STATEMENT

The authors declare no conflicts of interest. Author disclosures are available in the Supporting Information.

## CODE AVAILABILITY STATEMENT

The code used in this study is publicly available in the associated GitHub repository||.

## ETHICS STATEMENT

The study was approved by the Institutional Review Board (IRB) of the University of Florida to access archival data.

## Supporting information




**Supporting Information**: alz71593‐sup‐0001‐ICMJE

**Supporting Information**: alz71593‐sup‐0002‐SupMat

## Data Availability

All data used in this study are publicly available from the sources identified in the paper.

## Footnotes

*
*The ADNI (adni.loni.usc.edu) was launched in 2003 as a public‐private partnership, led by Principal Investigator Michael W. Weiner, MD. The primary goal of ADNI has been to test whether serial magnetic resonance imaging (MRI), positron emission tomography (PET), other biological markers, and clinical and neuropsychological assessment can be combined to measure the progression of mild cognitive impairment (MCI) and early Alzheimer's disease (AD). For up‐to‐date information, see* www.adni‐info.org.

† Obtained from the NACC database (available at [https://naccdata.org]).

‡ Obtained from the CamCAN repository (available at http://www.mrc‐cbu.cam.ac.uk/datasets/camcan/).

§ Provided by the Human Connectome Project in Aging (HCP‐Aging), accessible at [https://www.humanconnectome.org/study/hcp‐lifespan‐aging].

||
https://github.com/BabakAhmadi98/DL_BrainAge_CoPath
