# Supplementary material for: Deep learning‐based MRI analysis reveals Lewy body co‐pathology accelerates brain aging in Alzheimer's disease
Source: Alzheimers Dement. 2026 Jul 15;22(7):e71593. doi: 10.1002/alz.71593 (PMC13370797; doi:10.1002/alz.71593)
Supplement: Supplementary file 2 — Supporting Information: alz71593‐sup‐0002‐SupMat [file ALZ-22-e71593-s002.pdf]

SUPPLEMENTARY MATERIALS

1. Cohort characteristics

The cognitively unimpaired dataset was drawn from five independent cohorts—NACC (*n* = 2,304), ADNI (*n* = 348), AIBL (*n* = 359), CamCAN (*n* = 623), and HCP-A (*n* = 721)—and restricted to one scan per participant to minimize data leakage and the risk of overfitting. We first combined these data (*n* = 4,355; 65.9 ± 13.3 years, 63.2% female) and partitioned them into training (80%, *n* = 3,484; 65.8 ± 13.5 years, 63.6% female), validation (10%, *n* = 435; 66.3 ± 12.7 years, 61.4% female), and test (10%, *n* = 436; 66.2 ± 12.9 years, 61.7% female) sets, carefully stratifying by chronological age. The pooled cohort was lifespan-spanning but older-weighted. Across the full model-development cohort, 240 participants were younger than 40 years, 570 were younger than 50 years, and 1,138 were younger than 60 years; in the actual training split, the corresponding counts were 202, 463, and 924. Younger participants were contributed primarily by CamCAN and HCP-A, with additional younger and midlife representation from NACC, whereas ADNI and AIBL were concentrated in older ages. Mean age did not differ significantly across the training, validation, and test subsets (ANOVA $F = 0.461$, $p > 0.05$), and age-bin distributions were likewise preserved (decade-bin $\chi^2 = 8.68$, $p > 0.05$; exact stratification-bin $\chi^2 = 4.92$, $p > 0.05$). **Supplementary Table 1** summarizes the overall characteristics and partitioning of the cognitively unimpaired dataset, whereas **Supplementary Table 2** and **Supplementary Figure 1** are provided to document the age composition of the actual training cohort and to show that age-stratified splitting preserved comparable age-bin composition across the training, validation, and test subsets.

Supplementary Table 1. Cognitively unimpaired (CU) dataset characteristics and partitioning for the deep learning model. A total of 4,355 CU participants (aged 23–100 years) from five major cohorts (NACC, HCP, CamCAN, AIBL, ADNI) were split into training (*n* = 3,484), validation (*n* = 435), and test (*n* = 436) sets via age-stratified sampling. Mean age (± SD) and sex distribution (% female) did not differ significantly among the three subsets (ANOVA and χ^2 tests, $p > 0.05$), confirming balanced splits for model development and evaluation.

	**Full dataset (*n* = 4355)**	**Training (80%, *n* = 3484)**	**Validation (10%, *n* = 435)**	**Test (10%, *n* = 436)**
**Demographics**				
Age	65.89 ± 13.33	65.79 ± 13.46	66.35 ± 12.74	66.18 ± 12.88
Sex, F (%)	2752 (63.2%)	2216 (63.6%)	267 (61.4%)	269 (61.7%)

Supplementary Table 2. Age distribution of the actual training cohort used for brain age model development. The table reports the number of participants contributed by each source cohort, mean age, and decade-bin counts within the training split (23–29, 30–39, 40–49, 50–59, 60–69, 70–79, 80–89, and 90–100 years). The total training cohort comprised 3,484 cognitively unimpaired participants.

Cohort	n	Age, mean±SD	23–29	30–39	40–49	50–59	60–69	70–79	80–89	90–100
**ADNI**	282	71.2 ± 6.0	0	0	0	8	116	134	24	0
**AIBL**	280	71.5 ± 6.2	0	0	0	0	118	132	28	2
**CamCAN**	491	55.4 ± 18.1	50	72	89	66	78	89	47	0
**HCP-A**	569	60.5 ± 15.6	0	52	122	124	102	87	73	9
**NACC**	1,862	68.5 ± 10.7	11	17	50	263	615	651	228	27
**Total training cohort**	3,484	65.8 ± 13.5	61	141	261	461	1,029	1,093	400	38

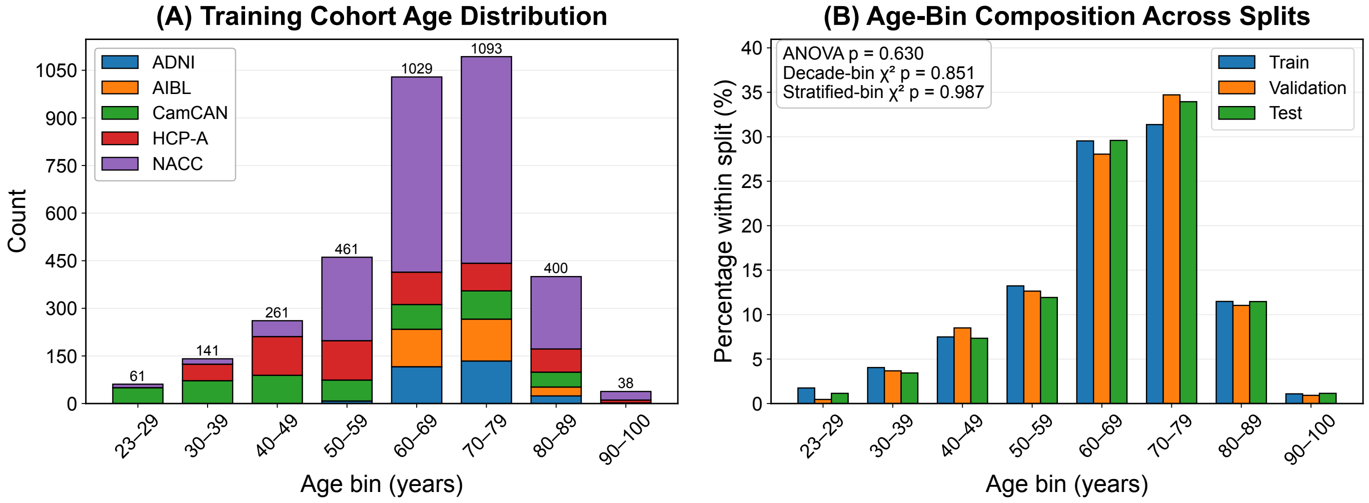

Supplementary Figure 1. Age distribution and split-level age-bin composition of the cognitively unimpaired normative cohort used for brain age model development. **(A)** Age distribution of the actual training split across source cohorts (ADNI, AIBL, CamCAN, HCP-A, and NACC) using decade bins. **(B)** Decade-bin percentages across the training, validation, and test subsets, showing preservation of age

composition after age-stratified splitting. Post hoc analyses showed no significant differences across subsets in mean age or age-bin composition.

Among all 803 cognitively impaired (CI) individuals with AD/LB pathology, 195 fell into the AD-LB- category, 46 in AD-LB+, 396 in AD+LB-, and 166 in AD+LB+. The AD-LB- subgroup was significantly younger than the three biomarker-positive subgroups ($p < 0.01$), although no significant differences were found among the three biomarker-positive groups themselves. Therefore, in all age-related analyses, chronological age was included as a covariate to control for its potential confounding effects. The proportion of female participants varied among groups, being lowest in AD-LB+ (28.3% *vs.* 39.2–42.4%), and pairwise comparisons showed significant differences between AD-LB+ and each of the other groups (all $p < 0.05$ after multiple test correction). As expected, the highest prevalence of APOE-ε4 carriers appeared in the AD+ groups (70.5% in AD+LB+ and 65.9% in AD+LB). The cognitively impaired participants spanned mild cognitive impairment (MCI) and dementia, with significantly more individuals manifesting dementia in the AD+ groups.

2. Brain age estimation in cognitively unimpaired individuals

We trained a 3D-DenseNet deep learning model to estimate brain age from T1-weighted MRI scans of cognitively unimpaired individuals, encompassing a broad age range (23–100 years), ensuring robust capture of normative aging trajectories.

After training, we evaluated the model on the held out cognitively unimpaired test set (n_{test}=436), which included scans from all five cohorts (n_{NACC} = 242, n_{ADNI} = 31, n_{AIBL} = 38, n_{CamCAN} = 67, n_{HCP} = 58). This diverse test set assessed the model's ability to generalize across site- and population-level differences. Prior to final performance reporting, we applied a linear bias correction for chronological age and sex. With bias correction in place, the model achieved an average brain age gap of 0.16 ± 0.22 years and mean absolute error (MAE) of 3.74 ± 0.13 years, a coefficient of determination (R^2) of 0.88, and a Spearman's correlation (*r*) of 0.92 between predicted and chronological ages. The high R^2 and strong correlation highlight the model's capacity to capture the main trajectory of healthy brain aging across multiple cohorts. Notably, the relatively small average gap (close to zero) after bias correction speaks to the robustness of the model's error distribution. To make cross-cohort generalization explicit within this held-out CU test set, we additionally examined performance separately in each source cohort. Across ADNI, AIBL, NACC, CamCAN, and HCP-A, MAE ranged from 2.47 to 4.60 years, Spearman's r from 0.83 to 0.96, and calibration slopes from 0.93 to 1.01, indicating that predictions closely tracked chronological age in each cohort rather than being driven by a single recruitment source

(**Supplementary Table 3**; **Supplementary Figure 2**). These held-out cohort-stratified results complement the stricter LOCO analysis reported in the next Section.

Supplementary Table 3. Cohort-stratified performance of the brain age model in the held-out cognitively unimpaired (CU) test set. The pooled held-out CU test set (*n* = 436) was stratified by source cohort (ADNI, AIBL, NACC, CamCAN, and HCP-A), and age- and sex-bias-corrected predicted brain ages were compared with chronological age within each cohort. Reported metrics include sample size, age distribution, mean absolute error (MAE), root mean squared error (RMSE), coefficient of determination (R^2), Spearman's correlation (r), and the calibration slope/intercept from linear regression of predicted age on chronological age.

Cohort	n	Age, mean ± SD (years)	MAE (years)	RMSE (years)	R^2	Spearman's r	Calibration slope	Calibration intercept
**Pooled CU test set**	436	66.184 ± 12.876	3.742	4.602	0.883	0.923	0.981	1.387
**ADNI**	31	71.452 ± 6.668	2.468	3.161	0.816	0.900	1.013	-0.691
**AIBL**	38	72.447 ± 5.867	2.784	3.690	0.698	0.834	0.933	5.737
**NACC**	242	69.182 ± 10.034	3.756	4.595	0.814	0.886	0.957	3.026
**CamCAN**	67	55.305 ± 17.088	4.081	4.936	0.918	0.960	0.966	1.430
**HCP-A**	58	59.328 ± 13.761	4.603	5.356	0.871	0.916	1.010	0.160

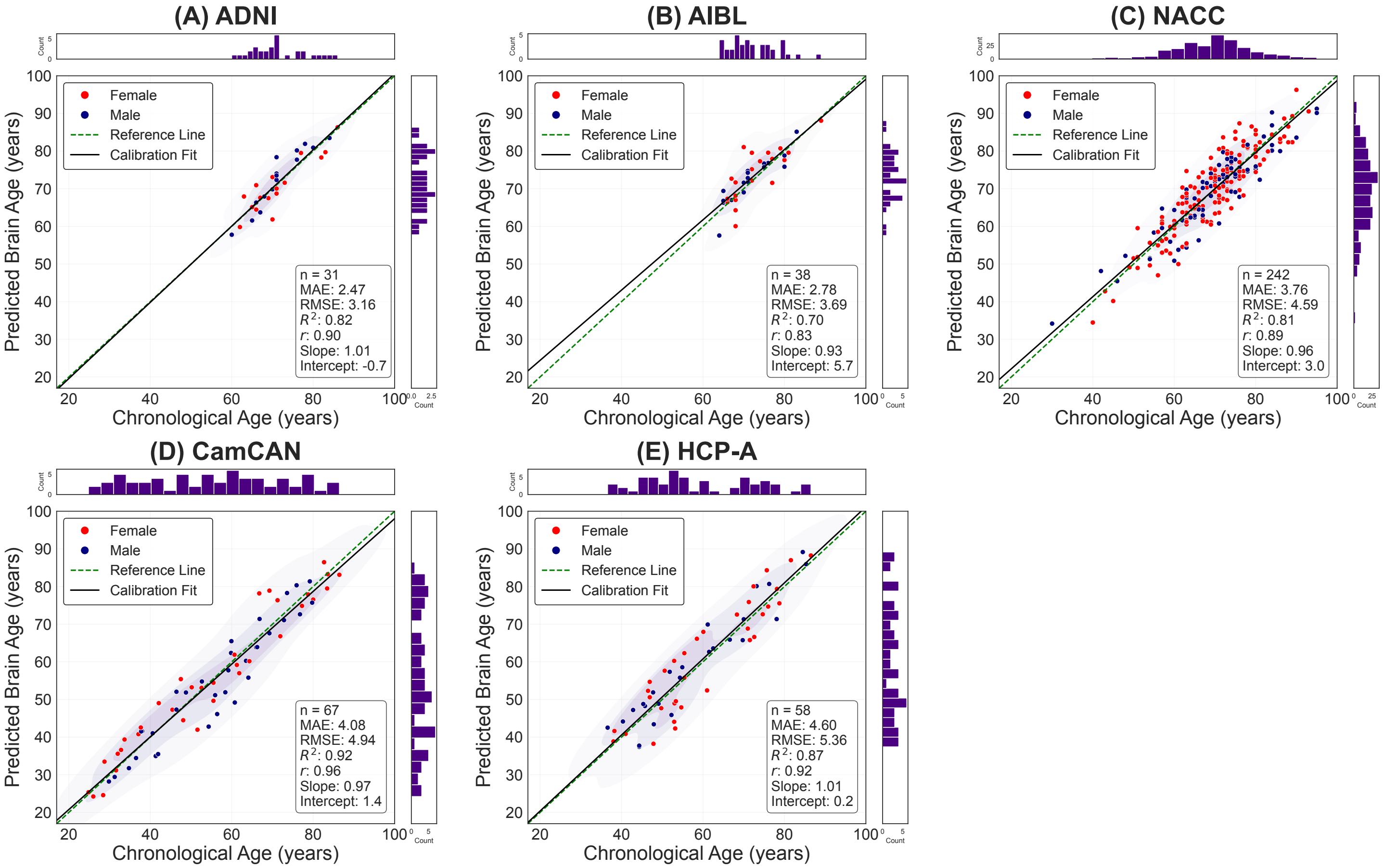

Supplementary Figure 2. Cohort-stratified calibration plots for the held-out cognitively unimpaired (CU) test set. Predicted brain age is plotted against chronological age separately for ADNI, AIBL, NACC, CamCAN, and HCP-A within the held-out CU test set used in **Figure 2A** of the main text. Each point represents a scan and is colored by sex; the dashed diagonal indicates the 1:1 reference line, and the solid black line shows the cohort-specific calibration fit. Histograms along the axes depict the marginal age and predicted-age distributions. Across cohorts, calibration slopes remained close to unity (0.93–1.01), supporting consistent held-out generalization rather than a cohort-specific artefact.

Next, we applied the model to the longitudinal scans of the cognitively unimpaired individuals in the ADNI dataset (excluding those participants used in the model training phase to avoid leakage). This step was aimed to test the model's reliability in capturing normal aging in the ADNI cohort and to generate a reference point for subsequent comparisons with AD/LB pathology subgroups. After bias correction, the average brain age gap was 0.12 ± 0.10 years and the MAE was 2.19 ± 0.06 years, suggesting robust performance on 691 scans of the cognitively unimpaired data in the ADNI cohort. The overall R^2 was 0.85, with r = 0.92, reaffirming the model's ability to track aging patterns in this population.

3. Model generalizability and cross-cohort validation

To rigorously evaluate the generalizability of our brain age model and its robustness to site- and population-level differences, we conducted a Leave-One-Cohort-Out (LOCO) cross-validation. The cognitively unimpaired dataset, comprising five distinct cohorts (NACC, ADNI, AIBL, CamCAN, and HCP-A), was partitioned five times. In each partition, one full cohort was held out as a completely independent test set, while the remaining four cohorts were combined and split into training (80%) and validation (20%) sets using the same age-stratified sampling as in our primary analysis. The 3D-DenseNet model was trained from scratch on each of the five training sets.

Performance was evaluated using the MAE between predicted and chronological age. To account for demographic differences between the training pool and the held-out test cohort, we quantified the age-distribution shift using the standardized mean difference (SMD). Furthermore, to provide an age-aligned comparison of performance, we applied a linear bias correction. The correction parameters were derived solely from the development data (validation sets) of each LOCO fold and subsequently applied to the corresponding held-out test cohort. We report the corrected MAE calculated on the common age band (subjects within the overlapping 5th to 95th age percentiles of the development and test sets).

The LOCO cross-validation demonstrated the strong generalizability of our brain age prediction model. The model performed exceptionally well when tested on held-out cohorts with age distributions similar to the training pool. For example, when the ADNI and AIBL cohorts were held out, the model achieved raw test MAEs in the range of 3.5–4 years.

Performance variation across the five folds was largely explained by the demographic mismatch between the training data and the held-out test set. The CamCAN and HCP-A cohorts, which are significantly younger than the multi-cohort training pool (SMD = 0.95 and 0.51, respectively), yielded higher raw MAEs. However, after applying an age- and sex-based bias correction and evaluating performance on the common age band, the MAEs across cohorts became more consistent: ADNI (3.43 y), AIBL (3.89 y), NACC (4.62 y), HCP-A (5.14 y), and CamCAN (5.25 y). Details are provided in **Supplementary Table 4**. These results confirm that the model effectively captures true features of brain aging and is not overfitted to site-specific characteristics. The consistent performance on age-aligned data underscores its robustness and suitability for application across diverse datasets.

Supplementary Table 4. Leave-One-Cohort-Out (LOCO) cross-validation results. Performance of the brain age model when trained on four cohorts and tested on a fifth, entirely held-out cohort. The age distribution shift is quantified by the standardized mean difference (SMD). Corrected MAE is calculated

on the common age band (overlapping 5-95th percentiles) after applying a bias correction derived from the development data.

Held-out cohort	*n* (Train/Val)	*n* (Test)	Age (Train/Val, mean ± SD)	Age (Test, mean ± SD)	Age shift (SMD)	Test MAE (corrected, common age band; years)
**ADNI**	4,007	348	65.3 ± 13.8 y	71.3 ± 6.2 y	0.44	3.43
**AIBL**	3,996	359	65.3 ± 13.8 y	71.7 ± 6.1 y	0.48	3.89
**NACC**	1,995	2,360	62.6 ± 15.5 y	68.6 ± 10.7 y	0.45	4.62
**HCP**	3,634	721	66.9 ± 12.7 y	60.2 ± 15.6 y	0.51	5.14
**CamCAN**	3,732	623	67.5 ± 11.7 y	55.4 ± 17.9 y	0.95	5.25

4. Benchmarking against brainageR

Since our central question required an anatomically interpretable brain age signal to relate global deviations to specific atrophy and cognition, we used a 3D-CNN-based model, whose attributions can be examined voxelwise (**Main Figure 5, Supplementary Figure 3**) and then tested with longitudinal ROI models. However, for completeness, we compared our model's predictive performance with the commonly used brainageR v2.1[1], using the same CU test splits across ADNI, AIBL, CamCAN, HCP-A, and NACC.

Regarding the implementation, brainageR performs SPM12 segmentation/normalization, PCA (≈80% variance; 435 PCs), and Gaussian Process Regression to predict age, using the pre-trained v2.1 model "as is." As noted in the package documentation[1], AIBL is part of the v2.1 training set, whereas ADNI, HCP-A, NACC, and CamCAN are not. Our DL evaluation followed the Methods protocol (one scan per CU participant; strict subject-level split); for brainageR we applied the model to the same held-out test subjects. We report MAE and the mean BAG for each cohort.

Our DL model produced lower MAE than brainageR in four cohorts outside the brainageR training set—ADNI (DL: 2.47 ± 0.36 y *vs.* brainageR: 5.49 ± 0.72 y), CamCAN (4.08 ± 0.34 y *vs.* 5.09 ± 0.46 y), HCP-A (4.60 ± 0.36 y *vs.* 5.52 ± 0.56 y), and NACC (3.76 ± 0.17 y *vs.* 6.48 ± 0.39 y). In AIBL, which is included in the brainageR training set, brainageR performed slightly better (DL: 2.78 ± 0.40 y *vs.* brainageR: 2.10 ± 0.61 y). Mean BAGs from brainageR were modestly negative in several cohorts (e.g., ADNI -1.65 y; CamCAN -1.31 y; NACC -3.60 y; HCP-A -3.20 y), whereas the DL BAGs were closer to zero (e.g., ADNI +0.22 y; CamCAN -0.45 y; NACC +0.05 y). These results indicate that our DL model is competitive in accuracy across cohorts not used to train

[1] https://github.com/james-cole/brainageR

brainageR and, crucially, provides the whole-brain interpretability leveraged in our saliency, regional atrophy, and cognition analyses.

Supplementary Table 5. Head-to-head accuracy in CU test sets (MAE in years; mean ± SE) for our deep learning model versus brainageR v2.1.

Cohort	Included in brainageR v2.1 training corpus?	brainageR MAE	brainageR BAG mean	DL MAE	DL BAG mean
**ADNI**	No	5.49 ± 0.72	-1.65 ± 1.19	2.47 ± 0.36	0.22 ± 0.58
**CamCAN**	No	5.09 ± 0.46	-1.31 ± 0.75	4.08 ± 0.34	-0.45 ± 0.60
**AIBL**	Yes	2.10 ± 0.61	0.54 ± 0.69	2.78 ± 0.40	0.91 ± 0.59
**HCP-A**	No	5.52 ± 0.56	-3.20 ± 0.79	4.60 ± 0.36	0.76 ± 0.70
**NACC**	No	6.48 ± 0.39	-3.60 ± 0.53	3.76 ± 0.17	0.05 ± 0.30

5. Reduced-capacity architecture sensitivity analysis

We evaluated robustness to model capacity by retraining two shallower 3D-DenseNet variants (2,4,8,6 and 2,3,6,4 dense-block configurations, instead of the primary 3,6,12,8) using identical preprocessing, splits, optimization, and bias-correction procedures. Both reduced-capacity models remained usable on the CU test set but showed higher prediction error than the primary model (**Supplementary Tables 6A** and **6B)**. In downstream analyses, both models preserved the main cross-sectional and baseline co-pathology BAG findings, and the medium-capacity model also preserved the AD+LB+ versus AD+LB- longitudinal slope difference. In the smallest model, that specific longitudinal contrast attenuated, consistent with reduced sensitivity of the model rather than reversal of the overall pathology pattern (**Supplementary Tables 7A** and **7B**).

Supplementary Table 6. Sensitivity analysis of reduced-capacity 3D-DenseNet architectures in cognitively unimpaired cohorts. The primary model (3,6,12,8 dense-block configuration) was compared with two shallower variants (2,4,8,6 and 2,3,6,4) trained with identical preprocessing, age-stratified splits, optimization, early-stopping criteria, and bias-correction procedures. Shown are approximate trainable parameter counts and normative performance metrics in **(A)** the held-out multicohort CU test set and **(B)** the CU ADNI longitudinal subset.

(A)

Model	Dense-block layers	Approx. trainable parameters	Multicohort CU test set MAE ± SE (years)	Multicohort CU test set R^2	Multicohort CU test set Spearman r
**Full**	3,6,12,8	251.1M	3.74 ± 0.13	0.88	0.92
**Medium**	2,4,8,6	110.9M	4.39 ± 0.18	0.86	0.91

**Small**	2,3,6,4	60.5M	4.54 ± 0.21	0.86	0.91

(B)

Model	Dense-block layers	Approx. trainable parameters	Independent CU ADNI longitudinal subset MAE ± SE (years)	Independent CU ADNI longitudinal subset R^2	Independent CU ADNI longitudinal subset Spearman r
**Full**	3,6,12,8	251.1M	2.19 ± 0.06	0.85	0.92
**Medium**	2,4,8,6	110.9M	2.35 ± 0.06	0.84	0.90
**Small**	2,3,6,4	60.5M	2.47 ± 0.09	0.81	0.89

Supplementary Table 7. Robustness of AD/LB pathology BAG findings across reduced-capacity architectures. Shown are **(A)** the mean absolute error values are for each pathology subgroup and **(B)** the overall group effect and the key AD+LB+ versus AD+LB- contrasts from the baseline BAG model, and longitudinal BAG-slope model. Both shallower models preserved the main cross-sectional and baseline co-pathology pattern, whereas the smallest model showed attenuation of the most specific longitudinal AD+LB+ versus AD+LB- slope contrast.

(A)

Model	AD-LB- MAE ± SE (years)	AD-LB+ MAE ± SE (years)	AD+LB- MAE ± SE (years)	AD+LB+ MAE ± SE (years)
**Full**	4.29 ± 0.17	4.36 ± 0.36	5.75 ± 0.16	6.96 ± 0.27
**Medium**	4.48 ± 0.27	4.61 ± 0.56	5.63 ± 0.22	6.85 ± 0.31
**Small**	4.70 ± 0.28	5.20 ± 0.58	5.99 ± 0.23	7.23 ± 0.32

(B)

Model	Overall group effect (GEE)	Baseline BAG model: AD+LB+ vs AD+LB-	Longitudinal BAG-slope model: AD+LB+ vs AD+LB-
**Full**	$\chi^2(4)=156.07$, $p<10^{-32}$	Δ = +1.25 years, SE = 0.44, $p < 0.01$	Δβ = +0.27 years/year, SE = 0.10, $p < 0.05$
**Medium**	$\chi^2(4)=154.67$, $p<10^{-32}$	Δ = +1.37 years, SE = 0.45, $p < 0.01$	Δβ = +0.25 years/year, SE = 0.10, $p < 0.05$

**Small**	$\chi^2(4)=184.77$, $p<10^{-38}$	$\Delta = +1.52$ years, SE = 0.45, $p < 0.01$	$\Delta\beta = +0.09$ years/year, SE = 0.10, $p > 0.05$

6. Brain age estimation in pathological groups

Deploying the model trained with normative cohorts, and applying the same bias correction used in cognitively unimpaired participants, we estimated brain age in four cognitively impaired AD/LB pathology subgroups (AD-LB-, AD-LB+, AD+LB-, and AD+LB+). **Figure 3** in the main manuscript shows the distribution of these bias-corrected brain age gaps, and corresponding summary statistics are presented in **Supplementary Table 8**. All AD/LB pathology subgroups showed significantly elevated brain age gaps relative to the cognitively unimpaired reference—indicating that their structural MRI-derived brain ages exceeded chronological ages to a greater extent than in cognitively unimpaired individuals. Notably, the co-pathology subgroup (AD+LB+) exhibited the largest (6.61 ± 0.3 years; MAE = 6.96 ± 0.27), exceeding that of both AD+LB- (4.32 ± 0.21 years; MAE = 5.75 ± 0.16) and AD-LB+ (1.98 ± 0.55 years; MAE = 4.36 ± 0.36), as well as AD-LB- (1.83 ± 0.26 years; MAE = 4.29 ± 0.17).

Supplementary Table 8. Summary of brain age estimation accuracy and brain age gap across all groups. Shown are the mean absolute error (MAE ± standard error [SE]), and mean bias-corrected brain age gap (BAG ± SE): the cognitively unimpaired (CU) test set, a longitudinal CU subset from ADNI, and four AD/LB pathology subgroups (AD-LB-, AD-LB+, AD+LB-, AD+LB+). While the CU groups exhibit low MAE and near-zero average BAG, all pathological subgroups show significantly elevated BAG, with the AD+LB+ (co-pathology) subgroup displaying the highest deviation. These findings suggest a progressive increase in structural brain aging when AD and LB pathologies co-occur.

Group	Mean Absolute Error ± SE (years)	Mean Age Gap ± SE (years)
CU Test set	3.74 ± 0.13	0.16 ± 0.22
CU Longitudinal ADNI	2.19 ± 0.06	0.12 ± 0.10
AD-LB-	4.29 ± 0.17	1.83 ± 0.26
AD-LB+	4.36 ± 0.36	1.98 ± 0.55
AD+LB-	5.75 ± 0.16	4.32 ± 0.21
AD+LB+	6.96 ± 0.27	6.61 ± 0.30

7. Effect sizes for key BAG and regional volumetric contrasts

Supplementary Tables 9 and **10** summarize effect sizes for the key BAG and representative regional volumetric contrasts emphasized in the main text of the manuscript. For baseline

comparisons, we report the adjusted mean difference, 95% confidence interval, FDR-adjusted p-value, and Hedges' g. For longitudinal comparisons, we report the adjusted slope difference, 95% confidence interval, FDR-adjusted p-value, and standardized β. Standardized β values for annualized interaction terms should be interpreted alongside the raw adjusted slope differences, because these standardized coefficients are often numerically small even when between-group divergence accumulates meaningfully over follow-up. Across outcomes, the largest and most consistent effects involved the AD+LB+ versus AD+LB- contrast, whereas estimates involving AD-LB+ generally showed wider confidence intervals, consistent with lower precision in that smaller subgroup.

Supplementary Table 9. Baseline effect sizes for key BAG and representative regional volumetric contrasts across AD/LB pathology subgroups. Pairwise baseline contrasts are shown for corrected brain age gap (BAG) and the representative regional volumetric measures emphasized in the main text (medial temporal lobe, basal ganglia, occipital lobe, and middle temporal cortex). Values are reported as Group1 − Group2, together with the adjusted mean difference, 95% confidence interval, FDR-adjusted p-value, and covariate-adjusted Hedges' g. For BAG, a negative difference indicates a higher BAG in Group2. For volumetric measures, a positive difference indicates a larger baseline volume in Group1 (equivalently, lower volume in Group2).

Outcome	Contrast (Group1 − Group2)	Adjusted difference	95% CI	P-value	Hedges' g
**BAG**	AD-LB- − AD-LB+	-0.81 years	-2.31 to 0.70	>0.05	-0.18
**BAG**	AD-LB- − AD+LB-	-2.50 years	-3.41 to -1.60	<0.001	-0.34
**BAG**	AD-LB- − AD+LB+	-3.76 years	-4.85 to -2.66	<0.001	-0.58
**BAG**	AD-LB+ − AD+LB-	-1.70 years	-3.20 to -0.20	<0.05	-0.16
**BAG**	AD-LB+ − AD+LB+	-2.95 years	-4.57 to -1.33	<0.001	-0.41
**BAG**	AD+LB- − AD+LB+	-1.25 years	-2.11 to -0.39	<0.01	-0.22
**Medial Temporal Lobe**	AD-LB- − AD-LB+	0.35 cm^3	-0.36 to 1.05	>0.05	0.15
**Medial Temporal Lobe**	AD-LB- − AD+LB-	0.58 cm^3	0.14 to 1.02	<0.05	0.16

**Medial Temporal Lobe**	AD-LB- − AD+LB+	1.20 cm^3	0.68 to 1.72	<0.001	0.43
**Medial Temporal Lobe**	AD-LB+ − AD+LB-	0.23 cm^3	-0.47 to 0.94	>0.05	0.01
**Medial Temporal Lobe**	AD-LB+ − AD+LB+	0.85 cm^3	0.09 to 1.61	<0.05	0.28
**Medial Temporal Lobe**	AD+LB- − AD+LB+	0.62 cm^3	0.21 to 1.03	<0.01	0.28
**Basal Ganglia**	AD-LB- − AD-LB+	0.45 cm^3	-0.25 to 1.16	>0.05	0.21
**Basal Ganglia**	AD-LB- − AD+LB-	0.36 cm^3	-0.07 to 0.80	>0.05	0.12
**Basal Ganglia**	AD-LB- − AD+LB+	0.59 cm^3	0.07 to 1.11	>0.05	0.21
**Basal Ganglia**	AD-LB+ − AD+LB-	-0.09 cm^3	-0.80 to 0.62	>0.05	-0.09
**Basal Ganglia**	AD-LB+ − AD+LB+	0.13 cm^3	-0.63 to 0.89	>0.05	0.00
**Basal Ganglia**	AD+LB- − AD+LB+	0.22 cm^3	-0.19 to 0.63	>0.05	0.10
**Occipital Lobe**	AD-LB- − AD-LB+	1.26 cm^3	-0.23 to 2.75	>0.05	0.27
**Occipital Lobe**	AD-LB- − AD+LB-	1.10 cm^3	0.17 to 2.02	>0.05	0.19
**Occipital Lobe**	AD-LB- − AD+LB+	1.04 cm^3	-0.06 to 2.14	>0.05	0.17
**Occipital Lobe**	AD-LB+ − AD+LB-	-0.16 cm^3	-1.66 to 1.33	>0.05	-0.09
**Occipital Lobe**	AD-LB+ − AD+LB+	-0.21 cm^3	-1.82 to 1.39	>0.05	-0.11
**Occipital Lobe**	AD+LB- − AD+LB+	-0.05 cm^3	-0.92 to 0.82	>0.05	-0.02
**Middle Temporal**	AD-LB- − AD-LB+	0.79 cm^3	0.00 to 1.58	>0.05	0.34

**Middle Temporal**	AD-LB- − AD+LB-	1.00 cm^3	0.51 to 1.49	<0.001	0.30
**Middle Temporal**	AD-LB- − AD+LB+	1.14 cm^3	0.56 to 1.73	<0.001	0.39
**Middle Temporal**	AD-LB+ − AD+LB-	0.21 cm^3	-0.58 to 1.00	>0.05	-0.02
**Middle Temporal**	AD-LB+ − AD+LB+	0.35 cm^3	-0.50 to 1.20	>0.05	0.02
**Middle Temporal**	AD+LB- − AD+LB+	0.14 cm^3	-0.32 to 0.60	>0.05	0.04

Supplementary Table 10. Longitudinal effect sizes for key BAG and representative regional volumetric contrasts across AD/LB pathology subgroups. Pairwise longitudinal contrasts are shown for corrected BAG and the representative regional volumetric measures emphasized in the main text. Values are reported as Group1 – Group2, together with the adjusted slope difference, 95% confidence interval, FDR-adjusted p-value, and standardized β. For BAG, a negative slope difference indicates a faster BAG increase in Group2. For volumetric measures, a positive slope difference indicates that Group2 has a more negative slope and therefore steeper longitudinal atrophy than Group1.

Outcome	Contrast (Group1 − Group2)	Adjusted slope difference	95% CI	P-value	Standardized β
**BAG**	AD-LB- − AD-LB+	-0.16 years/year	-0.44 to 0.13	>0.05	-0.03
**BAG**	AD-LB- − AD+LB-	-0.30 years/year	-0.41 to -0.18	<0.001	-0.06
**BAG**	AD-LB- − AD+LB+	-0.56 years/year	-0.78 to -0.35	<0.001	-0.11
**BAG**	AD-LB+ − AD+LB-	-0.14 years/year	-0.41 to 0.13	>0.05	-0.03
**BAG**	AD-LB+ − AD+LB+	-0.41 years/year	-0.73 to -0.08	<0.05	-0.08
**BAG**	AD+LB- − AD+LB+	-0.27 years/year	-0.46 to -0.08	<0.05	-0.05
**Medial Temporal Lobe**	AD-LB- − AD-LB+	0.08 cm^3/year	-0.03 to 0.19	>0.05	0.03

**Medial Temporal Lobe**	AD-LB- − AD+LB-	0.19 cm^3/year	0.12 to 0.25	<0.001	0.06
**Medial Temporal Lobe**	AD-LB- − AD+LB+	0.30 cm^3/year	0.21 to 0.39	<0.001	0.10
**Medial Temporal Lobe**	AD-LB+ − AD+LB-	0.11 cm^3/year	0.03 to 0.18	<0.01	0.04
**Medial Temporal Lobe**	AD-LB+ − AD+LB+	0.22 cm^3/year	0.12 to 0.33	<0.001	0.08
**Medial Temporal Lobe**	AD+LB- − AD+LB+	0.12 cm^3/year	0.06 to 0.17	<0.001	0.04
**Basal Ganglia**	AD-LB- − AD-LB+	0.03 cm^3/year	-0.10 to 0.17	>0.05	0.01
**Basal Ganglia**	AD-LB- − AD+LB-	0.06 cm^3/year	-0.02 to 0.14	>0.05	0.02
**Basal Ganglia**	AD-LB- − AD+LB+	0.16 cm^3/year	0.04 to 0.28	<0.05	0.06
**Basal Ganglia**	AD-LB+ − AD+LB-	0.03 cm^3/year	-0.06 to 0.12	>0.05	0.01
**Basal Ganglia**	AD-LB+ − AD+LB+	0.13 cm^3/year	-0.00 to 0.25	>0.05	0.05
**Basal Ganglia**	AD+LB- − AD+LB+	0.10 cm^3/year	0.03 to 0.17	<0.05	0.04
**Occipital Lobe**	AD-LB- − AD-LB+	0.02 cm^3/year	-0.25 to 0.29	>0.05	0.00
**Occipital Lobe**	AD-LB- − AD+LB-	0.34 cm^3/year	0.17 to 0.50	<0.001	0.06
**Occipital Lobe**	AD-LB- − AD+LB+	0.54 cm^3/year	0.30 to 0.78	<0.001	0.09
**Occipital Lobe**	AD-LB+ − AD+LB-	0.31 cm^3/year	0.14 to 0.49	<0.001	0.05
**Occipital Lobe**	AD-LB+ − AD+LB+	0.52 cm^3/year	0.27 to 0.76	<0.001	0.09
**Occipital Lobe**	AD+LB- − AD+LB+	0.20 cm^3/year	0.07 to 0.34	<0.01	0.03

**Middle Temporal**	AD-LB- − AD-LB+	0.10 cm^3/year	-0.06 to 0.25	>0.05	0.03
**Middle Temporal**	AD-LB- − AD+LB-	0.30 cm^3/year	0.20 to 0.39	<0.001	0.09
**Middle Temporal**	AD-LB- − AD+LB+	0.52 cm^3/year	0.38 to 0.65	<0.001	0.16
**Middle Temporal**	AD-LB+ − AD+LB-	0.20 cm^3/year	0.09 to 0.31	<0.01	0.06
**Middle Temporal**	AD-LB+ − AD+LB+	0.42 cm^3/year	0.28 to 0.56	<0.001	0.13
**Middle Temporal**	AD+LB- − AD+LB+	0.22 cm^3/year	0.14 to 0.30	<0.001	0.07

8. Sensitivity analysis excluding the AD-LB+ subgroup

To determine whether the small AD-LB+ subgroup drove the primary BAG findings, we repeated the three main BAG analyses after excluding AD-LB+ entirely: the scan-level GEE model, the baseline BAG model, and the longitudinal BAG-slope model. The qualitative pattern was unchanged in all cases, with AD+LB+ remaining significantly higher than AD+LB- (**Supplementary Table 11**). These results indicate that the central co-pathology findings were not driven by the small AD-LB+ subgroup.

Supplementary Table 11. Sensitivity analysis excluding the AD-LB+ subgroup from the primary BAG models. The three primary BAG analyses were repeated after excluding all AD-LB+ participants. For clarity, the table reports the central contrast of interest, AD+LB+ vs AD+LB-, in each model. The persistence of significant differences across all three models indicates that the main co-pathology findings were not driven by the small AD-LB+ subgroup.

Model	Contrast reported	Effect estimate	95% CI	P-value
**All-scan BAG GEE**	AD+LB+ vs AD+LB-	2.06 years	1.16 to 2.95	<0.001
**Baseline BAG (first-visit contrast)**	AD+LB+ vs AD+LB-	1.14 years	0.30 to 1.98	<0.01
**Longitudinal BAG slope**	AD+LB+ vs AD+LB-	0.291 years/year	0.10 to 0.48	<0.01

9. Harmonization sensitivity analysis

The CU training data were assembled from multiple cohorts (i.e., ADNI, AIBL, HCP, NACC, CamCAN). We therefore evaluated whether site differences could bias model training or downstream inferences. We implemented ComBat-GAM in two ways and assessed both site-effect attenuation and biological-signal preservation, then re-ran the main analyses.

We used ComBat-GAM[2] to adjust for site differences while preserving biological covariates. ComBat-GAM is an extension of ComBat[3] that models batch (i.e., site) effects with location-scale adjustments while allowing smooth covariate effects (here, we considered age as a smooth term to account for the nonlinear trajectory of brain changes across the lifespan) and preserving biological covariates. Harmonization parameters were learned on the CU training set only, to prevent information leakage and then applied unchanged to the CU validation/test sets and all AD/LB pathology groups. We conducted two pipelines: (i) harmonization applied to pre-processed images conservatively, before training the brain age model and (ii) harmonization applied post hoc to predicted brain ages.

To quantify site-signal attenuation and biological-signal preservation, we used: (i) domain-predictability of site (5-fold cross validation [CV] accuracy of multinomial logistic regression trained to predict site from principal components (PCs) of the images; chance≈0.20 with five cohorts); (ii) partial R^2(site) and partial R^2(age) computed on top PCs using Ordinary Least Squares (OLS) with adjustment for the other covariates (lower site with stable age indicates successful harmonization without erasing biology); and (iii) structural similarity (SSIM) between original and harmonized images (values near 1 indicate high fidelity). To test whether harmonization changed the predicted brain ages, we performed paired equivalence tests (two one-sided tests; TOST, ±1-year margin), complemented by paired *t*-tests, and Pearson correlations. For the post hoc harmonization we quantified the variance fraction attributable to site in a mixed-effects model of brain age (random site effect; where lower is better).

Relative to unharmonized images, image-level harmonization substantially reduced site signal while preserving age signal and image structure. In CU train/validation/rest, site CV accuracy fell from 0.76/0.67/0.66 to 0.54/0.40/0.42; mean partial R^2(site) on PCs dropped from 0.034/0.043/0.039 to 0.001/0.008/0.007, whereas partial R^2(Age) remained stable at ≈0.026–0.027. SSIM values were 0.984/0.976/0.981, indicating high structural fidelity after harmonization. These diagnostics are consistent with effective removal of site imprinting without erasing biological variation.

Paired within-subject comparisons supported equivalence within ±1 year. For raw brain age (BA), the mean difference (harmonized − baseline) was +0.30 years (paired *t*-test $p = 0.35$),

with TOST $p < 0.05$ and $r = 0.927$. For (age and sex) bias-corrected BA, the mean difference was +0.35 years ($p = 0.35$), with TOST $p < 0.05$ and $r = 0.926$. Thus, image-level harmonization produced BA estimates statistically equivalent to the unharmonized pipeline under a clinically conservative ±1-year margin.

Re-analysis of the ADNI subgroups preserved the qualitative ranking and longitudinal contrasts reported in the main text. AD+LB+ remained the group with the largest deviation from normative ageing and the steepest longitudinal increase in BAG; AD+LB- was intermediate; AD-LB+ and AD-LB- were smaller, mirroring the primary results (**Main Figures 3, 4**). As an example, mean absolute errors with this harmonization were 6.72 (AD+LB+), 5.58 (AD+LB-), 4.82 (AD-LB+), and 4.58 (AD-LB-), closely paralleling the unharmonized values (6.96, 5.75, 4.36, and 4.29, respectively), and leaving all inferences unchanged.

In the second harmonization pipeline (Combat-GAM on predicted brain ages, which are derived from training the deep learning model on unharmonized images), since BA itself already carried little site imprint, BA-only ComBat-GAM left site CV accuracy essentially unchanged in CU train/validation/test (≈0.565/0.542/0.588; values close to the after-harmonization site CV accuracy in the first pipeline), with partial R^2(site) ≈ 0.000–0.003 and stable correlations of BA with age (e.g., Validation $r = 0.916$ to 0.899; Test $r = 0.912$ to 0.895). The variance fraction of site in mixed models was ≈ 0.000 before and after harmonization, indicating that site contributed negligibly to variance in the data, both pre- and post-harmonization, confirming that results were not confounded by site effects. Additionally, paired equivalence tests indicated that BA-only harmonization yields virtually identical predictions: for raw BA, mean difference +0.00039 years (paired t-test $p < 0.01$; TOST $p < 0.001$; $r \approx 1$); for bias-corrected BA, mean difference +0.00046 years ($p < 0.01$; TOST $p < 0.001$; $r \approx 1$). In other words, this harmonization has no practical impact on predicted ages and leaves the downstream group contrasts and trajectories unchanged.

As an exploratory check we combined Nyúl histogram normalization with RAVEL[4] (CSF control). However, site predictability did not improve at all (Train 0.76 to 0.76; Validation 0.67 to 0.65; Test 0.66 to 0.66) and SSIM to original images dropped (≈ 0.54), indicating substantial structural distortion. Given these trade-offs, we did not pursue this pipeline further.

10. Interpretability of brain age prediction model

We used gradient-based saliency mapping to identify the brain regions most influencing our 3D-DenseNet model's brain age predictions. Gradients were computed to assess voxel-level sensitivity, with Gaussian smoothing applied to enhance interpretability. Saliency maps were

generated for all participants and averaged within each diagnostic group for comparison against cognitively unimpaired individuals to distinguish neurodegeneration-related regions in the AD/LB pathology subgroups. These difference maps revealed that the co-pathology group (AD+LB+) displayed more pronounced saliency among different brain regions, compared to other subgroups. Moreover, comparing co-pathology subgroup with isolated AD pathology subgroup showed heightened saliency near the cholinergic basal forebrain, as well as in parts of the right cingulum. Next, using the Automated Anatomical Labeling (AAL) atlas, we computed the average saliency values for each brain region to evaluate their relative importance. **Supplementary Tables 12–15** detail the regions exhibiting the highest mean saliency values across the following comparisons: (a) AD+LB+ *vs.* Normal Aging, (b) AD+LB- *vs.* Normal Aging, (c) AD-LB+ *vs.* Normal Aging, and (d) AD-LB- *vs.* Normal Aging.

Supplementary Table 12. Region-based mean saliency values, using AAL atlas for AD+LB+ *vs.* Normal aging (i.e., CU) comparison.

Region name	Region number	Mean saliency
Amygdala_R 4202	42	0.012160149
Amygdala_L 4201	41	0.012017412
Hippocampus_R 4102	38	0.011542726
Hippocampus_L 4101	37	0.009637509
ParaHippocampal_R 4112	40	0.006564623
Olfactory_R 2502	22	0.006333057
Cingulum_Ant_R 4002	32	0.006192116
Calcarine_R 5002	44	0.006122886
Olfactory_L 2501	21	0.005885927
Cingulum_Post_R 4022	36	0.005779585
Fusiform_R 5402	56	0.005695175
Thalamus_L 7101	77	0.005512096
Calcarine_L 5001	43	0.005427618
Lingual_L 5021	47	0.005319608
Temporal_Mid_R 8202	86	0.005246252
Cingulum_Mid_R 4012	34	0.005215452
Fusiform_L 5401	55	0.005197588
Occipital_Mid_R 5202	52	0.005189824
Occipital_Inf_R 5302	54	0.004998993
Precuneus_R 6302	68	0.004971811
SupraMarginal_R 6212	64	0.004915192
Lingual_R 5022	48	0.00490186
Cingulum_Post_L 4021	35	0.004890117
Cuneus_R 5012	46	0.004843625

Precuneus_L 6301	67	0.004797317
Temporal_Sup_R 8112	82	0.004726816
Rolandic_Oper_R 2332	18	0.004706172
Occipital_Sup_R 5102	50	0.004692861
Parietal_Inf_L 6201	61	0.004690736
Occipital_Mid_L 5201	51	0.004665844
Angular_R 6222	66	0.004566956
Cuneus_L 5011	45	0.004554632
Temporal_Inf_R 8302	90	0.004438887
Angular_L 6221	65	0.004430278
Temporal_Mid_L 8201	85	0.004420687
Frontal_Med_Orb_L 2611	25	0.004417704
Precentral_R 2002	2	0.004403006
Cingulum_Ant_L 4001	31	0.004296766
Vermis_8 9150	114	0.004196622
Insula_R 3002	30	0.004161854
Cerebelum_9_R 9072	106	0.00413233
Vermis_3 9110	110	0.004097534
Occipital_Sup_L 5101	49	0.004080345
Temporal_Inf_L 8301	89	0.004008973
Cingulum_Mid_L 4011	33	0.003992106

Supplementary Table 13. Region-based mean saliency values, using AAL atlas for AD+LB- *vs*. CU comparison.

Region name	Region number	Mean saliency
Amygdala_L 4201	41	0.010837
Amygdala_R 4202	42	0.0099
Hippocampus_R 4102	38	0.009578
Hippocampus_L 4101	37	0.00789
Cingulum_Post_L 4021	35	0.005151
Lingual_L 5021	47	0.004985
Calcarine_R 5002	44	0.004955
Cingulum_Ant_R 4002	32	0.004788
SupraMarginal_R 6212	64	0.004711
ParaHippocampal_R 4112	40	0.004692
Temporal_Mid_R 8202	86	0.004638
Calcarine_L 5001	43	0.004577
Occipital_Inf_R 5302	54	0.004422
Cingulum_Post_R 4022	36	0.004333
Cuneus_R 5012	46	0.004307
Occipital_Mid_R 5202	52	0.004285

Angular_R 6222	66	0.004232
Fusiform_L 5401	55	0.004228
Olfactory_L 2501	21	0.004204
Thalamus_L 7101	77	0.004153
Fusiform_R 5402	56	0.004144
Temporal_Sup_R 8112	82	0.004054
Cuneus_L 5011	45	0.004038
Olfactory_R 2502	22	0.004025
Lingual_R 5022	48	0.003954

Supplementary Table 14. Region-based mean saliency values, using AAL atlas for AD-LB+ *vs*. CU comparison.

Region name	Region number	Mean saliency
Hippocampus_L 4101	37	0.007823
Hippocampus_R 4102	38	0.007373
Amygdala_R 4202	42	0.00612
Amygdala_L 4201	41	0.005658
Vermis_7 9140	113	0.005541
Vermis_6 9130	112	0.005339
Olfactory_L 2501	21	0.004967
Thalamus_L 7101	77	0.004924
Lingual_L 5021	47	0.00473
Calcarine_L 5001	43	0.004576
Calcarine_R 5002	44	0.004511
Temporal_Mid_R 8202	86	0.004503
Cerebelum_6_L 9041	99	0.004395
Cuneus_R 5012	46	0.004281
Vermis_8 9150	114	0.004239
Fusiform_R 5402	56	0.00411
ParaHippocampal_R 4112	40	0.004039
Occipital_Inf_R 5302	54	0.003971
Fusiform_L 5401	55	0.003891
Occipital_Mid_R 5202	52	0.003843
SupraMarginal_R 6212	64	0.003784
Rolandic_Oper_R 2332	18	0.003709
Cerebelum_6_R 9042	100	0.00364
Olfactory_R 2502	22	0.003551
SupraMarginal_L 6211	63	0.003525
Precuneus_R 6302	68	0.003484
Lingual_R 5022	48	0.00343

Cingulum_Post_L 4021	35	0.003239
Temporal_Inf_L 8301	89	0.003101
Precentral_R 2002	2	0.003082
Cerebelum_9_R 9072	106	0.003077
Temporal_Inf_R 8302	90	0.003065
Occipital_Sup_R 5102	50	0.003009

Supplementary Table 15. Region-based mean saliency values, using AAL atlas for AD-LB- *vs.* CU comparison.

**Region name**	**Region number**	**Mean saliency**
Hippocampus_R 4102	38	0.005791
Amygdala_L 4201	41	0.005225
Amygdala_R 4202	42	0.005183
Hippocampus_L 4101	37	0.003883
Lingual_L 5021	47	0.003762
Vermis_8 9150	114	0.00376
Thalamus_L 7101	77	0.003379
Cingulum_Post_R 4022	36	0.003217
SupraMarginal_R 6212	64	0.003167
Temporal_Mid_R 8202	86	0.003085
Calcarine_L 5001	43	0.003053
Rolandic_Oper_R 2332	18	0.003042
Calcarine_R 5002	44	0.003039

11. Validation of saliency mapping methods with SmoothGrad

To ensure our interpretability findings were robust and not dependent on a single attribution method, we validated our primary gradient-based saliency results using SmoothGrad[5]. The SmoothGrad technique is designed to produce visually sharper and less noisy attribution maps by averaging the gradients computed from multiple noisy copies of a single input image.

For each MRI scan, we generated 30 noisy replicas by adding Gaussian noise. The standard deviation of the noise was scaled to 10% of the standard deviation of voxel intensities within the brain mask to ensure meaningful yet non-saturating perturbations. The gradients of the model's brain age output with respect to the input voxels were calculated for each noisy replica, and then were averaged to produce a single, stabilized saliency map. The SmoothGrad-derived difference saliency maps are presented in **Supplementary Figure 3**.

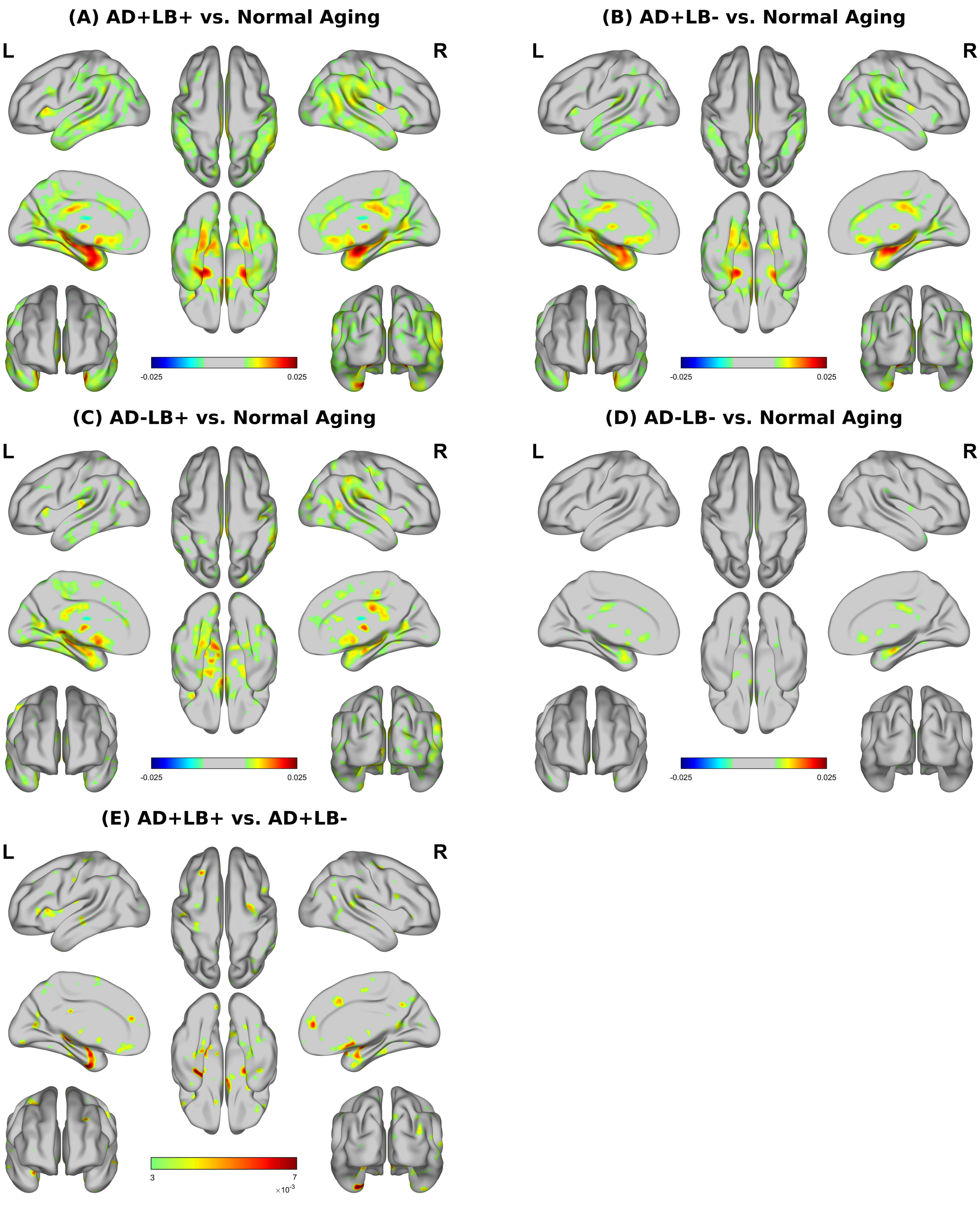

Supplementary Figure 3. Group-level difference saliency maps using SmoothGrad, highlighting regions contributing to elevated brain age in AD/LB subgroups. (A–D) The difference between the averaged saliency for each AD/LB subgroup versus the cognitively unimpaired (CU) reference; warmer colors (yellow–red) indicate stronger contributions to the model's higher brain age predictions in the pathological subgroup. Conversely, cooler colors (blue–cyan) mark areas in which saliency is greater for the cognitively unimpaired cohort. The AD+LB+ demonstrates a more extensive involvement than

AD+LB- or AD-LB+, consistent with an amplified neurodegeneration in co-pathology. **(E)** Direct comparison of AD+LB+ *vs.* AD+LB-, isolating the additional effect of LB on an AD background. Prominent saliencies involve cholinergic basal forebrain, and parts of medial temporal structures, right cingulum, right precuneus, and left fusiform gyrus consistent with a compounded neurodegenerative footprint in co-pathology.

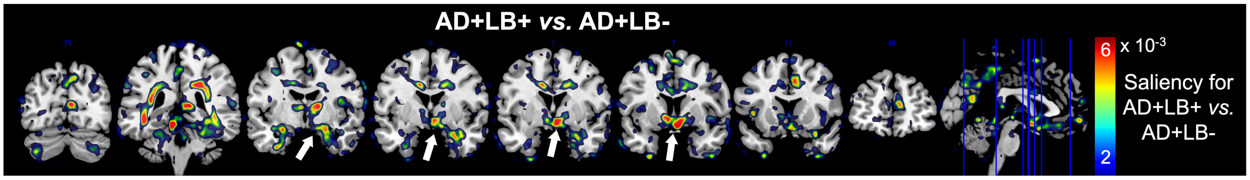

Supplementary Figure 4. **Saliency map comparisons for AD+LB+ *vs.* AD+LB-**. Saliency maps are overlaid on the standard MNI template, and various slices of the coronal view are presented. Comparing AD+LB+ *vs.* AD+LB-, we can observe a positive saliency cluster near the cholinergic basal forebrain (indicated by white arrows), including the Nucleus Basalis of Meynert (NBM), highlighting heightened relevance of these nuclei in distinguishing co-pathology from AD alone.

We quantified the agreement between the original Vanilla and the new SmoothGrad maps at three distinct levels for each pathological group comparison (e.g., AD+LB+ *vs.* CU):

1. **Regional saliency correlation:** We calculated the Spearman's correlation of the mean saliency values across all anatomical regions defined by the standard FreeSurfer parcellation. This atlas, which combines the Desikan-Killiany cortical parcellation with subcortical segmentation, allowed for a comprehensive, region-by-region comparison of the saliency patterns generated by each method.
2. **Top-ranked region overlap:** We identified the top-20 most salient brain regions for each method (ranked by mean saliency) and computed the Dice coefficient to measure their set-wise overlap.
3. **Voxel-wise spatial overlap:** We thresholded the saliency maps from each method to create binary masks of the top 10% most salient positive voxels within the brain and computed their spatial overlap using the Dice coefficient.

The SmoothGrad analysis produced saliency maps that were in strong quantitative and qualitative (visually) agreement with those generated in the main text, confirming the robustness of our findings. Across all four pathological group comparisons, the two methods identified highly similar anatomical patterns of importance for brain age prediction (**Supplementary Table 16**). At the regional level, we observed excellent concordance, with Spearman’s correlations between the mean saliency values of the two methods ranging from r = 0.90 to 0.93. The sets of the top-20 most salient regions also showed substantial overlap, with Dice coefficients of 0.80 or greater. Finally, at the voxel level, the spatial distribution of the most influential features was highly similar,

with Dice coefficients for the top 10% of voxels ranging from 0.72 to 0.84. This strong cross-method consistency demonstrates that the key anatomical regions highlighted in our main analysis are robust findings and not artifacts of the specific saliency technique employed.

Supplementary Table 16. Quantitative Agreement Between Saliency Mapping Methods. Comparison of vanilla gradient and SmoothGrad saliency maps for each pathology group versus cognitively unimpaired (CU) controls. High agreement is demonstrated by Spearman's correlation of mean ROI saliency, Dice coefficient for the top-20 most salient ROIs, and Dice coefficient for the top 10% most salient voxels.

Pathology Group Comparison	Spearman's *r* (ROI Saliency)	Top-20 ROI Dice	Top 10% Voxel Dice
**AD+LB+ *vs.* CU**	0.932	0.8	0.836
**AD+LB- *vs.* CU**	0.902	0.8	0.812
**AD-LB+ *vs.* CU**	0.922	0.85	0.728
**AD-LB- *vs.* CU**	0.927	0.85	0.723

12. Baseline and longitudinal statistical models

The share notation in the models are as follows:

- **Subjects and visits:** i indexes subjects; t indexes visits. Time t_{it} is years since the subject's first visit for that outcome; thus $t = 0$ at baseline.
- **Group:** Four pathology groups AD-LB-, AD-LB+, AD+LB-, AD+LB+, as well as one CU ADNI longitudinal, with AD-LB- (or CU ADNI longitudinal in BAG analyses) as reference. Indicator variables $G_i^{(g)}$ are 1 if subject i is in group g (non-reference), 0 otherwise.
- **Outcomes:** In the main text, BAG and regional MRI volumes are analyzed both at baseline and longitudinally. Cognitive measures are considered as the outcome in **Supplementary Section 14.**
- **Covariates:** $\mathbf{C}_i$ denotes covariates (continuous covariates centered).
- **Random effects:** Subject-specific random intercept b_{0i} and random slope b_{1i} on time t, with $\begin{bmatrix} b_{0i} \\ b_{1i} \end{bmatrix} \sim \mathcal{N}(\mathbf{0}, \boldsymbol{\Sigma}_b)$. Residuals $\varepsilon_{it} \sim \mathcal{N}(0, \sigma^2)$.
- **Model selection:** For each longitudinal endpoint, fit linear and quadratic time specifications; choose by BIC.

12.1. Baseline models (GLM)

$$\text{Outcome}_{i0} = \beta_0 + \sum_g \gamma_g \, G_i^{(g)} + \boldsymbol{\beta}_C^\top \mathbf{C}_i + \varepsilon_i.$$

12.2. Longitudinal models (LMM)

Let t_{it} denote years since baseline.

- Linear time:

$$\text{Outcome}_{it} = (\beta_0 + b_{0i}) + (\beta_t + b_{1i})\, t_{it} + \sum_g \left[\gamma_g\, G_i^{(g)} + \phi_g \left(t_{it} G_i^{(g)}\right)\right] + \boldsymbol{\beta}_C^{\top} \mathbf{C}_i + \varepsilon_{it}.$$

- Quadratic time (if lower BIC):

$$\text{Outcome}_{it} = \cdots + \beta_{t^2}\, t_{it}^2 + \sum_g \phi_g^{(2)} \left(t_{it}^2 G_i^{(g)}\right) + \varepsilon_{it}.$$

Here, ϕ_g tests group-dependent slope differences; $\phi_g^{(2)}$ tests group-dependent curvature (acceleration/deceleration).

13. Longitudinal effect of co-pathology on region-specific atrophy

Having established elevated baseline BAG and steeper longitudinal BAG increase in co-occurring AD and LB pathologies, we next investigated specific neuroanatomical regions to gain a more granular understanding and clarify how these proteinopathies combine to drive atrophy beyond what either pathology alone might incur. Informed by our saliency map results and prior literature, we focused on the following anatomical targets: (i) the medial temporal lobe (MTL)—aggregating hippocampus, amygdala, entorhinal cortex, and parahippocampal cortex—as these structures are strongly implicated in early AD and exhibited high salience in our interpretability analyses, and (ii) key regions outside the MTL, including the basal ganglia (caudate, putamen, pallidum, accumbens), the middle temporal cortex, and the occipital lobe (lateral occipital, cuneus, pericalcarine, and lingual cortices). The detailed analysis of these regions is included in the main text. In this supplementary section, we provide the similar analyses for additional regions, including the fusiform, cingulum, insula, and MTL sub-regions. The results are presented across the four AD/LB pathological subgroups, offering insights into how co-pathology drives regional atrophy beyond the effects of either pathology alone. Aligned with our global brain age analyses, BIC comparisons consistently supported linear rather than quadratic trajectories in each region of interest (ΔBIC > 0 for all regions, where a negative value indicates preference for the quadratic model, and a positive term indicates preference for the linear model).

Hippocampus.

At baseline, relative to AD-LB-, AD+LB- ($\Delta\beta$ = 403.26, SE = 91.64, p < 0.001) and AD+LB+ ($\Delta\beta$ = 546.11, SE = 109.83, p < 0.001) each showed significantly lower hippocampal volumes, indicating pronounced baseline atrophy. LB positivity alone (AD-LB+) did not differ significantly from AD-LB- (p > 0.05), and pairwise subgroup contrasts indicated that AD+LB+ was also lower

than AD-LB+ ($\Delta\beta = 405.81$, SE = 163.16, $p < 0.05$), whereas AD+LB+ versus AD+LB- remained nonsignificant at baseline. Over time, AD+LB- ($\Delta\beta = 59.53$, SE = 14.92, $p < 0.001$) and AD+LB+ ($\Delta\beta = 98.43$, SE = 21.10, $p < 0.001$) exhibited accelerated atrophy relative to AD-LB-, whereas AD-LB+ again did not reach significance ($p > 0.05$). Pairwise post hoc tests further indicated that AD+LB+ outpaced AD+LB- ($\Delta\beta = 38.90$, SE = 10.81, $p < 0.001$), underscoring a synergistic effect when LB co-occurs with AD.

Entorhinal Cortex.

At baseline, AD+LB+ ($\Delta\beta = 263.01$, SE = 92.53, $p < 0.05$) was significantly lower than AD-LB-, while AD+LB- ($\Delta\beta = 86.13$, SE = 77.20) and AD-LB+ did not differ (both $p > 0.05$). Pairwise contrasts between AD+LB+ and AD+LB- did not meet corrected significance ($p > 0.05$). Over time, AD+LB- ($\Delta\beta = 54.58$, SE = 17.14, $p < 0.01$) and AD+LB+ ($\Delta\beta = 81.69$, SE = 23.71, $p < 0.01$) each displayed faster atrophy than AD-LB-, while AD-LB+ again was not significant ($p > 0.05$). Post hoc tests indicated that AD+LB+ exceeded AD+LB- in slope ($\Delta\beta = 27.11$, SE = 11.38, $p < 0.05$), whereas the contrast versus AD-LB+ did not survive correction.

Amygdala.

At baseline, AD+LB- ($\Delta\beta = 250.53$, SE = 50.13, $p < 0.001$) and AD+LB+ ($\Delta\beta = 388.60$, SE = 60.08, $p < 0.001$) both showed significantly lower amygdala volumes compared to AD-LB-. Pairwise comparisons further indicated that AD+LB+ was lower than AD-LB+ ($\Delta\beta = 339.95$, SE = 89.24, $p < 0.001$) and that AD+LB+ also differed significantly from AD+LB- ($\Delta\beta = 138.08$, SE = 48.01, $p < 0.01$); AD-LB+ also differed from AD+LB- ($\Delta\beta = 201.87$, SE = 83.38, $p < 0.05$). Longitudinally, AD+LB- ($\Delta\beta = 46.13$, SE = 10.08, $p < 0.001$) and AD+LB+ ($\Delta\beta = 76.48$, SE = 14.02, $p < 0.001$) declined faster than AD-LB-, whereas AD-LB+ did not differ significantly ($p > 0.05$). Post hoc tests confirmed that AD+LB+ surpassed AD+LB- in its slope ($\Delta\beta = 30.35$, SE = 6.83, $p < 0.001$), and both AD+LB- and AD+LB+ exceeded AD-LB+ (both $p < 0.01$), demonstrating greater atrophy under co-pathology.

Fusiform.

At baseline, AD+LB- ($\Delta\beta = 518.82$, SE = 191.24, $p < 0.05$) was significantly lower than AD-LB-, while AD+LB+ ($\Delta\beta = 509.61$, SE = 229.20) and AD-LB+ did not differ from AD-LB- after correction (both $p > 0.05$). No other pairwise comparisons reached significance. Over time, AD+LB- ($\Delta\beta = 244.49$, SE = 44.99, $p < 0.001$) and AD+LB+ ($\Delta\beta = 367.45$, SE = 62.95, $p < 0.001$) each showed accelerated atrophy relative to AD-LB-. Pairwise tests also revealed that AD+LB+ exceeded AD+LB- ($\Delta\beta = 122.96$, SE = 32.46, $p < 0.001$), mirroring the synergy noted in other regions; LB positivity alone (AD-LB+) did not differ significantly from AD-LB- ($p > 0.05$).

Cingulum.

At baseline, no differences were observed between AD-LB- and AD-LB+ ($p > 0.05$); however, AD+LB- ($\Delta\beta = 469.31$, SE = 183.83, $p < 0.05$) and AD+LB+ ($\Delta\beta = 689.11$, SE = 220.32, $p < 0.05$) were each significantly lower than AD-LB-. Over time, AD+LB- ($\Delta\beta = 109.71$, SE = 34.25, $p < 0.01$) and AD+LB+ ($\Delta\beta = 193.32$, SE = 49.05, $p < 0.001$) both declined faster than AD-LB-. Pairwise contrasts confirmed that AD+LB+ also surpassed AD-LB+ ($\Delta\beta = 172.17$, SE = 53.39, $p < 0.01$) and AD+LB- ($\Delta\beta = 83.61$, SE = 26.75, $p < 0.01$), consistent with the co-pathology effect.

Insula.

No subgroup differed from AD-LB- at baseline after correction (all $p > 0.05$). By contrast, over time, AD+LB- ($\Delta\beta = 86.01$, SE = 26.73, $p < 0.01$) and AD+LB+ ($\Delta\beta = 137.52$, SE = 39.38, $p < 0.01$) each had a significantly faster slope than AD-LB-, whereas AD-LB+ remained nonsignificant ($p > 0.05$). Pairwise post hoc tests indicated that both AD+LB- and AD+LB+ also exceeded AD-LB+ (both $p < 0.05$). Moreover, AD+LB+ differed significantly from AD+LB- ($\Delta\beta = 51.51$, SE = 23.86, $p < 0.05$).

In sum, these longitudinal volumetric findings continue to demonstrate that LB pathology, when added to an existing AD pathological process, exerts a synergistic effect on neurodegeneration. Whereas isolated LB status often yields nonsignificant effects, co-pathology (AD+LB+) consistently intensifies atrophy—now evident across multiple regions both at baseline (e.g., hippocampus; cingulum; fusiform [AD+LB-]) and over time—aligning with our brain-age–based analyses and highlighting the augmented neuroanatomical burden imposed by dual pathology.

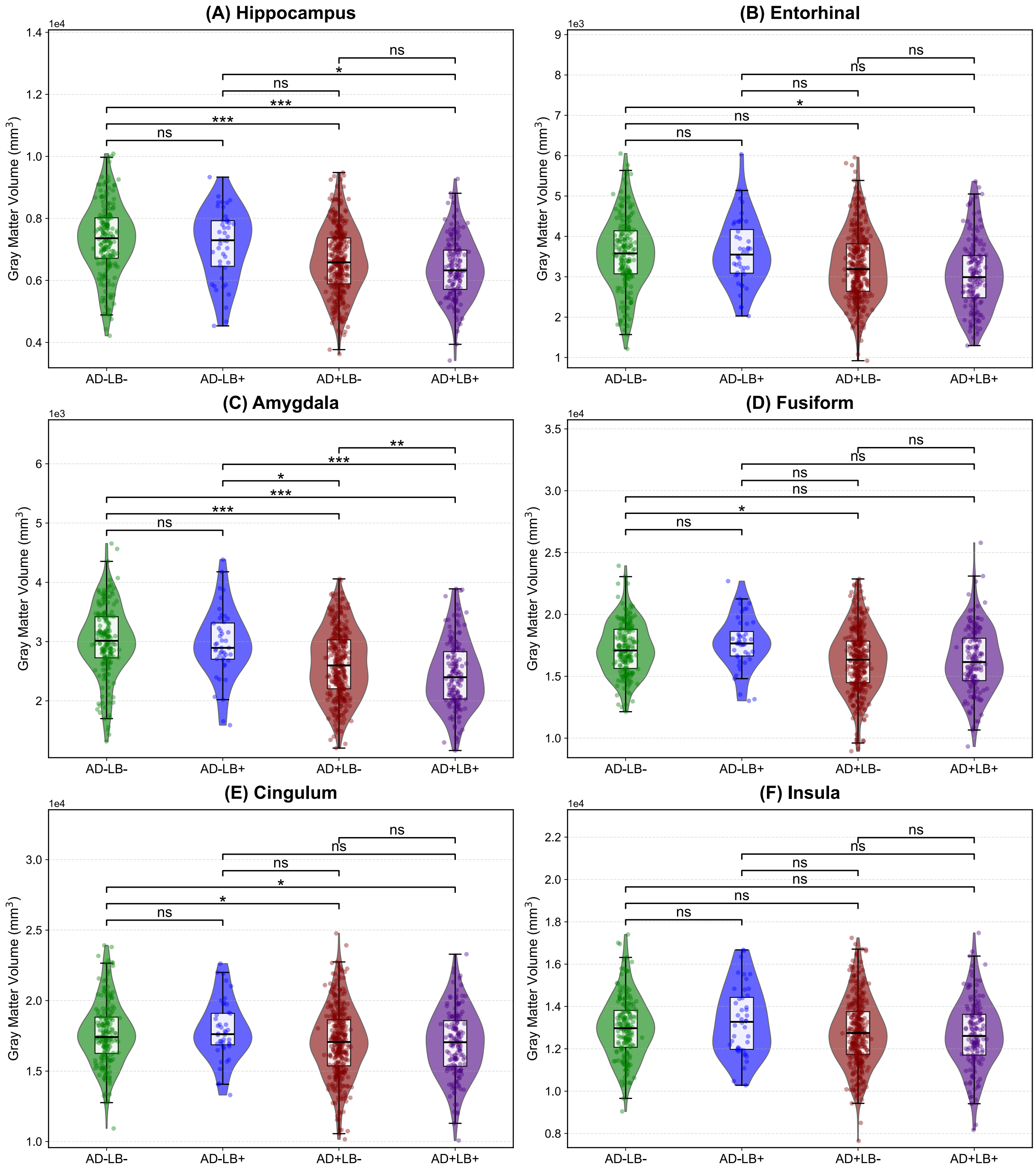

Supplementary Figure 5. Baseline volumetric comparisons across AD/LB pathology subgroups. Violin plots illustrate the distribution of gray matter volume at baseline across four AD/LB pathological subgroups for **(A)** Hippocampus, **(B)** Entorhinal cortex, **(C)** Amygdala, **(D)** Fusiform gyrus, **(E)** Cingulum, and **(F)** Insular cortex, with overlaid boxplots indicating median and interquartile ranges. The top bars and corresponding significance labels reflect pairwise group comparisons under a general linear model adjusted for baseline age, sex, baseline cognitive state, APOE-ε4 status, years of education, recruitment site, and ICV, with p-values corrected for multiple comparisons. (*ns*: not significant; *$p < 0.05$; **$p < 0.01$; ***$p < 0.001$).

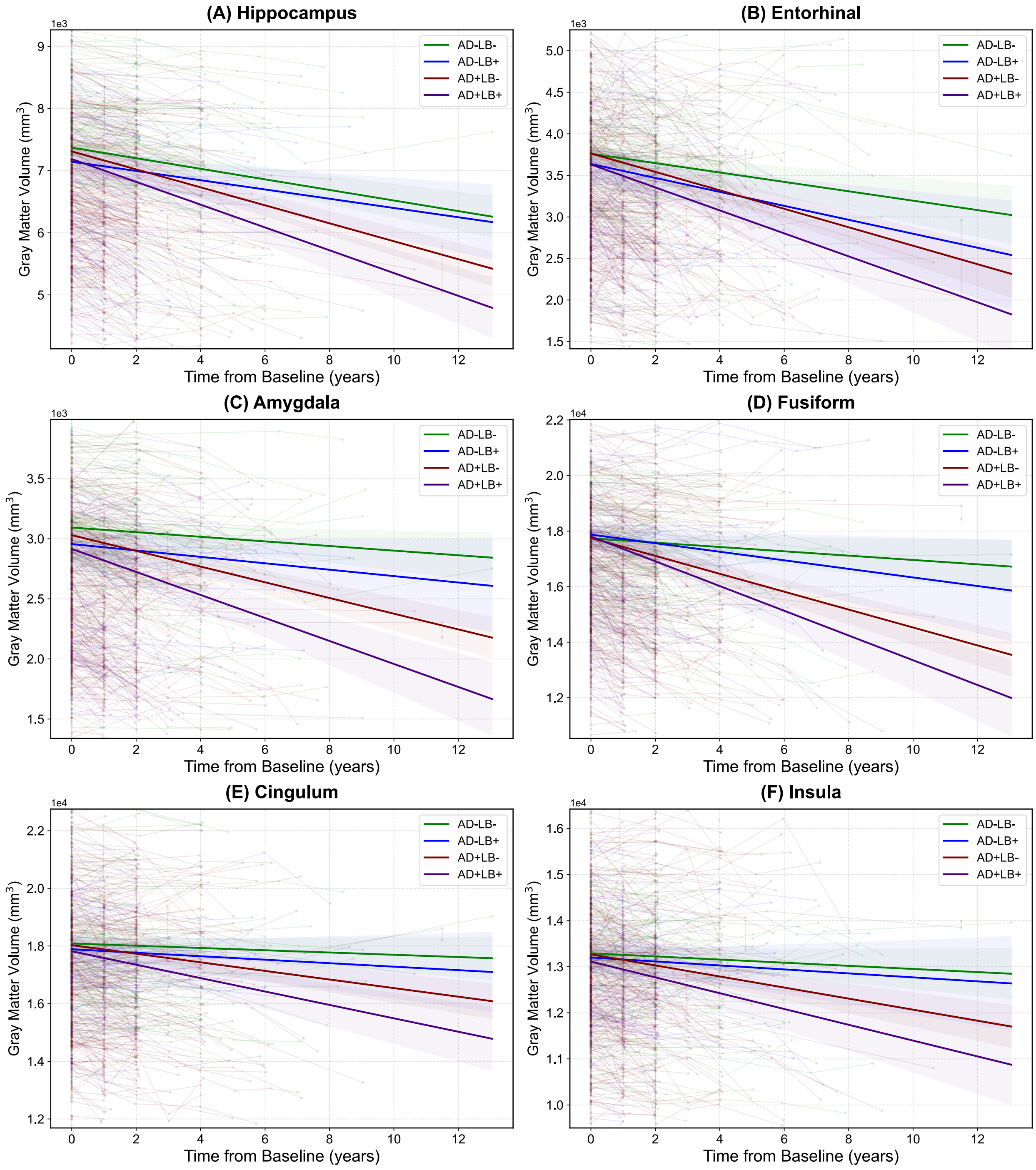

Supplementary Figure 6. **Extended longitudinal analyses of volumetric atrophy.** Each panel shows gray matter volumes (y-axis) over time (x-axis) for **(A)** Hippocampus, **(B)** Entorhinal, **(C)** Amygdala, **(D)** Fusiform, **(E)** Cingulum, and **(F)** Insular cortex across the four AD/LB pathological subgroups. Each thin line represents an individual's change in gray matter volume over time from baseline (spaghetti plots, included only for visual context), while the thicker lines with shaded bands depict population-level trajectories predicted from the fitted linear mixed-effects models (random intercepts and slopes; fixed effects as a function of time) and corresponding 95% confidence intervals. In all regions, AD+LB+

demonstrates the steepest decline relative to AD+LB-, AD-LB+, and AD-LB- ($p < 0.001$–0.05 across contrasts), consistent with a faster and more pronounced atrophy pattern under LB co-pathology. All models are adjusted for baseline age, sex, baseline cognitive status, APOE-ε4 status, years of education, recruitment site, and ICV, with all results corrected for multiple comparisons.

14. Longitudinal effect of co-pathology on cognition

Building on the structural and saliency-map findings, we next examined whether LB co-pathology similarly accelerates longitudinal cognitive decline. To this end, we conducted mixed-effects analyses on two global cognition measures—CDR Sum of Boxes (CDRSB) and the ADAS-Cog 11 (ADAS11)—as well as four domain-specific cognitive composites (memory, language, visuospatial, and executive functioning). All baseline group differences for each outcome were examined using a general linear model.

For CDRSB, a quadratic model was preferred (ΔBIC = -8.78). As shown in **Supplementary Figure 7A**, at baseline, none of the three pathology subgroups (AD+LB-, AD-LB+, AD+LB+) differed significantly from AD-LB- after multiple-comparison correction (all $p > 0.05$). However, over time, the non-linear (Time^2) term emerged as the key driver of group differences (**Supplementary Figure 8A**). Although AD+LB- showed a modestly positive Time^2 component ($\beta = 0.025$, SE = 0.016, $p > 0.05$), AD+LB+ exhibited a significantly larger acceleration ($\beta = 0.116$, SE = 0.027, $p < 0.001$) relative to AD-LB-. Pairwise comparisons further revealed that this Time^2 effect in AD+LB+ significantly exceeded that of AD+LB- ($\beta = 0.091$, $p < 0.001$). A similar pattern emerged for ADAS11, which also favored a quadratic model (ΔBIC = -8.41). As shown in **Supplementary Figure 7B**, at baseline, AD+LB- ($\beta = 1.42$, SE = 0.41, $p < 0.001$) and AD+LB+ ($\beta = 2.73$, SE = 0.55, $p < 0.001$) both had significantly higher ADAS11 deficits than AD-LB-, and pairwise comparisons indicated that AD+LB+ also exceeded AD-LB+ ($p < 0.01$) and AD+LB- ($p < 0.05$). Over time (**Supplementary Figure 8B**), while AD+LB- showed a mild positive Time^2 ($\beta = 0.022$, SE = 0.008, $p < 0.05$), AD+LB+ displayed a more pronounced acceleration ($\beta = 0.54$, SE = 0.14, $p < 0.001$) relative to AD-LB-, and also declined more steeply than AD+LB- ($\beta = 0.32$, $p < 0.01$). These results align with our saliency and volumetric findings showing pronounced medial temporal and subcortical atrophy in AD+LB+ relative to AD+LB-, suggesting that α-synuclein aggregates intensify the atrophic processes already triggered by AD, causing more global clinical severity.

Among the domain-specific composites, we observed a similar pattern of intensified decline in AD+LB+ (**Supplementary Figure 7C–F** for baseline, and **Supplementary Figure 8C–F** for longitudinal). Based on the BIC values, linear model fits best for the memory (ΔBIC = 0.84)

and visuospatial composites (ΔBIC = 19.87), whereas language (ΔBIC = -8.47) and executive function (ΔBIC = -5.54) followed quadratic trajectories. In the memory domain, at baseline, both AD+LB- ($\beta = -0.485$, $p < 0.001$) and AD+LB+ ($\beta = -0.630$, $p < 0.001$) were significantly lower than AD-LB-, and additional pairwise tests indicated that AD+LB+ also scored lower than AD+LB- ($p < 0.05$) and AD-LB+ ($p < 0.001$). Over time, both AD+LB- and AD+LB+ declined significantly faster than AD-LB- ($p < 0.001$ for each), with AD+LB+ again showing the steepest slope (*vs.* AD+LB-: $\beta = -0.13$, $p < 0.01$). These findings mirror the marked medial temporal lobe atrophy (especially in the hippocampus and entorhinal cortex) observed in our structural analyses. For language, AD+LB- ($\beta = -0.203$, $p < 0.05$) and AD+LB+ ($\beta = -0.294$, $p < 0.01$) were both significantly worse than AD-LB- at baseline, whereas AD-LB+ was not ($p = 0.576$). No other baseline pairwise contrasts reached significance in language ($p > 0.05$). Over time, both AD+LB- and AD+LB+ exhibited accelerated declines ($p < 0.05$ and < 0.001, respectively), with the strongest acceleration evident in AD+LB+ ($\beta = -0.022$, $p < 0.01$), aligning with the saliency signals in mid-temporal, and fusiform areas. Additionally, AD+LB+ versus AD+LB- in language slope reached significance ($p < 0.05$), confirming a steeper decline under co-pathology. In visuospatial, although baseline differences were less pronounced (i.e., AD+LB- ($\beta = -0.141$, $p > 0.05$) and AD+LB+ ($\beta = -0.227$, $p > 0.05$) were borderline lower than AD-LB-, and no other pairwise tests were significant at baseline), both AD+LB- and AD+LB+ declined faster than AD-LB- ($p < 0.001$, $p < 0.001$, respectively), with co-pathology showing the steepest slope (β *vs.* AD+LB- = -0.16, $p < 0.001$), consistent with the occipital and mid-temporal saliency increases. A similar pattern emerged for executive function. At baseline, AD+LB- ($\beta = -0.343$, $p < 0.001$) and AD+LB+ ($\beta = -0.554$, $p < 0.001$) had a significantly pronounced deficit compared to AD-LB-, and pairwise tests also revealed that AD+LB+ was lower than AD+LB- ($p < 0.05$) and AD-LB+ ($p > 0.05$). Over follow-up, both AD+LB- and AD+LB+ deteriorated faster than AD-LB- (both $p < 0.05$), and AD+LB+ again exceeded AD+LB- ($\beta = -0.005$, $p < 0.05$). These results align with the elevated saliency near the cholinergic nuclei, particularly evident when comparing AD+LB+ to AD+LB-, indicating that LB pathology in the basal forebrain may exacerbate the attentional and executive deficits that already characterize AD.

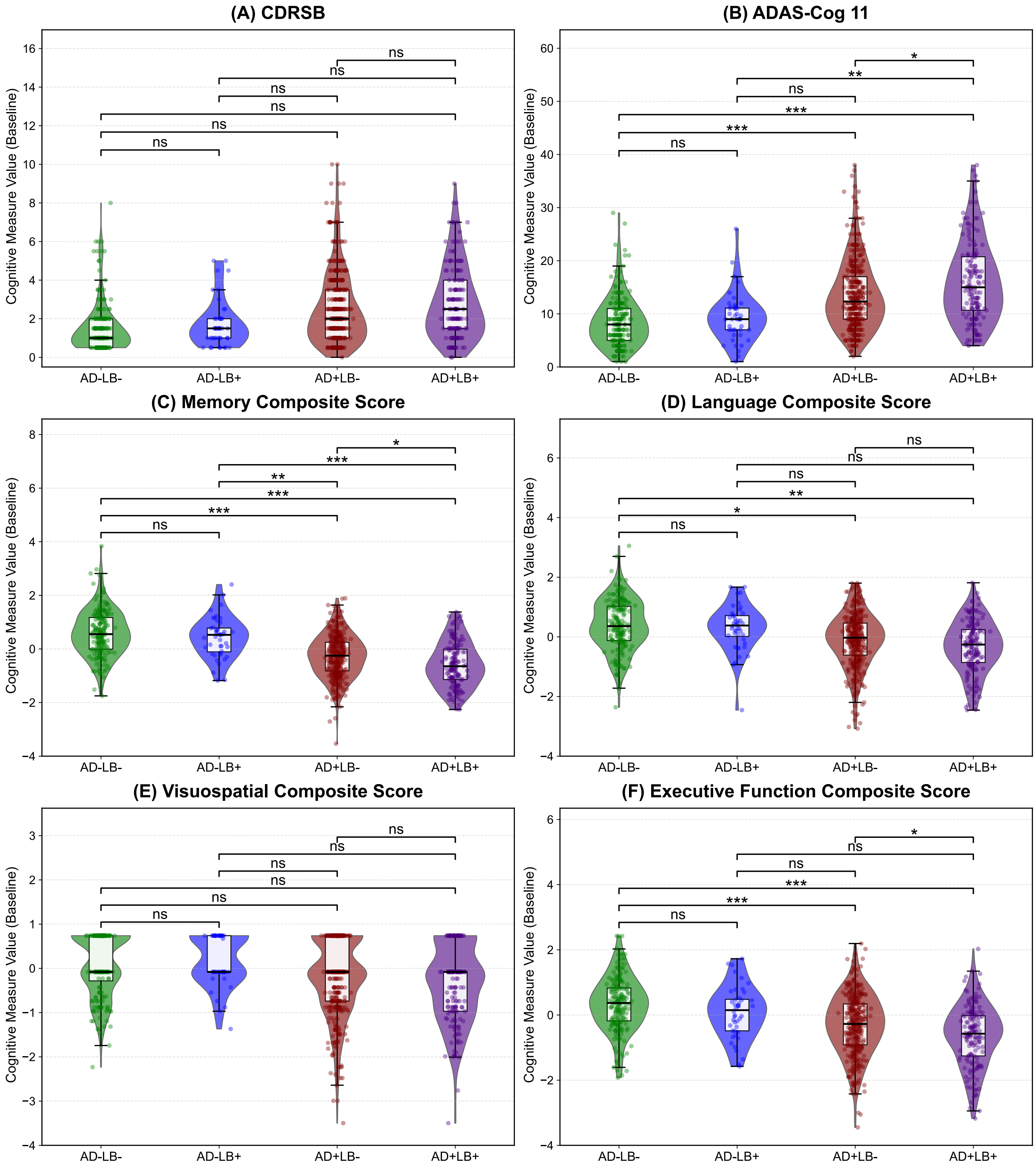

Supplementary Figure 7. Baseline comparisons of global and domain-specific cognitive measures across AD/LB subgroups. **(A)** CDR Sum of Boxes (CDRSB) and **(B)** ADAS-Cog 11 (ADAS11) illustrate global cognitive measures; and **(C–F)** show the domain-specific performance: Memory, Language, Visuospatial, and Executive composites, respectively. Each violin plot depicts the distribution of the cognitive measure at baseline, with boxplots overlaid to illustrate medians and quartiles. The top bars and corresponding significance labels reflect pairwise group comparisons under a general linear model adjusted

for baseline age, sex, baseline cognitive state, APOE-ε4 status, years of education, and recruitment site with p-values corrected for multiple comparisons. (*ns*: not significant; *$p < 0.05$; **$p < 0.01$; ***$p < 0.001$).

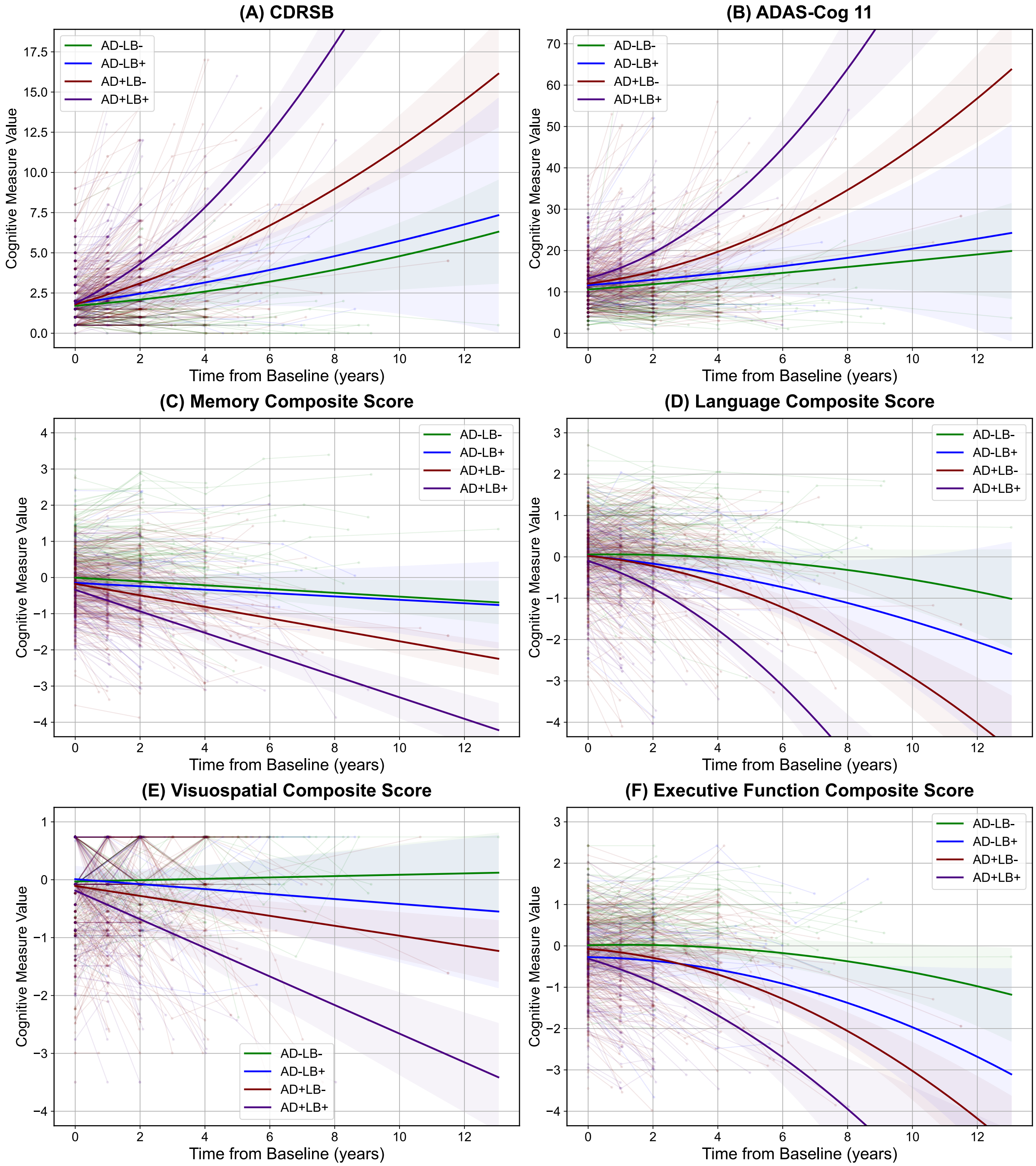

Supplementary Figure 8. Longitudinal trajectories of global and domain-specific cognitive measures across AD/LB subgroups. (A) CDR Sum of Boxes (CDRSB) and **(B)** ADAS-Cog 11 (ADAS11) illustrate global cognitive decline over time; **(C–F)** show the domain-specific performance: Memory, Language, Visuospatial, and Executive composites, respectively. Thin lines represent each individual's time course

(spaghetti plots, included only for visual context), whereas thicker lines with shaded areas show population-level trajectories predicted from the fitted mixed-effects models (random intercepts and slopes; fixed effects as a function of time), with model form (linear or quadratic) selected via the Bayesian Information Criterion (BIC), and corresponding 95% confidence intervals. Notably, the co-pathology subgroup (AD+LB+) exhibits the steepest acceleration (for quadratic trajectories) or slope (for linear trajectories) of decline in both global and domain-specific outcomes. Specifically, *vs.* AD+LB-, AD+LB+ shows significantly greater worsening in CDRSB ($p < 0.001$), ADAS11 ($p < 0.01$), Memory ($p < 0.01$), Language ($p < 0.01$), Visuospatial ($p < 0.001$), and Executive function ($p < 0.05$). These findings indicate that LB co-pathology amplifies and hastens the deterioration already driven by AD-specific pathologies. All models are corrected for baseline age, sex, baseline cognitive state, APOE-ε4 status and level of education. Also, all p-values are adjusted for multiple comparison.

15. Incremental value of BAG beyond conventional volumetrics

In this section, we tested whether BAG captures cognitive impairment and longitudinal cognitive decline beyond standard covariates ("BASE") and two commonly used structural MRI approaches: (1) a whole-brain atrophy burden score derived from ROI-wise normative atrophy z-scores ("$\text{SimpleAtrophy}_{\text{total,z}}$"), and (2) conventional FreeSurfer volumetric measures targeting medial temporal lobe volume and global cortical gray matter volume ("VOL" = MTL + CortexGM). In other words, we asked whether BAG provides incremental prognostic information for change in global scales and cognitive domains over time beyond established structural MRI summaries. To evaluate this, we compared model fits and time slopes using baseline regression and linear mixed-effects models and reported ΔBIC (negative favors the augmented model) and likelihood-ratio test (LRT) p-values as formal evidence.

MRI predictors are defined below:

1) BAG: BAG was the bias-corrected brain age gap from the deep learning age model. For interpretability across predictors, BAG was z-scored.

2) Whole-brain normative atrophy burden (SimpleAtrophy_total,z): Using all available FreeSurfer gray-matter ROIs (within DK parcellation), we fit robust regression models in cognitively unimpaired (CU) baseline scans for each ROI *i*:

$$Y_i = \beta_{0,i} + \beta_{1,i}\,\text{Age} + \beta_{2,i}\,\text{Sex} + \beta_{3,i}\,\text{eTIV} + \beta_{4,i}\,\text{Site} + \varepsilon_i,$$

where Y_i is the ROI volume (cortical gray matter volume for DK parcels; aseg volume for subcortical). We estimated the residual scale σ_i from the fitted model. For any scan of subject s at visit t, we computed the residual $\hat{r}_{i,s,t} = Y_{i,s,t} - \hat{Y}_{i,s,t}$ and defined the atrophy z-score:

$$Z_{i,s,t}^{\text{atrophy}} = -\frac{\hat{r}_{i,s,t}}{\sigma_i},$$

so larger values indicate *smaller than expected* gray matter volume (i.e., more atrophy).

For each visit, we aggregated Z_i^{atrophy} across the ROI set to obtain a single *SimpleAtrophy*$_{total}$ score. Our primary analysis was the *mean* across ROIs; we then z-scored it relative to CU to obtain SimpleAtrophy$_{total,z}$. Alternative aggregators (median, trimmed/winsorized mean, burden metrics, top-k) yielded the same qualitative model ranking.

3) Conventional volumetrics: In parallel, we defined a conventional volumetric feature set ("VOL") using widely used FreeSurfer summary measures: (i) a bilateral MTL composite volume and (ii) global cortical gray matter volume (CortexGM). For interpretability aligned with BAG/SimpleAtrophy, volumetric predictors were standardized and sign-coded so higher values correspond to smaller volume (i.e., greater atrophy).

Statistical models and comparisons:

Outcomes: Global measures (CDR-SB, ADAS-Cog 11) and cognitive domain composites (Memory, Language, Visuospatial, Executive).

Covariates: Baseline age, sex, education, baseline cognitive state (DX_bl), APOE-ε4, site, and intracranial volume (ICV). Group is pathology category (reference AD−LB−). Longitudinal models additionally include time and time×Group.

We evaluated the same candidate MRI summaries at both baseline and longitudinal levels:

Baseline (first available visit; OLS):

- Base: outcome ~ Group + covariates
- Base + BAG
- Base + SimpleAtrophy_total,z
- Base + VOL
- Base + VOL + BAG (tests BAG incremental value beyond conventional volumes)

Longitudinal (LMM):

- Base: outcome ~ time×Group + covariates + (1 + time | subject)
- Base + BAG + time×BAG
- Base + SimpleAtrophy_total,z + time×SimpleAtrophy_total,z
- Base + VOL + time×VOL
- Base + VOL + BAG (+ time×BAG and time×VOL)

Model preference is judged by ΔBIC (negative values favor the augmented model) and likelihood-ratio test (LRT) p-values.

The ΔBIC quantities reported below address related but distinct comparison questions. "ΔBIC (Simple − Base)" and "ΔBIC (BAG − Base)" quantify improvement over the covariate-only baseline model when adding either SimpleAtrophy or BAG, respectively. "ΔBIC (BAG − Simple)"

compares two alternative MRI summary models directly and indicates whether the BAG-based model is preferred over the whole-brain SimpleAtrophy model. On the other hand, "ΔBIC add BAG|VOL" is a nested incremental comparison defined as [BIC(Base+VOL+BAG) − BIC(Base+VOL)], and tests whether BAG adds explanatory value after conventional volumetric markers (MTL + CortexGM) are already included. Thus, the first three ΔBIC columns compare alternative model specifications, whereas the final column specifically tests incremental value of BAG beyond conventional volumetrics.

Results for all cognitive measures are summarized in **Supplementary Table 17A (baseline)** and **Supplementary Table 17B (longitudinal)**. All LRT p-values were <0.001 unless otherwise noted.

Baseline cognition: Across outcomes, BAG improved model fit relative to the Base model and generally outperformed the whole-brain SimpleAtrophy summary. When evaluated against conventional volumetrics (MTL + CortexGM), adding BAG further improved model fit for most outcomes (**Supplementary Table 17A**). All nested likelihood ratio tests were $p < 0.001$, except the baseline comparisons for the VOL models where p-values ranged from 0.011 to 0.004.

Longitudinal cognitive trajectories: In longitudinal mixed-effects models, BAG consistently improved model fit relative to Base and SimpleAtrophy across all outcomes (**Supplementary Table 17B**). Crucially, when BAG was added to models already including MTL and global cortical GM volume, model fit improved substantially for every outcome. All nested likelihood ratio tests for these comparisons were $p < 0.001$. These findings indicate that BAG captures variance in longitudinal cognitive decline beyond both whole-brain normative atrophy summaries and conventional volumetric markers.

Sensitivity analysis: When analyses were restricted to AD+ participants (AD+LB- vs AD+LB+), the same qualitative pattern was observed: BAG improved model fit relative to both SimpleAtrophy and VOL comparators for baseline cognition and longitudinal trajectories.

Together, the results show that BAG consistently improves cognitive model fit beyond two different classes of structural MRI summaries:

- Whole-brain normative atrophy burden derived from ROI-wise z-scores.
- Conventional volumetric markers capturing medial temporal and global cortical atrophy.

This convergence across analytic frameworks supports BAG as a compact nonlinear summary of distributed neurodegenerative burden that provides incremental information beyond established structural MRI measures. Accordingly, these analyses support BAG as an integrated MRI

summary of distributed structural burden, especially for longitudinal cognitive decline, rather than implying that BAG indexes a biological process wholly distinct from established atrophy patterns.

Supplementary Table 17A. Baseline model comparisons (OLS).

Outcome	ΔBIC (Simple − Base)	ΔBIC (BAG − Base)	ΔBIC (BAG − Simple)	ΔBIC add BAG|VOL
**CDRSB**	-18.4	-25.1	-6.7	-2.8
**ADAS-Cog 11**	-26.2	-33.0	-6.8	-1.6
**Memory**	-15.7	-20.5	-4.8	-0.59
**Language**	-20.8	-28.9	-8.1	-11.0
**Visuospatial**	-19.6	-23.7	-4.1	-2.7
**Executive**	-21.3	-32.4	-11.1	-22.9

Negative ΔBIC favors the augmented model.
"ΔBIC add BAG|VOL" is BIC(Base+VOL+BAG)−BIC(Base+VOL).

Supplementary Table 17B. Longitudinal model comparisons (LMM).

Outcome	ΔBIC (Simple − Base)	ΔBIC (BAG − Base)	ΔBIC (BAG − Simple)	ΔBIC add BAG|VOL
**CDRSB**	-50.8	-68.3	-17.5	-36.5
**ADAS-Cog 11**	-78.4	-88.9	-10.5	-46.5
**Memory**	-43.1	-70.9	-27.8	-45.3
**Language**	-64.7	-92.9	-28.2	-56.6
**Visuospatial**	-65.3	-71.9	-6.6	-25.7
**Executive**	-57.8	-104.1	-46.3	-76.4

Negative ΔBIC favors the augmented model.
"ΔBIC add BAG|VOL" is BIC(Base+VOL+BAG)−BIC(Base+VOL).

16. Details of the mediation analysis

Mediation analyses focused on the contrast LB+ *vs.* LB- among AD+ participants (indicator X), using the first available visit for baseline and all visits for longitudinal. Time was measured in years since each participant's baseline and centered. Let X_i denote LB positivity (1 for LB+, 0 for LB-) among AD-positive participants, M_i the mediator (brain age gap, BAG; higher indicates an older-appearing brain), Y_{it} repeated cognitive scores for participant i at time t (years since baseline), and C_i the list of covariates.

We report the average causal mediation effect (ACME; indirect effect), the average direct effect (ADE), and the total effect (TE), together with the proportion mediated (PM) defined as

ACME/TE. The ACME is *ab*, the ADE is *c′*, and the TE is *c*. Inference used non-parametric bootstrap with 5,000 resamples and bias-corrected and accelerated (BCa) confidence intervals for the ACME. Two-sided p-values for the ACME were derived from the bootstrap sign distribution. The proportion mediated was computed as the point estimate ab/c. As a sensitivity analysis, we additionally included X×M in the outcome model; ACME estimates and inferences were unchanged. Because ratios can be unstable when the total effect is small or when the indirect and total effects have opposite signs, we flag PM in two situations: $|c| < max(0.05 \cdot SD(Y), 1.0 \cdot SE(c))$, and when ACME and TE have opposite signs. False discovery rate control used the Benjamini–Hochberg procedure within each prespecified analysis family. Models used all available observations for which X, M, Y and C were observed.

Below, we present the full results of the mediation analyses investigated in the main text. We formally tested whether BAG mediates the association between LB positivity within AD+ and cognition. At baseline, LB+ was strongly associated with higher BAG (path a), and BAG in turn related to poorer performance on the four cognitive composites (negative path b) and greater impairment on the global clinical scales (positive path b), consistent with their scoring directions. Across all six outcomes, the indirect effect (a·b) was statistically significant with BCa confidence intervals excluding zero and remained significant after FDR control; proportions mediated (PM) were largest for the global clinical measures (CDRSB ≈ 84%; ADAS-Cog 11 ≈ 41%) and ranged from ~38–59% for the domain composites. For Language and Visuospatial, the direct effect (c′) was not significant, consistent with near-complete mediation at baseline; for Memory and Executive, c′ remained significant but attenuated relative to the total effect (c), indicating partial mediation. Notably, for CDRSB the total effect (c) was modest and not significant whereas a·b was robust, a pattern compatible with an “indirect-only” pathway when the net total signal is small. Together, these results indicate that cross-sectionally a substantial fraction of the LB+–cognition association is conveyed through BAG, with the strongest mediation for clinical severity and language/visuospatial domains (**Supplementary Figure 9**).

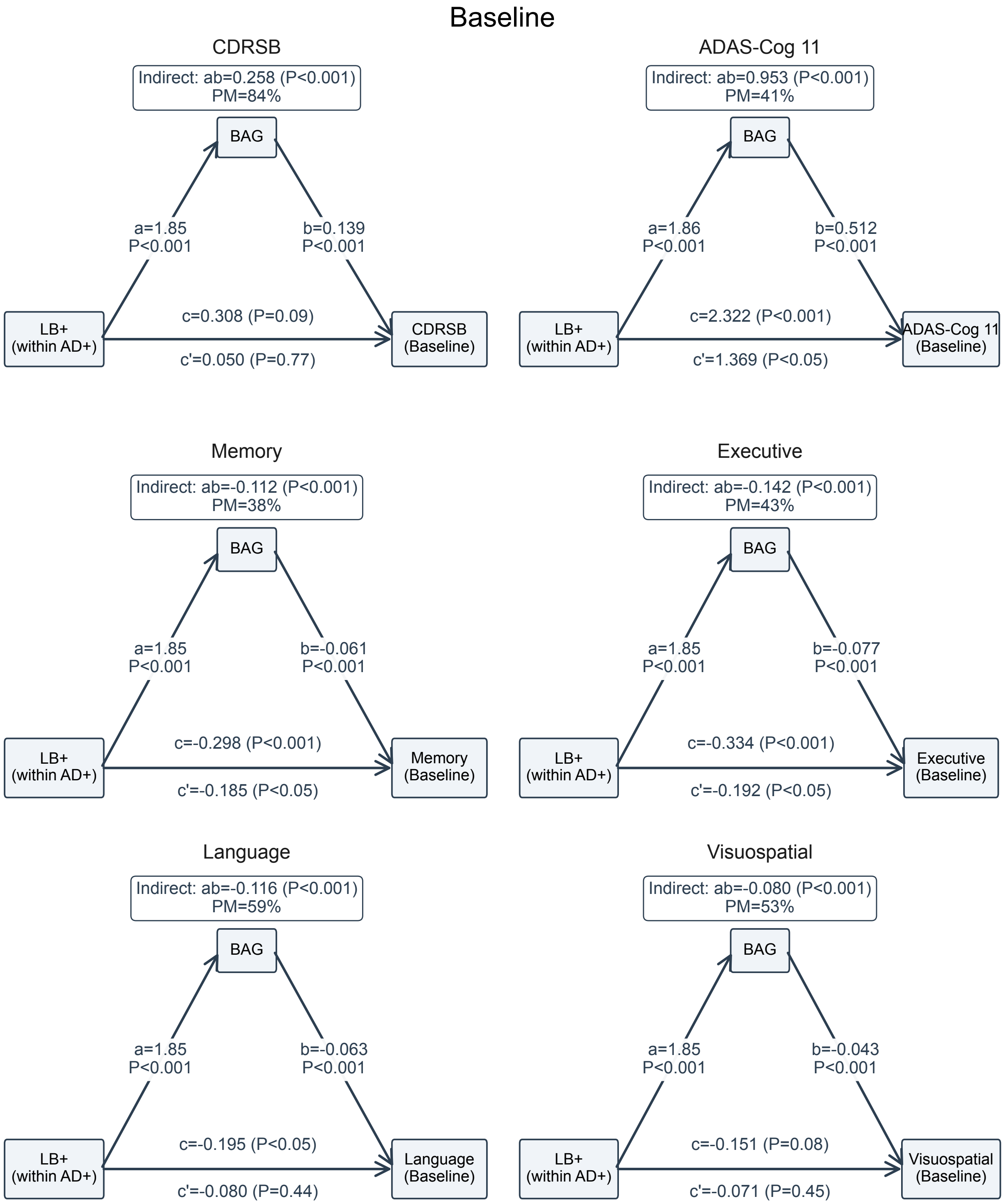

Supplementary Figure 9. Mediation of baseline cognition by brain age gap. Mediation models testing whether the effect of LB positivity within AD+ individuals on baseline cognition is conveyed through brain age gap (BAG). Path coefficients are shown for the indirect pathway (a·b), the total effect (c), and the direct effect (c′) for global clinical outcomes (CDRSB, ADAS-Cog 11) and each domain (Memory, Executive, Language, Visuospatial). Effects were directionally consistent with scoring (negative b for cognitive composites; positive b for impairment scales). The indirect effect was significant for all outcomes by BCa bootstrap and remained significant after FDR control; the proportion mediated ranged from ~38% to 84%

(largest for CDR-SB), indicating that BAG accounts for a substantial share of the LB+–outcome association at baseline. Direct effects were attenuated and not significant for Language and Visuospatial, consistent with near-complete mediation; Memory, Executive and ADAS-Cog 11 showed partial mediation (c′ reduced but non-zero).

We next evaluated mediation for longitudinal change (Baseline-to-Change; **Supplementary Figure 10**) by pairing baseline BAG with rates of cognitive change. Again, a·b was significant for all outcomes after FDR control, but the mediated fraction was smaller (PM ≈ 14–39%), as expected when modeling slopes rather than levels. For CDRSB, c′ was reduced and compatible with zero at the chosen threshold (P≈0.06), whereas for ADAS-Cog 11 the direct path remained significant but attenuated, indicating near-complete versus partial mediation, respectively. The cognitive composites showed consistent, directionally appropriate effects (negative b for decline in Memory, Executive, Language, and Visuospatial), with significant a·b and attenuated c′, supporting partial mediation of LB-related acceleration of decline by BAG. Overall, longitudinal analyses corroborate the baseline pattern: BAG carries a meaningful share of the LB+ effect on cognitive trajectories within AD+, with the clearest mediation for clinical worsening and smaller—but still significant—mediation for domain-specific decline.

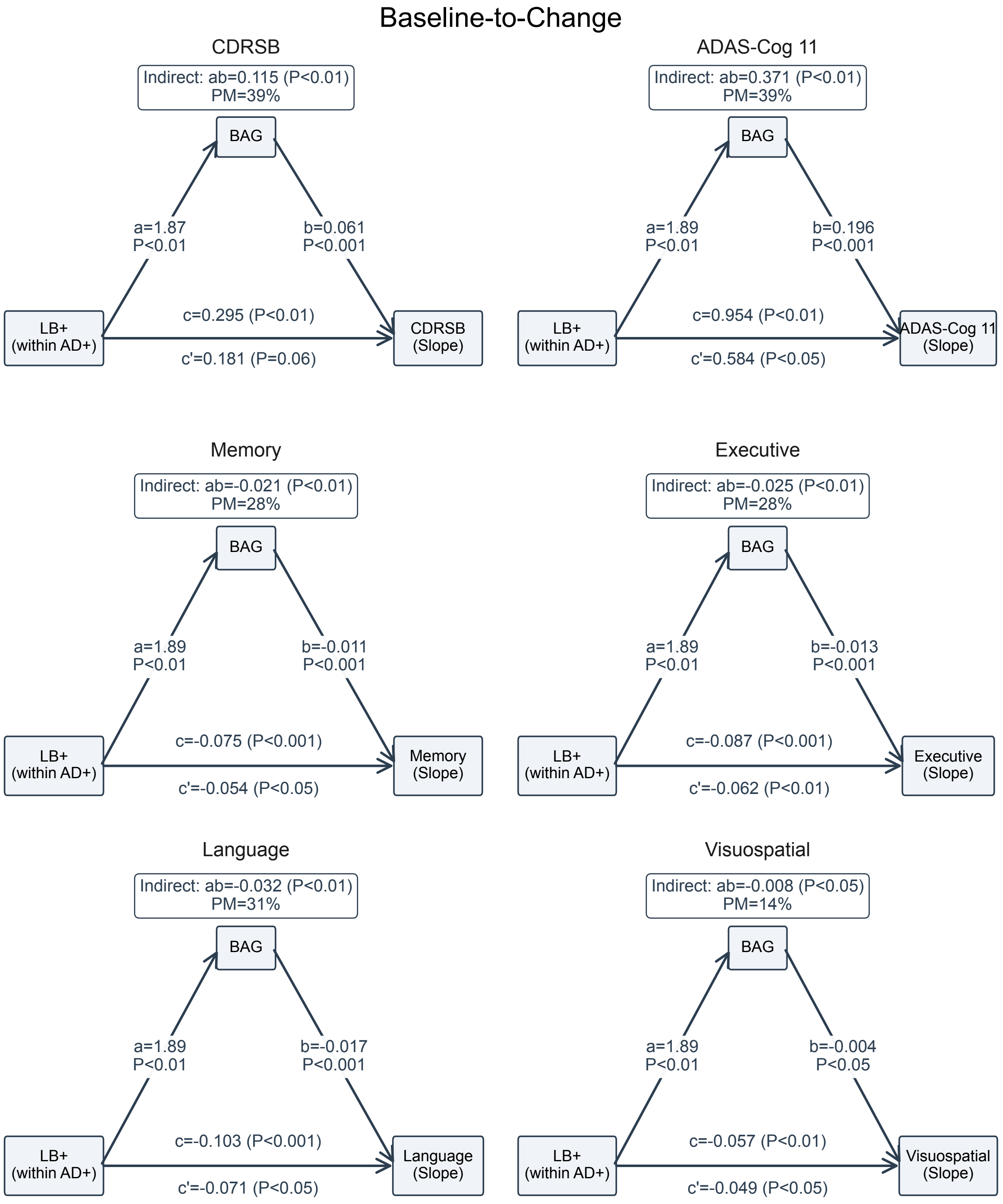

Supplementary Figure 10. Mediation of longitudinal cognitive change by brain age gap. Mediation models examining whether BAG mediates the association between LB positivity within AD+ individuals and trajectories of cognitive decline. Path coefficients (a, b, c, c′) and indirect effects (ab) are displayed for each outcome. Indirect effects were significant across all outcomes (BCa bootstrap; FDR-controlled), with proportion mediated values between ~14% and 39%, indicating that BAG partly explains the impact of LB co-pathology on cognitive trajectories. Directionality matched expectations (negative b for domain composites; positive b for impairment scales). Direct effects were reduced relative to the total effect,

including near-zero c' for CDR-SB (consistent with near-complete mediation), whereas other outcomes showed partial mediation.

References:

1. Biondo F, Jewell A, Pritchard M, et al. Brain-age is associated with progression to dementia in memory clinic patients. *Neuroimage-Clin*. 2022;36doi:10.1016/j.nicl.2022.103175

2. Pomponio R, Erus G, Habes M, et al. Harmonization of large MRI datasets for the analysis of brain imaging patterns throughout the lifespan. *Neuroimage*. Mar 2020;208doi:10.1016/j.neuroimage.2019.116450

3. Fortin JP, Cullen N, Sheline YI, et al. Harmonization of cortical thickness measurements across scanners and sites. *Neuroimage*. Feb 15 2018;167:104-120. doi:10.1016/j.neuroimage.2017.11.024

4. Fortin JP, Sweeney EM, Muschelli J, Crainiceanu CM, Shinohara RT, Neuroimaging AD. Removing inter-subject technical variability in magnetic resonance imaging studies. *Neuroimage*. May 15 2016;132:198-212. doi:10.1016/j.neuroimage.2016.02.036

5. Smilkov D, Thorat N, Kim B, Viégas F, Wattenberg M. Smoothgrad: removing noise by adding noise. *arXiv preprint*. 2017;doi:10.48550/arXiv.1706.03825
